# Cu_4_S Cluster in “0-Hole” and “1-Hole” States: Geometric and Electronic Structure Variations for the Active Cu_Z_* Site of N_2_O Reductase

**DOI:** 10.1021/jacs.3c04893

**Published:** 2023-08-11

**Authors:** Yang Liu, Sayanti Chatterjee, George E. Cutsail, Sergey Peredkov, Sandeep K. Gupta, Sebastian Dechert, Serena DeBeer, Franc Meyer

**Affiliations:** † Institute of Inorganic Chemistry, University of Göttingen, Tammannstraße 4, 37077 Göttingen, Germany; ‡ Max Planck Institute for Chemical Energy Conversion, Stiftstrasse 34−36, 45470 Mülheim an der Ruhr, Germany; § Institute of Inorganic Chemistry, University of Duisburg-Essen, Universitätsstraße 7, 45117 Essen, Germany; ⊥ International Center for Advanced Studies of Energy Conversion (ICASEC), University of Göttingen, Tammannstraße 6, 37077 Göttingen, Germany

## Abstract

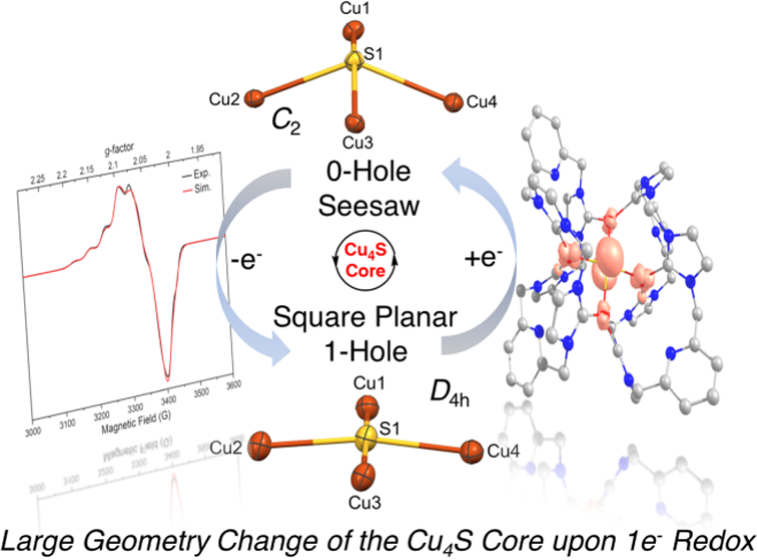

The active site of nitrous oxide reductase (N_2_OR), a key enzyme in denitrification, features a unique μ_4_-sulfido-bridged tetranuclear Cu cluster (the so-called Cu_Z_ or Cu_Z_* site). Details of the catalytic mechanism have remained under debate and, to date, synthetic model complexes of the Cu_Z_*/Cu_Z_ sites are extremely rare due to the difficulty in building the unique {Cu_4_(μ_4_-S)} core structure. Herein, we report the synthesis and characterization of [Cu_4_(μ_4_-S)]^n+^ (*n* = 2, **2**; *n* = 3, **3**) clusters, supported by a macrocyclic {py_2_NHC_4_} ligand (py = pyridine, NHC = *N*-heterocyclic carbene), in both their 0-hole (**2**) and 1-hole (**3**) states, thus mimicking the two active states of the Cu_Z_* site during enzymatic N_2_O reduction. Structural and electronic properties of these {Cu_4_(μ_4_-S)} clusters are elucidated by employing multiple methods, including X-ray diffraction (XRD), nuclear magnetic resonance (NMR), UV/vis, electron paramagnetic resonance (EPR), Cu/S K-edge X-ray emission spectroscopy (XES), and Cu K-edge X-ray absorption spectroscopy (XAS) in combination with time-dependent density functional theory (TD-DFT) calculations. A significant geometry change of the {Cu_4_(μ_4_-S)} core occurs upon oxidation from **2** (τ_4_(S) = 0.46, seesaw) to **3** (τ_4_(S) = 0.03, square planar), which has not been observed so far for the biological Cu_Z_(*) site and is unprecedented for known model complexes. The single electron of the 1-hole species **3** is predominantly delocalized over two opposite Cu ions via the central S atom, mediated by a π/π superexchange pathway. Cu K-edge XAS and Cu/S K-edge XES corroborate a mixed Cu/S-based oxidation event in which the lowest unoccupied molecular orbital (LUMO) has a significant S-character. Furthermore, preliminary reactivity studies evidence a nucleophilic character of the central μ_4_-S in the fully reduced 0-hole state.

## Introduction

**Figure 1:**
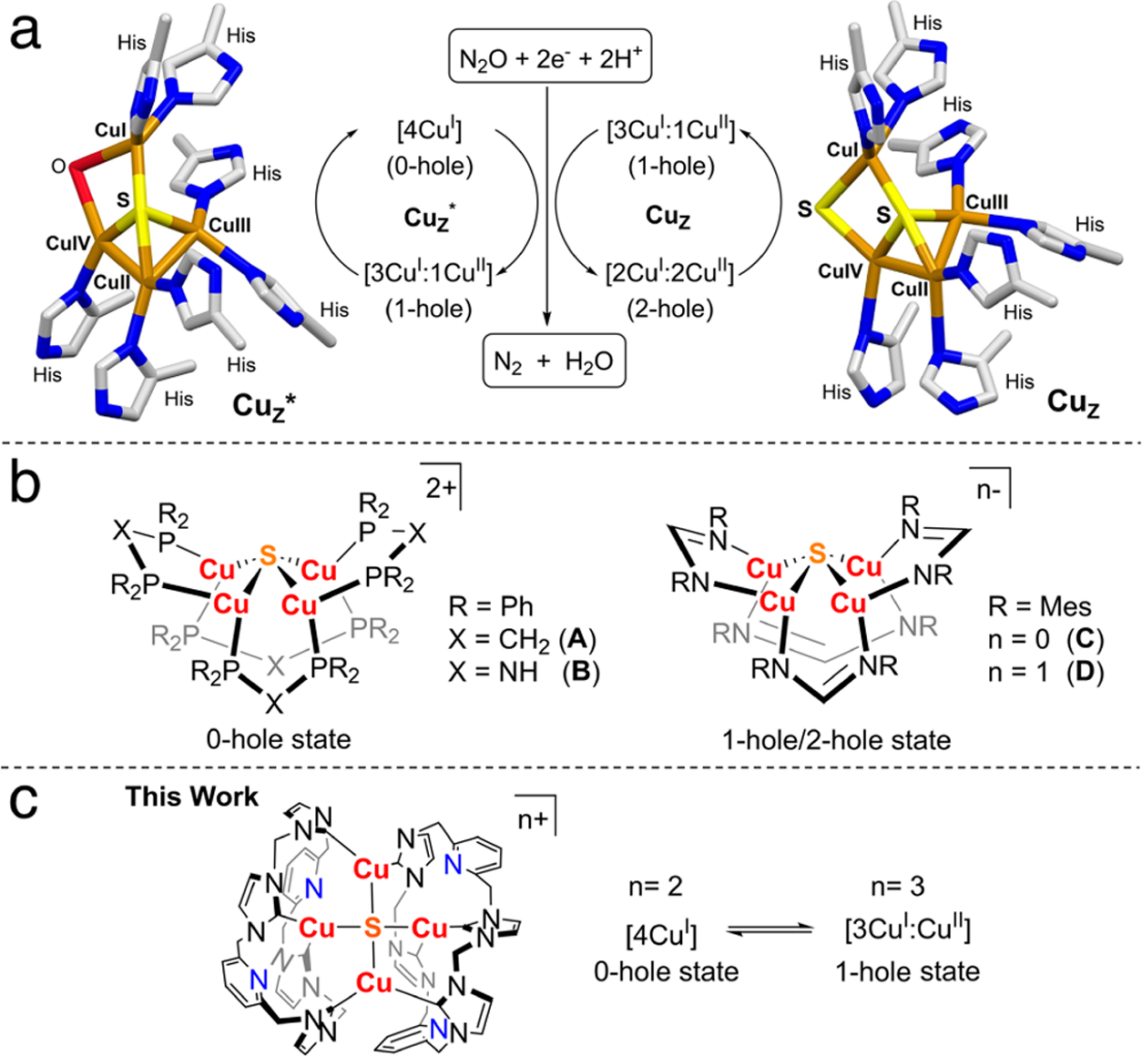
(a) Solid structures of Cu_Z_* (PDB: 1fwx)^13^ and Cu_Z_ (PDB: 3sbp)^15^ derived from N_2_OR and their active states in the N_2_O reduction. (b) Literature reported model complexes. (c) {Cu_4_(μ_4_-S)} model complexes reported in this work.

Nitrous oxide (N_2_O) is an environmentally problematic compound due to its dual roles as a potent greenhouse gas with a global warming potential 300 times higher than that of CO_2_ and as an ozone layer depletion agent with an impact comparable to that of notorious chlorofluorocarbons.^1,2^ Although the 2e^–^/2H^+^ reduction of N_2_O to N_2_ and H_2_O is thermodynamically favorable (Δ*G*^o^ = −81 kcal/mol), a catalyst is required due to the high activation barrier (Δ*G*^‡^= +59 kcal/mol).^3^ In nature, N_2_O reduction is efficiently catalyzed by the metalloenzyme nitrous oxide reductase (N_2_OR) during the final step of bacterial denitrification, where N_2_O acts as an electron acceptor for anaerobic respiration.^4−7^ Two copper sites are present in N_2_OR, the binuclear Cu_A_ site responsible for electron transfer (ET) and the Cu_Z_* or Cu_Z_ site, depending on the isolation conditions, where the N_2_O reduction takes place.^8−12^ The Cu_Z_*/Cu_Z_ catalytic site of N_2_OR features a unique μ_4_-sulfido-capped tetranuclear copper core ligated by seven histidine residues and with either a solvent-derived ligand (for Cu_Z_*)^13,14^ or a μ_2_-S^2–^ (for Cu_Z_)^15^ on the CuI–CuIV edge (Figure 1a). The resting state of Cu_Z_* is the [3Cu^I^:Cu^II^] (1-hole, *S* = 1/2) state, which is reduced to the active [4Cu^I^] (0-hole, *S* = 0) state en route to the catalytic N_2_O reduction cycle where this 0-hole species interconverts with an active 1-hole intermediate (Cu_Z_°); the latter has been spectroscopically observed, along with the electron transfer (Figure 1a).^16−23^ In contrast, the Cu_Z_ site has a [2Cu^I^:2Cu^II^] (2-hole, *S* = 1) resting state and converts to a 1-hole state that shows limited N_2_O reduction activity (Figure 1a).^21^

The {Cu_4_(μ_4_-S)} cores of both resting Cu_Z_* and Cu_Z_ sites adopt a seesaw geometry with a large CuI–S–CuII angle of ∼160° (Figure 1a),^13,15^ which allows for pronounced σ interactions between the Cu(I/II) d_*x*^2^−*y*^2^_ and the S 3p_*y*_ orbitals, thus leading to a CuI–S–CuII σ/σ superexchange pathway for electron delocalization in the 1-hole Cu_Z_* form.^17,18^ Electron paramagnetic resonance (EPR) and magnetic circular dichroism (MCD) spectroscopies evidenced that the spin density of 1-hole Cu_Z_* is delocalized unevenly over the two Cu ions (∼5:2),^17,18^ whereas the spin population is evenly localized on two and three Cu ions for the 1-hole Cu_Z_° and Cu_Z_, respectively,^23,24^ indicating significant structural differences between these states. Spectroscopic methods in combination with theoretical calculations have shown that the additional coordination site at the CuI–CuIV edge may be vacant (for 0-hole Cu_Z_*) or may be occupied by a μ_2_–OH (for resting Cu_Z_*),^20,25^ terminal CuIV–OH (for Cu_Z_°),^23^ μ_2_-S^2–^ (for Cu_Z_),^15^ and μ_2_-SH^–^ (for 1-hole Cu_Z_).^24^ Despite the apparent distinctions in the edging ligands, the {Cu_4_(μ_4_-S)} core structures were so far assumed to be largely unchanged, mostly on the basis of computational models that used truncated structures derived from the parental resting species, which also satisfies the requirement for a minimal reorganization energy during ET processes.^23,24^ Nevertheless, due to the lack of crystallographic data of N_2_ORs where the Cu_Z_*/Cu_Z_ site is in a nonresting state, the structural properties of Cu_Z_* (1-hole), Cu_Z_°, and Cu_Z_ (1-hole), especially their {Cu_4_(μ_4_-S)} core structures, are yet to be investigated. Gaining experimental information about geometric and electronic structure variations during redox interconversion of the {Cu_4_(μ_4_-S)} unit would thus be valuable and might contribute to the understanding of the catalytic mechanism of N_2_O reduction.

The unique {Cu_4_(μ_4_-S)} core structure and the rich redox chemistry of the Cu_Z_*/Cu_Z_ site have stimulated many efforts to synthesize model complexes with such a motif, not only as an alternative way to study the short-lived biological intermediates but also to exploit the use of such metal clusters in, e.g., photochemistry and catalysis.^26−28^ However, building a tetranuclear copper core bearing a single S-cap has proven extremely challenging due to the propensity to form copper clusters with multiple S atoms.^29−32^ So far, only very few model complexes containing the {Cu_4_(μ_4_-S)} motif have been reported, and their supporting ligands are limited to bidentate phosphine ligands (PXP, where X = CH_2_ or NH) or amidinate ligands (NCHN^–^). The first synthetic example of a {Cu_4_(μ_4_-S)} cluster was reported by Yam et al. in 1993 before the structure of N_2_OR was elucidated; hence, it was not discussed in the context of the biological site.^33,34^ Its {Cu_4_(μ_4_-S)} core, which was isolated solely in the [4Cu^I^] state, was supported by a diphosphine ligand (dppm) and featured a square pyramidal geometry (Figure 1b, **A**). After Yam’s work, some di- and tricopper clusters bridged by a single S atom were reported,^35−37^ but the second example of a synthetic {Cu_4_(μ_4_-S)} cluster appeared only in 2014.^38^ Modifying the dppm ligand, Mankad et al. isolated a {Cu_4_(μ_4_-S)} cluster with NH-bridged bidentate phosphorus ligands (dppa; Figure 1b, **B**), yet again only in the [4Cu^I^] state. Interestingly, this complex showed stoichiometric N_2_O reduction reactivity under very specific reaction conditions, which was proposed to be supported by the secondary coordination sphere N–H groups that could serve as hydrogen bond donors.^39^ A seminal breakthrough was made by the same group when the neutral diphosphine ligands were replaced by anionic amidinate ligands (NCHN^–^), and a redox pair of {Cu_4_(μ_4_-S)} clusters in the [2Cu^I^:2Cu^II^] (2-hole; Figure 1b, **C**)^40^ and [3Cu^I^:Cu^II^] (1-hole, Figure 1b, **D**)^41^ states was obtained. The {Cu_4_(μ_4_-S)} cores in both states adopt square pyramidal geometries with rather bent Cu–S–Cu (opposite Cu ions) angles of up to 126°. EPR spectroscopy showed that the unpaired electron of the 1-hole species is delocalized evenly over the four Cu ions. At low temperature (−78 °C), N_2_O can be stoichiometrically reduced by the 1-hole species to N_2_ and O^2–^ along with the formation of the 2-hole species.^41,42^

In spite of the significant achievements made by the Yam and Mankad groups, the modeling chemistry for the unique Cu_Z_ site is still very limited, with an obvious scarcity of ligand scaffolds that can stabilize {Cu_4_(μ_4_-S)} clusters in different states. In particular, a system that allows for the isolation and investigation of a pair of {Cu_4_(μ_4_-S)} complexes in the biologically relevant [3Cu^I^:Cu^II^] (1-hole, *S* = 1/2) and [4Cu^I^] (0-hole, *S* = 0) states is still lacking. *N*-Heterocycle carbenes (NHCs) are increasingly employed as alternative ligands and as surrogates of histidine residues in bioinspired model chemistry, facilitating the stabilization of reactive intermediates and unusual oxidation states.^43−49^ In our recent work, we found that the macrocycles {py_2_NHC_2_} (L’, py = pyridine) and {py_2_NHC_4_} (L), featuring combinations of pyridine and NHC donors, show great flexibility and can support mononuclear complexes [L’Cu]^1/2/3+^ in various Cu oxidation states (+I, +II, and +III)^50,51^ as well as binuclear complexes [LCu_2_]^2/3+^ in the Cu^I^Cu^I^ and mixed-valent Cu^1.5^Cu^1.5^ states,^52^ respectively. The mixed-valent complex [LCu^1.5^Cu^1.5^]^3+^ features a large spin delocalization energy and fast electron self-exchange rate, which structurally and functionally mimics the Cu_A_ site. In the present study, novel {Cu_4_(μ_4_-S)} clusters scaffolded by the macrocycle L were isolated and fully characterized in both the 0-hole and 1-hole states, i.e., in the active states of the Cu_Z_* site during the N_2_O reduction process (Figure 1a,c). These are distinct from the few synthetic {Cu_4_(μ_4_-S)} clusters reported so far, and a significant redox-induced structural change in the {Cu_4_(μ_4_-S)} core has now been evidenced by X-ray diffraction (XRD) analyses. The corresponding electronic structure changes have been analyzed by a range of spectroscopic techniques, including UV–vis, EPR, Cu K-edge X-ray absorption spectroscopy (XAS), and Cu/S X-ray emission spectroscopy (XES), and the experimental results were correlated with DFT calculations.

## Results and Discussion

### Cu_4_(μ_4_-S) Cluster in 0-Hole State

**Scheme 1:**
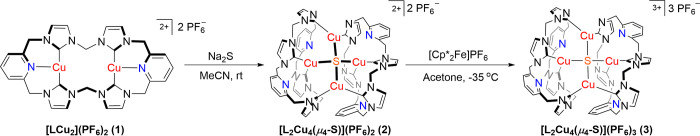
Synthesis of Cu_4_(μ_4_-S) Clusters in 0-Hole and 1-Hole States

**Figure 2:**
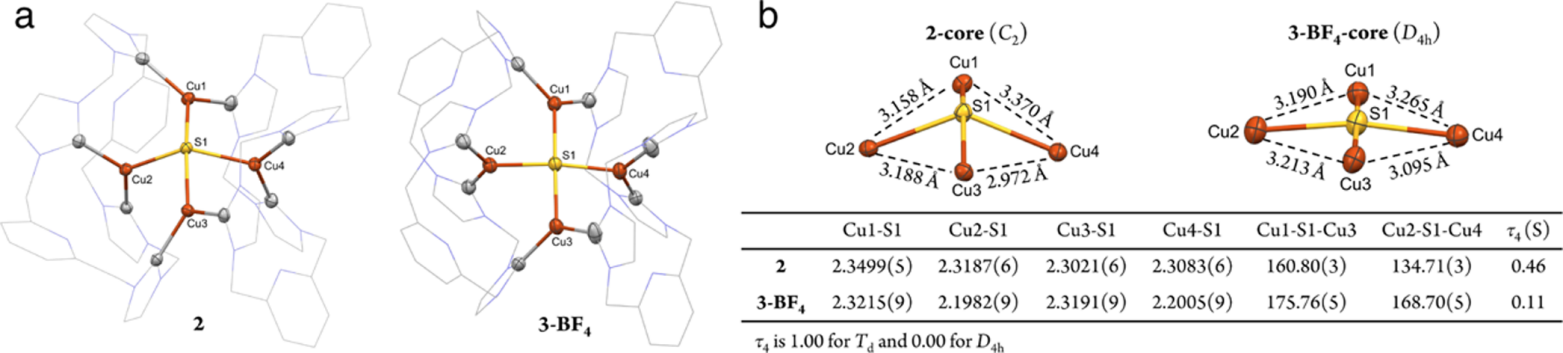
(a) Solid-state structures of the cations of **2** and **3-BF**_**4**_ with 50% probability ellipsoids. Hydrogen atoms, anions, and lattice solvents have been omitted for clarity. (b) Core structures of **2** and **3-BF**_**4**_ and selected bond lengths (Å) and angles (^o^).

Initially, we were aiming at the synthesis of a μ_2_-S-bridged neutral dicopper(I,I) species [LCu_2_(μ_2_-S)] that could mimic a fragment of the core structure of the Cu_Z_*/Cu_Z_ site. However, treating the dicopper(I,I) complex [LCu_2_](PF_6_)_2_ (**1**) with 1 equiv of sodium sulfide (Na_2_S) in acetonitrile (MeCN) gave, after workup and slow diffusion of diethyl ether (Et_2_O) into the MeCN solution, the μ_4_-S-capped tetranuclear copper complex, [L_2_Cu_4_(μ_4_-S)](PF_6_)_2_ (**2**) as yellow crystals (Scheme 1). X-ray diffraction shows that **2** has a {Cu_4_(μ_4_-S)} core supported by two macrocyclic ligands L (Figure 2a). All Cu ions are ligated by two NHC donors but with a rearranged coordination mode of L compared to the precursor complex **1**. Two Cu ions (Cu2 and Cu4) in opposite positions are coordinated by NHCs from the same ligand (L) but different {pyNHC} cavities, while the other two opposite Cu ions (Cu1 and Cu3) are coordinated by NHCs from different ligands L, thus rendering two pairs of Cu ions in distinct coordination environment (Cu2 and Cu4 vs Cu1 and Cu3). In contrast, in all other Cu_Z_* model complexes reported so far, neighboring Cu ions of the {Cu_4_(μ_4_-S)} core are spanned by bidentate phosphine or amidinate ligands in the same coordination mode (Figure 1b, **A**–**D**).^33,38,40,41^ The Cu–S bond distances in **2** are found in the narrow range 2.3021(6)–2.3499(5) Å and are similar to the Cu–S distances in the Cu_Z_*/Cu_Z_ sites of N_2_OR.^13,15^ The projection of the Cu_4_ base is an irregular quadrilateral, with Cu···Cu distances varying from 2.972(1) to 3.370(1) Å (Figure 2b). The Cu1–(μ_4_-S1)–Cu3 angle of 160.80(3)^o^ is comparable to that in the Cu_Z_*/Cu_Z_ sites (∼160°), while the Cu2–(μ_4_-S1)–Cu4 angle of 134.71(3)^o^ is larger than that in the biological systems (∼90 ^o^). The τ_4_ value is 0.46 for the μ_4_-sulfide ligand in **2**, indicating a seesaw shape of the {Cu_4_(μ_4_-S)} core (τ_4_ is 1.0 for tetrahedral and 0.0 for square planar),^53^ which is smaller than τ_4_(μ_4_-S) in the Cu_Z_*/Cu_Z_ sites (0.74/0.71).^13,15^ Notably, this τ_4_(μ_4_-S) value is the smallest among the reported Cu_Z_*/Cu_Z_ model complexes (0.59–0.90), in which the {Cu_4_(μ_4_-S)} core is best described as a square pyramid with more bent Cu−μ_4_-S–Cu angles (opposite Cu ions, 101.3–145.6°). Interestingly, the molecular structure of the cation of **2** in the solid state has an approximate *C*_2_ local symmetry. However, ^1^H NMR spectra in different solvents (*d*_3_-MeCN, *d*_6_-acetone, and *d*_6_-DMSO) show only one set of signals in the temperature range studied (238–358 K; Figures S4–S6, S16, and S17) with equivalent protons of both halves of the macrocyclic ligand (L), implying a higher level of symmetry in solution. This might be caused by the fast conformational inversion of the {Cu_4_(μ_4_-S)} core as well as the ligand macrocycle, leading to an averaged overall *C*_2v_ symmetry on the NMR time scale.

At room temperature (rt), **2** is stable both in solid state and in common solvents (e.g., MeCN, acetone, DMF, and DMSO) under an inert atmosphere. In MeCN solution under aerobic conditions, however, it decomposes slowly to give precursor complex **1**, which likely proceeds through the oxidation of **2** by O_2_ and subsequent decomposition of the oxidized species (*vide infra*). The reformation of **1** shows that the Cu–C^NHC^ bonds are labile and that the rearrangement of L, interconverting the binding modes seen in **2** and **1**, is reversible. The identity of **2** was further confirmed by combustion analysis and by electrospray ionization mass spectrometry (ESI-MS); the latter shows a dominant peak at *m*/*z* = 645.1 amu (in MeCN) with an isotope pattern that is characteristic of the dication [L_2_Cu_4_(μ_4_-S)]^2+^ (Figure S19), indicating that the {Cu_4_(μ_4_-S)} core structure of **2** is retained in solution.

**Figure 3:**
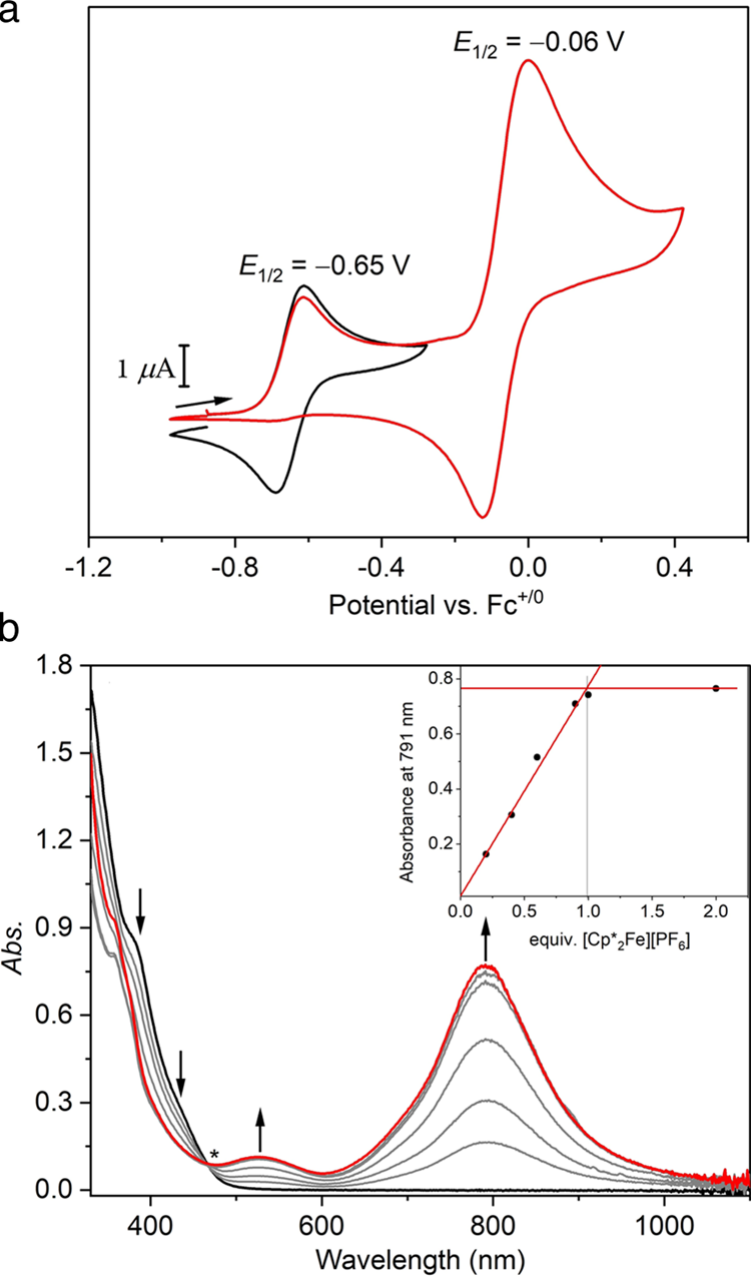
(a) Cyclic voltammogram of **2** in MeCN (0.1 M [^*n*^Bu_4_N]PF_6_, 100 mV/s) at room temperature with different scan ranges (black: – 0.98 to −0.28 V; red: −0.98 to +0.42 V). (b) UV–vis titration of **2** (black) with [Cp*_2_Fe]PF_6_ at −80 °C in acetone. Changes in the spectrum are indicated by black arrows; The isosbestic point is marked with an asterisk. The inset shows the increase of the absorption at 790 nm depending on the equivalents of [Cp*_2_Fe]PF_6_ added.

To assess if the 1-hole state of this {Cu_4_(μ_4_-S)} cluster can be accessed, which would emulate both active states of the Cu_Z_* site that are passed through in the catalytic N_2_O reduction cycle, the redox properties of **2** were examined electrochemically. Cyclic voltammetry (CV) of **2** in MeCN (100 mV/s) at rt reveals one reversible redox event at a potential of −0.65 V (*E*_1/2_, vs Fc^+/0^; Figures 3a and S25), assigned to the [L_2_Cu_4_(μ_4_-S)]^3+/2+^ redox couple when a small potential range from −0.98 to −0.28 V (vs Fc^+/0^) was scanned. This redox potential is significantly cathodically shifted compared to the 0-hole model complexes [(μ_2_-dppm)_4_Cu_4_(μ_4_-S)](PF_6_)_2_ and [(μ_2_-dppa)_4_Cu_4_(μ_4_-S)](PF_6_)_2_ (Figure 1b, **A** and **B**; *E*_pa_ = +0.26 and −0.05 V, vs Fc^+/0^, respectively) for which only irreversible oxidation events were observed.^27,28,38,54^ This difference can likely be attributed to the strong σ-donating ability of the NHC ligands; at the same time, the NHC-based scaffold L is capable of stabilizing the {Cu_4_(μ_4_-S)} core in both relevant oxidation states. However, the redox event at *E*_1/2_ = −0.65 V becomes irreversible when the scan range is extended anodically to +0.42 V (vs Fc^+/0^), and a quasi-reversible redox event at *E*_1/2_ = −0.06 V appears (Figure 3a) that is strikingly similar to the redox couple **1**^**ox**^/**1** (*E*_1/2_ = −0.09 V).^52^ This can be attributed to the rapid decomposition of the species resulting after 2-fold oxidation of **2**, giving two equiv of **1**. In line with this interpretation, the current of the redox event at *E*_1/2_ = −0.06 V is approximately twice the current of the initial oxidation of **2** at −0.65 V.

While the oxidized species (1-hole state) is metastable at rt, UV–vis titration experiments showed that it is relatively stable at low temperatures. The UV–vis spectrum of complex **2** (Figure 3b) in acetone at −80 °C displays a prominent shoulder band at 380 nm with a discernible shoulder at 430 nm. Upon titration with [Cp*_2_Fe]PF_6_ (Cp* = pentamethylcyclopentadienyl, *E*^o^’ = −0.59 V vs Fc^+/0^)^55^ at −80 °C, both absorptions of **2** vanish and an intense broad band at λ_max_ = 790 nm as well as a band with lower intensity at λ_max_ = 530 nm emerge, reaching a plateau in intensity after the addition of 1 equiv of oxidant (Figure 3b). The characteristic low-energy bands thus indicate the formation of the one-electron-oxidized species (1-hole state), [L_2_Cu_4_(μ_4_-S)](PF_6_)_3_ (**3**). The observation of an isosbestic point at 470 nm indicates a clean conversion of **2** to **3** without intermediate step(s) (Figure 3b). The interconversion between **2** and **3** is fully reversible, as evidenced by the reduction of **3** with 1 equiv of Cp_2_Co (Cp = cyclopentadienyl, *E*^o^’ = −1.33 V vs Fc^+/0^)^55^ and subsequent reoxidation with 1 equiv of [Cp*_2_Fe]PF_6_ (Figure S28). In situ-formed **3** is stable at −35 °C for several hours without noticeable decay according to UV–vis spectroscopy, suggesting that the isolation of **3** should be feasible at such low temperature.

### Cu_4_(μ_4_-S) Cluster in 1-Hole State

Bulk oxidation of **2** with [Cp*_2_Fe]PF_6_ was carried out at −35 °C in acetone (Scheme 1). After workup and crystallization, purple crystals of **3** were obtained; their quality was rather low but permitted the atom connectivity of the {Cu_4_(μ_4_-S)} core to be established by X-ray diffraction analysis (Figure S2). The cation of **3** is structurally similar to the cation of **2** consisting of a {Cu_4_(μ_4_-S)} core, yet in an unprecedented square planar geometry where the μ_4_-S resides in the center of the square with a τ_4_(S) value of 0.03. This is in remarkable contrast to **2** and all of the other model complexes (Figure 1b, **A**–**D**) as well as Cu_Z_*/Cu_Z_ sites where the {Cu_4_(μ_4_-S)} cores have either seesaw shapes or square pyramidal shapes. To improve the crystallization behavior, counteranion exchange of **3** with NaBF_4_ was subsequently performed, which resulted in high-quality purple crystals of **3-BF**_**4**_.

The structure of the cation of **3-BF**_**4**_ (Figure 2a) features a similar square planar {Cu_4_(μ_4_-S)} core as that in **3** with a slightly larger τ_4_(S) value of 0.11, which might originate from the collective packing effects of anions (PF_6_^–^ vs BF_4_^–^) and solvents (acetone vs MeCN). Upon oxidation to the 1-hole state, the Cu_4_ base in **3-BF**_**4**_ is less asymmetric than in **2**, with Cu···Cu distances in a narrower range (3.095(1)–3.265(1) Å; Figure 2b). The large Cu···Cu separations >3 Å, however, exclude any direct Cu–Cu interaction. The four Cu–S bonds, which have comparable lengths in **2**, become pairwise unequal in **3-BF**_**4**_ with two long Cu–S bonds (Cu1–S1, 2.3215(9) Å and Cu3–S1, 2.3191(9) Å) and two short Cu–S bonds (Cu2–S1, 2.1982(9) Å and Cu4–S1, 2.2005(9) Å). Careful inspection of the structure reveals that each Cu ion adopts a trigonal planar geometry; however, the {C_2_^NHC^CuS} planes centered on Cu2 and Cu4 are approximately perpendicular to the Cu_4_ base plane with a dihedral angle of 86.3 and 89.0°, respectively, while the {C_2_^NHC^CuS} planes centered on Cu1 and Cu3 are bisected by the Cu_4_ base plane with dihedral angles of 45.3 and 40.8°, respectively. Such a spatial configuration might affect the interaction of the Cu 3d orbitals with the μ_4_-S 3p orbitals, resulting in the inequality of Cu–S bond lengths in **3-BF**_**4**_. Notably, the two short Cu–S bonds (at Cu2 and Cu4, both ligated by NHC donors from the same ligand L) are significantly contracted compared to the corresponding Cu–S bonds in **2**, suggesting that the two opposite metal ions Cu2 and Cu4 are predominantly involved in the oxidation process (*vide infra*).

**Figure 4:**
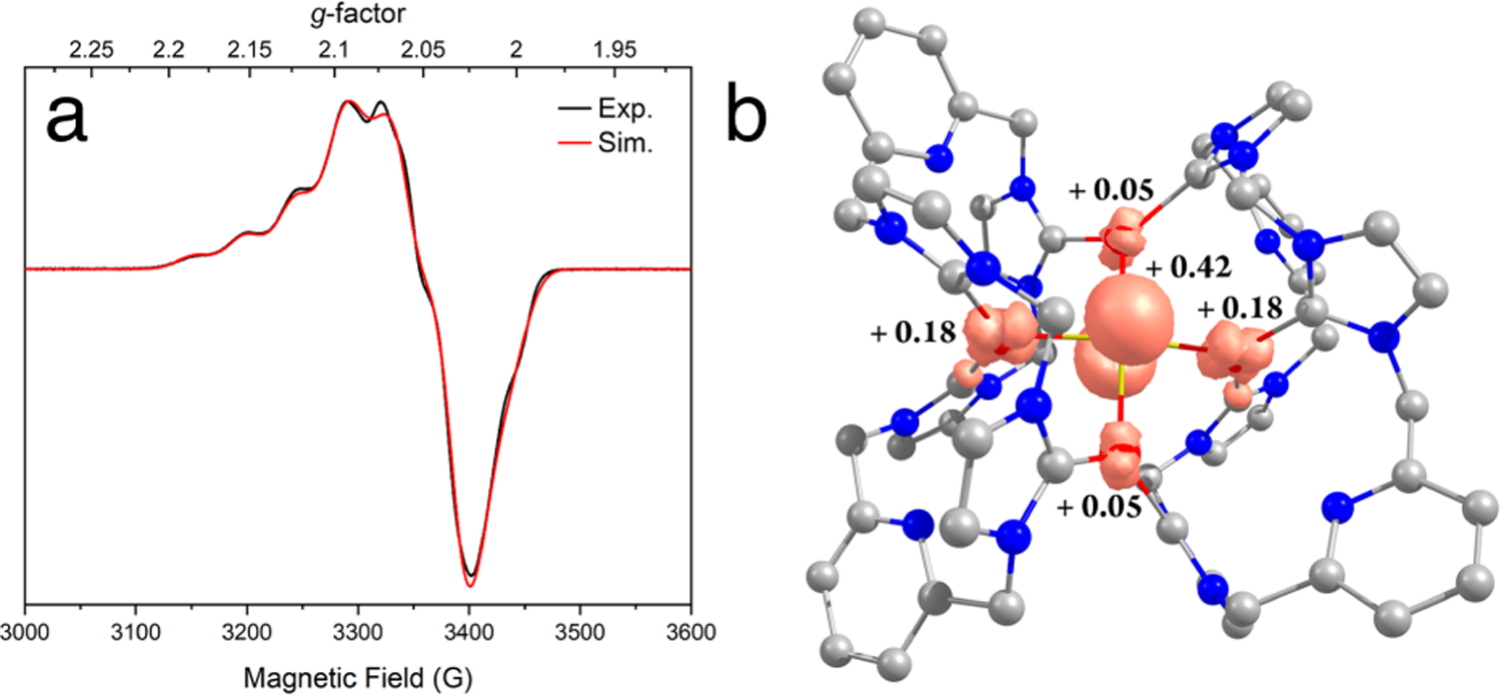
(a) CW X-band (9.63 GHz) EPR spectrum of **3** collected at 50 K (black) and a simulation (red) with the following parameters: ***g*** = [2.092, 2.064, 2.029], ***A***(Cu) = [128, 44, 10] MHz for two equal Cu ions, line width (full width at half-maximum, Gaussian line shape) = 3.4 mT with additional broadening along *g*_1_ of 1.4 mT. (b) Loewdin spin density population of **3** (isodensity value 0.08 au). Color code: C (gray), N (blue), S (yellow), and Cu (red).

The ^1^H NMR spectrum of **3** at 263 K displays broad singlets in the range of 0–18 ppm due to its paramagnetic nature (Figure S14). The *S* = 1/2 ground state of 1-hole **3** was confirmed by SQUID magnetometry where a constant χ_M_T value of 0.40 cm^–3^ mol^–1^ K (corresponding to μ_eff_ = 1.79 μB) over the entire temperature range from 2 to 300 K was observed and best-fitted as an *S* = 1/2 system with *g*_iso_ = 2.07 (Figure S22). The continuous-wave X-band (9.63 GHz) EPR spectrum of **3** in acetone recorded at 50 K is pseudoaxial with a hyperfine pattern on the low field side attributed to the ^63/65^Cu (*I* = 3/2) nuclei, with relatively small splittings and an intensity pattern that suggests the unpaired electron is delocalized over more than one Cu ion (Figure 4a). The spectrum features a seven-line hyperfine splitting (2*nI*+1; where *n* is the number of equivalent nuclei) and intensity pattern resulting from two equivalent copper centers.^52^ To further resolve the EPR parameters, a two-pulse echo-detected Q-band (34.0 GHz) EPR spectrum of **3** in acetone was collected at 15 K. This higher-frequency spectrum resolved the rhombic splitting of the g-tensor, particularly the narrow *g*_3_ feature (Figure S23). The overall line width of the Q-band spectrum is larger than the X-band spectrum, a common occurrence for Cu centers measured at higher microwave frequencies, resulting in only a broad *g*_1_ feature without any well-resolved copper hyperfine coupling.^56^ Both the X- and Q-band spectra are best simulated with a rhombic *g*-tensor ***g*** = [2.092, 2.064, 2.029] and two equivalent Cu hyperfine tensors ***A***(Cu) = [128, 44, 10] MHz. The *g*_iso_ of 2.06 also agrees well with the value derived from the magnetic susceptibility measurements. Most importantly, the model and simulation with two equivalent Cu ions reproduce best both the position and breadth of the *g*_1_(*A*_1_) features. Attempts to reproduce both the X-and Q-band EPR spectra by simulation with only a single Cu hyperfine interaction were unsuccessful and unable to reproduce the multifrequency EPR data (Figure S24). Therefore, the multifrequency EPR analysis evidences that the unpaired electron in 1-hole **3** is delocalized over two equivalent Cu ions.

It is worth noting that spin delocalization equally over two oppositely positioned Cu ions has also been observed for the biological 1-hole Cu_Z_° site, which is the active intermediate species during the N_2_O catalytic cycle.^23^ In addition, the maximum Cu hyperfine coupling in **3** (128 MHz) is comparable to that in Cu_Z_° (118 MHz), indicating that the spin population on the Cu ions in these two species is similar (Table 1). In contrast, the EPR signatures for other 1-hole {Cu_4_(μ_4_-S)} sites or model complex **D** (Table 1) show a different spin delocalization, either unequally over two Cu ions (with a ratio of ∼5:2 for the Cu_Z_* site) or equally over three (for the reduced Cu_Z_ site) or four Cu ions (for model complex **D**).

**Table 1:** EPR Spectroscopic Signatures of {Cu_4_(μ_4_-S)} Clusters in the 1-Hole State

	**3**	Cu_Z_*^a^	Cu_Z_°^b^	Cu_Z_^c^	**D**^d^
*g*_⊥_	2.029	2.043	2.050	2.042	2.090
	2.064				
*g*_||_	2.092	2.160	2.177	2.152	2.043
A_⊥_^e^	10, 44	75, 60	126	60	100
*A*_||_^e^	128	182, 69	126	168	15
spin	2 Cu	2 Cu	2 Cu	3 Cu	4 Cu
del.^f^	(even)	(5:2)	(even)	(even)	(even)

a Ref (17).

b Ref (23).

c Ref (24).

d Ref (41).

e In MHz.

f Number of Cu ions the spin is delocalized over.

The electronic structure of these {Cu_4_(μ_4_-S)} clusters (**2** and **3-BF**_**4**_) was further investigated by density functional theory (DFT) calculations at the B3LYP/def2-TZVP level. The seesaw and square planar {Cu_4_(μ_4_-S)} core structures of the cations of **2** and **3-BF**_**4**_, respectively, as well as relevant bond lengths/angles, were well reproduced in the optimized structures (τ_4_(S) = 0.44 and 0.10; Table S3). The singly occupied molecular orbital (SOMO, unrestricted natural orbital (UNO) 328) of **3-BF**_**4**_ is primarily composed of Cu 3d_*yz*_ (Cu2 and Cu4, 17% each)/3d_*xz*_ (Cu1 and Cu3, 6% each) orbitals and the μ_4_-S 3p_*z*_ (39%) orbital featuring π-antibonding interactions (*z*-axis is perpendicular to the Cu_4_S plane; Figure S29). Such π* interactions in the ground state of **3-BF**_**4**_ contrast the situation in the 1-hole Cu_Z_* site^17,18^ and model complex **D**(41,42) where the electron delocalization is mediated by σ* interactions between the Cu and S atoms. This can be attributed to the distinct macrocyclic ligand scaffold of L that induces a configuration where the {C_2_^NHC^CuS} planes (centered on Cu2 and Cu4) are almost perpendicular to the Cu_4_ base plane (*vide supra*). Such a configuration, combined with the large Cu–(μ_4_-S)–Cu angles, enables the effective π/π overlap of Cu 3d_*yz*_/3d_*xz*_ orbitals and the μ_4_-S 3p_*z*_ orbital in **2**/**3-BF**_**4**_ (Figure S29). Removing one electron from the π* HOMO of **2** upon oxidation strengthens the Cu–S bonds, especially the Cu2–S1 and Cu4–S1 bonds, which accordingly leads to the planarization of the {Cu_4_(μ_4_-S)} core structure. The Loewdin spin density was found to be delocalized over the four Cu ions via these π* interactions between the Cu and S atoms, constituting an excellent superexchange pathway (Figure 4b). The unpaired electron spin is predominantly located on the μ_4_-S center (42%) and moderately located on the Cu2 and Cu4 ions (each 18%), while the Cu1 and Cu3 ions feature only minor spin populations (each 5%) (Figure 4b). These computational findings are consistent with the EPR results where hyperfine coupling to two Cu ions was observed (here identified as Cu2 and Cu4). Notably, the spin population on the central μ_4_-S in **3-BF**_**4**_ (42%) is obviously larger than that in the Cu_Z_* site (14%)^17^ and model complex **D** (32%),^41^ indicating a higher extent of S participation in the redox process. The large spin delocalization on the μ_4_-S and high covalency of the Cu–S and Cu−NHC bonds might also account for the rather small anisotropy of the EPR spectrum that adopts a small *g*_1_ value.^41,57^

To obtain further insight into the electronic structure variations of the [L_2_Cu_4_(μ_4_-S)]^3+/2+^ redox couple, Cu K-edge X-ray absorption (XAS), and Cu and S valence to core (VtC) X-ray emission spectra (XES) of **2** and **3** were measured. These data, together with the corresponding calculated spectra, are shown in Figures S34–S36. The Cu K-edge XAS (Figure S34) clearly evidences an increase in the rising edge position of **3** as compared to that of **2** (by ∼1 eV) consistent with a significant metal-based contribution to the oxidation event in **3**. The Cu and S VtC XES probe the transitions from the filled valence levels to the 1s core holes on the Cu and S, respectively;^58,59^ hence, taken together, the relative contributions of copper and sulfur to the filled bonding levels can be experimentally assessed. The Cu VtC XES (Figure S35) shows very similar spectra for **2** and **3**, highlighting that the Cu K-edge XAS, which primarily probes the lowest unoccupied molecular orbital (LUMO), is a more sensitive electronic structure probe in the present case. It is of interest to note that the S VtC XES (Figure S36) shows clear changes between **2** and **3**, suggesting a modulation of the sulfur involvement in bonding, which may be attributed to either a substantial ligand-based oxidation or increased sulfur covalency. Time-dependent (TD)-density functional theory (DFT) and one-electron DFT calculations were utilized to calculate the XAS and XES spectra, respectively. The general trends in the data are well-reproduced by the calculations, supporting that the DFT electronic structures of **2** and **3** are reasonable descriptions of the electronic ground states (Figures S34–S36). Complex **2** is best described as a fully reduced cluster, while **3** has a hole character with a LUMO that is ∼50% copper-based and ∼50% ligand-based, reflecting the high covalency of the cluster (Table S8 and Figure S37).

**Figure 5:**
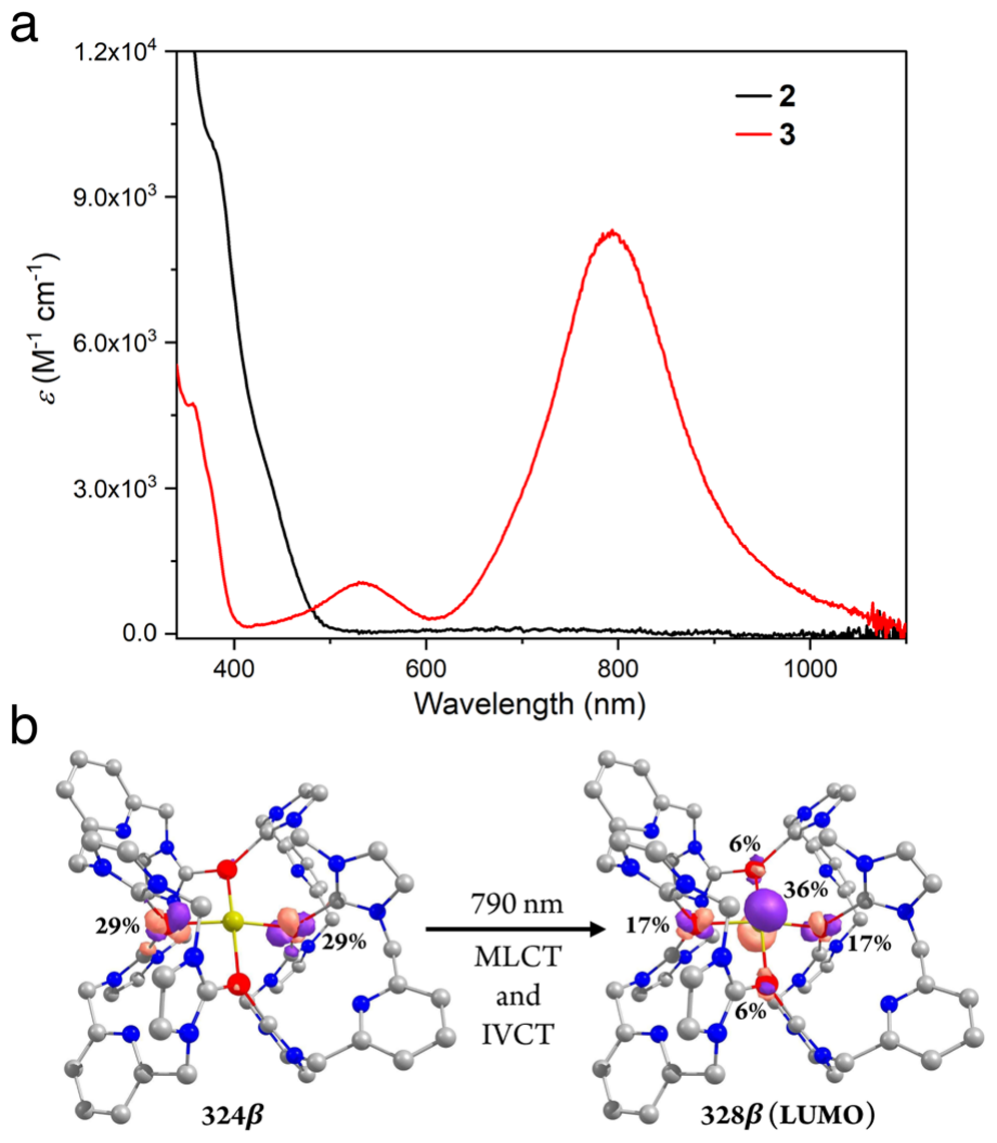
(a) UV–vis spectra of **2** (black) and **3** (red) in acetone at −35 °C. (b) TD-DFT calculated transition orbitals for 790 nm excitation of **3** (isodensity value 0.08 au). Color code: C (gray), N (blue), S (yellow), and Cu (red).

The UV–vis spectra of pure samples of complexes **2** and **3** (Figure 5a) in acetone at −35 °C are consistent with the spectra observed in the UV–vis titration experiments (Figure 3b). The shoulder bands for **2** at 380 nm (ε = 10000 M^–1^ cm^–1^) and 430 nm (ε = 3700 M^–1^ cm^–1^) are assigned to transitions from MOs of mainly Cu 3d and μ_4_-S 3p character-to-ligand (L)-based MOs according to TD-DFT calculations (Table S4 and Figures S30 and S31). The characteristic intense band at λ_max_ = 790 nm (ε = 8260 M^–1^ cm^–1^) for **3** is of particular interest. TD-DFT calculations predict an intense absorption band whose main contribution is β electron excitation from the 324β to the 328β (the spin-down LUMO) orbital (Figure 5b; see the Supporting Information (SI) for detailed information). The 324β orbital is predominately composed of 3d_*yz*_ orbitals from Cu2 and Cu4 ions (29%, each), while the composition of 328β is similar to the SOMO (*vide supra*) as they are essentially the same orbital in a spin-restricted formalism. Thus, the transition at λ_max_ = 790 nm corresponds to a mixture of Cu2/Cu4 to S charge transfer (MLCT) and metal–metal charge transfer from Cu2/Cu4 to Cu1/Cu3 (MMCT). Considering the large difference in the spin populations on Cu2/Cu4 and Cu1/Cu3, the latter transition can be seen as an intervalence charge transfer (IVCT). In contrast, absorption bands at 680 and 694 nm with lower intensity (<5000 M^–1^ cm^–1^) were observed for the 1-hole Cu_Z_* and Cu_Z_ sites, respectively, and have been attributed to S-to-Cu charge transfer (LMCT).^24^ The 1-hole model complex **D** shows an intense band at 566 nm (8600 M^–1^ cm^–1^), which was assigned to charge transfer from the four Cu ions to the S center (MLCT).^41^

Given that the fully reduced (0-hole) state and the 1-hole state are proposed as active states of Cu_Z_* in the catalytic cycle of N_2_O reduction, and the coordination mode of N_2_O at the catalytic site remains under debate, we performed preliminary reactivity studies to assess the reactivity profile of the new 0-hole cluster **2**. It does not react with N_2_O or triatomic anions such as linear N_3_^–^ or bent NO_2_^–^ (that are related to the geometries of N_2_O in its ground state or in a transition state proposed in biological N_2_O activation, respectively),^20^ and only anion exchange is observed with iodide (I^–^), a known inhibitor of N_2_OR,^60^ giving [L_2_Cu_4_(μ_4_-S)]I_2_ (**4**; see the SI for detailed information). The large steric hindrance around the metal ions enforced by the two macrocyclic ligands may account for the inertness of **2** toward these substrates. Though the four Cu ions are shielded by the macrocyclic ligands, however, an open cleft allowing for access to the central μ_4_-S is present on one side of the Cu_4_S cluster in the 0-hole state, which could make an attack on the S atom possible; this contrasts the situation in the planar 1-hole state Cu_4_S cluster, where this cleft is mostly closed (Figure S56). The large contribution of the μ_4_-S 3p_*z*_ orbital (39%), which is perpendicular to the Cu_4_S plane, to the HOMO of **2** was expected to impart a nucleophilic character to the S center. Indeed, **2** reacts rapidly with electrophilic [Me_3_O]BF_4_ via *S*-alkylation, giving the dicopper(I) complex **1** and its thiolato-bridged congener [LCu^I^_2_(μ_2_-SMe)]^+^ (**5**; see the SI for details). A distinct N_2_O activation mode where the S atom is involved as one binding site has been proposed.^42^ Attack at the S atom (3p_*z*_ orbital) in **2** is an indication of the potential ability of **2** to functionally mimic the Cu_Z_* site. Modification of the macrocyclic ligand to provide a larger ring, increased metal ion access, and more electron-rich Cu ions are undergoing.

## Summary and Conclusions

The present study demonstrates that a model of the unique {Cu_4_(μ_4_-S)} cluster found in the active Cu_Z_* or Cu_Z_ site of the metalloenzyme N_2_O reductase, which mediates the final step of bacterial denitrification, can be isolated by using a flexible hexadentate macrocyclic ligand system L that provides two binding pockets and NHC ligation for the Cu ions. This new {Cu_4_(μ_4_-S)} cluster is distinct from the very few reported Cu_Z_*/Cu_Z_ models that are based on bidentate {PXP} and {NCN} ligands. [L_2_Cu_4_(μ_4_-S)]^2+/3+^ could be isolated in the fully reduced 0-hole state ([4Cu^I^], *S* = 0) and in the singly oxidized 1-hole state (*S* = 1/2), viz. in both states that have been proposed for the relevant intermediates of Cu_Z_* in the catalytic N_2_O reduction cycle. Comprehensive structural and spectroscopic characterization of the [L_2_Cu_4_(μ_4_-S)]^2+/3+^ complexes has provided novel insights into how the {Cu_4_(μ_4_-S)} core structure of Cu_Z_* might adapt to the 1e^–^ oxidation state change, revealing a planarization upon oxidation that gives rise to pronounced π* interactions between Cu 3d_*yz*_/3d_*xz*_ orbitals and the μ_4_-S 3p_*z*_ orbital. The unpaired electron spin in the 1-hole state is almost equally located on the μ_4_-S center and on the Cu ions (in total), with metal contributions predominantly from two Cu ions located opposite each other in the {Cu_4_(μ_4_-S)} core. This is similar to what has been proposed for the active 1-hole intermediate (Cu_Z_°) of Cu_Z_*, and it is also reflected in a significant contraction of the corresponding Cu–S bonds upon oxidation of the present {Cu_4_(μ_4_-S)} model cluster from its 0-hole state to its 1-hole state. Cu K-edge XAS and Cu/S K-edge XES spectroscopies, combined with DFT calculations, corroborated a mixed metal- and sulfur-based hole character with a highly covalent Cu_4_S core of the oxidized species.

How can these findings be related to the enzyme active site? In previous computational models that predicted the {Cu_4_(μ_4_-S)} core structures of the Cu_Z_(*) site to be largely unchanged in both resting and active states, structural constraints imposed by the protein scaffold were considered by fixing the position of the distal nitrogen or the C_γ_ atoms of each histidine ligand in the DFT geometry optimizations.^20,23,24^ However, theoretical work also indicated that, if all constraints are removed, the original seesaw {Cu_4_(μ_4_-S)} cluster rearranges to a trigonal pyramidal geometry with three Cu and S atoms at its base and that the protein strain energies are higher for the 1-hole species.^20^ The latter is reminiscent of our 1-hole complex, which indeed undergoes a structural rearrangement from a seesaw to a square planar core geometry. Though the protein environment was suggested to be strained, the flipping of some histidine rings^61^ as well as a conformational switch of the active site relevant to its function has indeed been observed for these N_2_OR enzymes^62,63^ and has been suggested for their Cu_A_ site by theoretical calculations as well.^64^ Furthermore, a complex network of hydrogen bonds involving the protein environment has been suggested to determine the orientation of the histidine side chains.^65^ Therefore, a change in local pH or a shift in hydrogen bonding could lead to a change in the orientation of the histidine ligands, resulting in a corresponding change in the geometry of the {Cu_4_(μ_4_-S)} core. Based on this, geometric and electronic structure variations of the Cu_Z_(*) sites are conceivable when shuttling through different states during N_2_O reduction; the present pair of bioinspired {Cu_4_(μ_4_-S)} clusters **2**/**3** clearly illustrates possible variations in response to the 1e^–^ oxidation state change.

## Supporting Information Available

Experimental details and synthetic procedures, further spectroscopic and analytical data, crystallographic information, and details of DFT calculations (PDF)
