# Supplementary material for: Cu4S Cluster in “0-Hole” and “1-Hole” States: Geometric and Electronic Structure Variations for the Active CuZ* Site of N2O Reductase
Source: J Am Chem Soc. 2023 Aug 11;145(33):18477–86. doi: 10.1021/jacs.3c04893 (PMC10450684; doi:10.1021/jacs.3c04893)
Supplement: Supplementary file 1 — ja3c04893_si_001.pdf [file ja3c04893_si_001.pdf]

Supporting Information

for

Cu_4S Cluster in "0-Hole" and "1-Hole" States: Geometric and Electronic Structure Variations for the Active Cu_Z* Site of N_2O Reductase

Yang Liu,[†,*] Sayanti Chatterjee,[§] George E. Cutsail III,[§,‡] Sergey Peredkov,[§] Sandeep K. Gupta,[†] Sebastian Dechert,[†] Serena DeBeer,[§] Franc Meyer[†,#,*]

[†] Institute of Inorganic Chemistry, University of Göttingen, Tammannstraße 4, 37077 Göttingen, Germany

[§] Max Planck Institute for Chemical Energy Conversion, Stiftstrasse 34–36, 45470 Mülheim an der Ruhr, Germany

[‡] Institute of Inorganic Chemistry, University of Duisburg-Essen, Universitätsstraße 7, 45117 Essen, Germany

[#] International Center for Advanced Studies of Energy Conversion (ICASEC), University of Göttingen, Tammannstraße 6, 37077 Göttingen, Germany

franc.meyer@chemie.uni-goettingen.de; yang.liu@chemie.uni-regensburg.de

Table of Contents

Experimental Procedures S3

General Considerations S3

Synthesis of **2** and **3** S3

Crystallographic Data S5

NMR Spectra S9

Variable-Temperature ^1H NMR spectra S15

ESI Mass Spectra of **2** S17

IR Spectra of **2** and **3** S18

Magnetic Measurements S19

EPR Spectroscopy S20

Electrochemistry S22

UV-Vis-NIR spectroscopy S23

DFT Calculations S25

XES and XAS Data and Spectral Calculations S29

Reactivity Study of Complex $[L_2Cu_4S](PF_6)_2$ (**2**) S34

Reaction of **2** with N_2O S34

Reaction of **2** with $[^nBu_4N]N_3$ and $[^nBu_4N]NO_2$ S34

Reaction of **2** with KI S34

Reaction of **2** with $[Me_3O]BF_4$ S38

References S55

Experimental Procedures

General Considerations

All manipulations of air- and moisture-sensitive materials were performed under an atmosphere of dry dinitrogen with the rigorous exclusion of air and moisture using standard Schlenk techniques, or in a N_2-filled glovebox. Solvents were dried with sodium (Et_2O) in the presence of benzophenone or CaH_2 (MeCN) and were freshly distilled and degassed prior to use. Toluene, acetone, d_6-acetone, d_3-MeCN and d_6-dimethyl sulfoxide (d_6-DMSO) were dried with 3 Å molecular sieves. $[LCu_2](PF_6)_2$[1] and $[Cp^*_2Fe]PF_6$ (Cp* = pentamethylcyclopentadienyl)[2] were synthesized according to literature procedures. $[^nBu_4N]PF_6$ was dried in vacuo at 80 °C for 2 d, then transferred to the glovebox prior to use. All other chemicals were purchased from Sigma-Aldrich (Germany) and used as received. ^1H, ^{13}C, ^{31}P and ^{19}F NMR spectra were recorded with Bruker 300 MHz or 400 MHz spectrometer. All chemical shifts are reported in units of ppm and referenced to the residual ^1H NMR signals of the deuterated solvents for proton chemical shifts, the ^{13}C NMR signal of deuterated solvents for carbon chemical shifts, the ^{31}P NMR signal of 85% phosphorous acid (external standard) for phosphorous chemical shifts, and the ^{19}F NMR signal of $CFCl_3$ (external standard) for fluorine chemical shifts. ESI mass spectra were recorded on a Bruker HCT ultra spectrometer. Elemental analyses were performed by the analytical laboratory of the Institute of Inorganic Chemistry at the University of Göttingen using an Elementar Vario EL III instrument. UV-vis-NIR spectra were recorded with an Agilent Cary 60 equipped with an Unisoku Cryostat (CoolSpek) and magnetic stirrer using quartz cuvettes with an attached tube and a screw cap with a septum. IR spectra were recorded inside a glovebox on a Cary 630 FTIR spectrometer equipped with ZnSe ATR module and analyzed by FTIR MicroLab software.

Synthesis of 2 and 3

Scheme S1. Synthetic route for **2** and **3**.

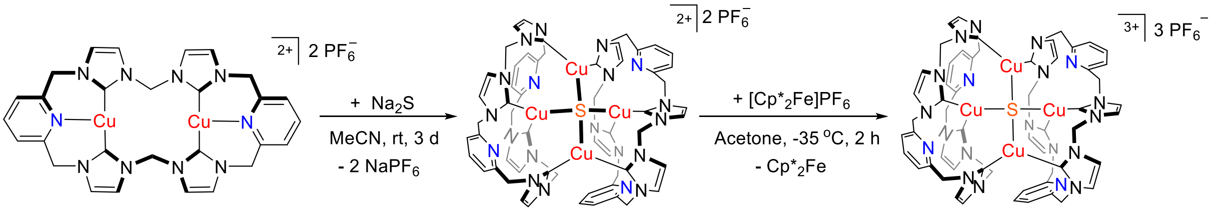

Synthesis of $[L_2Cu_4S](PF_6)_2$ (2). At room temperature, to the MeCN (7 mL) solution of $[LCu_2](PF_6)_2$ (**1**, 55 mg, 0.06 mmol) was added Na_2S (14 mg, 0.18 mmol). The suspension was stirred at room temperature for 3 days, and during this time the color of the suspension changed from orange to yellow. After centrifugation, the upper yellow solution was collected and left for crystallization through Et_2O diffusion at room temperature. Yellow crystals of **2** were obtained in 80% yield (38 mg). ^1H NMR (400 MHz, d_3-MeCN, 298 K): δ (ppm) 8.32 (d, J = 12.4 Hz, 4H, NHC–CH_2–NHC), 7.62 (t, J = 8.0 Hz, 4H, CH–p–Py), 7.34 (d, J = 7.6 Hz, 4H, CH–m–Py), 7.22 (d, J = 2.0 Hz, 4H, CH-imidazolyl backbone), 7.19 (d, J = 1.6 Hz, 4H, CH-imidazolyl backbone), 7.03 (d, J = 7.6 Hz, 4H, CH–m–Py), 6.88 (d, J = 2.0 Hz, 4H, CH-imidazolyl backbone), 6.81 (d, J = 1.6 Hz, 4H, CH-imidazolyl backbone), 5.29/5.16 (ABq, 8H, J = 16 Hz, Py–CH_2–NHC), 5.00/4.93 (ABq, 8H, J = 12.8 Hz, Py–CH_2–NHC), 4.78 (d, J = 12.4 Hz, 4H, NHC–CH_2–NHC). ^1H NMR (400 MHz, d_6-Acetone, 298 K): δ (ppm) 8.52 (d, J = 12.4 Hz, 4H, NHC–CH_2–NHC), 7.68 (t, J = 7.6 Hz,

4H, C*H*–*p*–Py), 7.51 (d, *J* = 7.6 Hz, 4H, C*H*–*m*–Py), 7.50 (d, *J* = 2.0 Hz, 4H, C*H*-imidazolyl backbone), 7.42 (d, *J* = 1.6 Hz, 4H, C*H*-imidazolyl backbone), 7.13 (d, *J* = 7.6 Hz, 4H, C*H*–*m*–Py), 7.10 (d, *J* = 2.0 Hz, 4H, C*H*–imidazolyl backbone), 6.99 (d, *J* = 1.6 Hz, 4H, C*H*-imidazolyl backbone), 5.49/5.34 (ABq, 8H, *J* = 15.6 Hz, Py–CH_2–NHC), 5.18/5.03 (ABq, 8H, *J* = 12.4 Hz, Py–CH_2–NHC), 5.43 (d, *J* = 12.4 Hz, 4H, NHC–CH_2–NHC). ^{1}H NMR (300 MHz, d_6-DMSO, 298 K): δ (ppm) 8.10 (d, *J* = 12.2 Hz, 4H, NHC–CH_2–NHC), 7.65 (t, *J* = 7.6 Hz, 4H, C*H*–*p*–Py), 7.53 (d, *J* = 1.8 Hz, 4H, C*H*-imidazolyl backbone), 7.51–7.44 (m, 8H, C*H*-imidazolyl backbone and C*H*-*m*-Py), 7.11–7.02 (m, 8H, C*H*-imidazolyl backbone and C*H*-*m*-Py), 6.98 (d, *J* = 1.8 Hz, 4H, C*H*-imidazolyl backbone), 5.26/5.18 (ABq, 8H, *J* = 15.9 Hz, Py–CH_2–NHC), 4.95/4.87 (ABq, 8H, *J* = 12.3 Hz, Py–CH_2–NHC), 4.81 (d, *J* = 12.2 Hz, 4H, NHC–CH_2–NHC). See Figures S4-S6 for a more detailed assignment of these peaks. ^{13}C NMR (100 MHz, d_3-MeCN, 298 K): δ (ppm) 191.4/188.7 (N*C*N), 157.1/157.0 (*C*H-*o*-Py), 138.4 (*C*H–*p*–Py), 122.7/122.6 (*C*H-imidazolyl backbone), 122.3/121.1 (*C*H–*m*–Py), 119.6/119.0 (*C*H-imidazolyl backbone), 61.5 (NHC–*C*H$_2$–NHC), 57.2/54.4 (Py–*C*H$_2$–NHC). See Figure S7 for a more detailed assignment of these peaks. ^{19}F NMR (376 MHz, d_3-MeCN, 298 K): δ (ppm) −72.8 (d, $J_{P\text{–}F}$ = 706.9 Hz). ^{31}P NMR (162 MHz, d_3-MeCN, 298 K): δ (ppm) −144.6 (hepta, $J_{F\text{–}P}$ = 707.3 Hz). Anal. Calcd. for $C_{56}H_{52}Cu_4N_{20}P_2F_{12}$·2MeCN: C 43.32, H 3.51, N 18.52, S 1.93; Found: C 43.53, H 3.71, N 18.13, S 2.14. ESI–MS (MeCN) m/z (%): 645.1 (100) for $[C_{56}H_{52}Cu_4N_{20}]^{2+}$. Absorption spectrum (acetone, −35 °C): λ_{max}, nm (ε, M^{-1} cm^{-1}) 380 (shoulder, 10000), 430 (shoulder, 3700). ATR–IR (powder, cm^{-1}): ν = 3165 (w), 3135 (w), 3127 (w), 2928 (w), 1588 (w), 1572 (w), 1457 (w), 1435 (w), 1383 (w), 1344 (w), 1317 (w), 1221(m), 1210 (w), 1195 (w), 1168 (w), 1156 (w), 1100 (w), 1041 (w), 994 (w), 976 (w), 956 (w), 917 (w), 876 (w), 840 (s), 829 (s), 785 (m), 762 (m), 730 (m), 723 (m), 714 (m), 705 (m), 685 (w), 662 (w), 652 (w).

Synthesis of [L$_2$Cu$_4$S](PF$_6$)$_3$ (3). At −35 °C, to the acetone (3 mL) solution of [L$_2$Cu$_4$S](PF$_6$)$_2$ (**2**, 15.8 mg, 0.01 mmol) was added a precooled acetone (1 mL) solution of [Cp*$_2$Fe]PF$_6$ (5.0 mg, 0.11 mmol). The color of the solution changed from yellow to purple immediately. The reaction mixture was stored in the glovebox freezer (−35 °C) for 2 hours and then poured into a large amount of cold Et$_2$O (30 mL). After decanting the upper yellowish solution, the resulting purple precipitate was washed with Et$_2$O (3 mL × 2) until the washing liquid becomes almost colorless. The purple solid was put in the glovebox freezer (−35 °C) for 20 mins and then extracted with cold acetone/toluene (2 mL, v/v = 4/1) and fast filtered into a glass tube surrounded by cold Et$_2$O in a big glass vial. Purple crystals of **3** (10.7 mg, 62%) were obtained after crystallization by layering hexane (8 mL) on top of this acetone/toluene solution at −35 °C. *Caution: Complex **3** in solution is thermally sensitive. All the glassware used in the workup should be precooled (−35 °C), and the solution was always kept at −35 °C or only shortly under the glovebox environment using a cooling aluminum block.* ^{1}H NMR (400 MHz, d_6-acetone, 263 K): δ (ppm) 17.45 (br), 13.13 (br), 9.54 (br), 7.93 (br), 7.56 (br), 7.39 (br), 6.81 (vbr), 6.25 (vbr), 4.63 (br), 2.97 (br). Anal. Calcd. for $C_{56}H_{52}Cu_4N_{20}P_3F_{18} \cdot C_3H_6O$: C 39.71, H 3.28, N 15.70, S 1.80; Found: C 39.80, H 3.40, N 15.49, S 2.15. Absorption spectrum (acetone, −35 °C): λ_{max}, nm (ε, M^{-1} cm^{-1}) 360 (4700), 375 (shoulder, 3100), 450 (broad, 270), 530 (broad, 1060), 790 (8260). ATR–IR (powder, cm^{-1}): ν = 3173 (w), 3137 (w), 3111 (w), 2937 (w), 1591 (w), 1574 (w), 1454 (w), 1440 (w), 1412 (w), 1389 (w), 1377 (w), 1344 (w), 1321(w), 1226 (m), 1197 (w), 1159 (w), 1109 (w), 1092 (w), 1046 (w), 995 (w), 978 (w), 956 (w), 917 (w), 874 (w), 828 (s), 785 (m), 763 (m), 738 (m), 685 (w), 667 (w).

Synthesis of [L$_2$Cu$_4$S](BF$_4$)$_3$ (3-BF$_4$). In a 8 mL glass vial, a mixture of [L$_2$Cu$_4$S](PF$_6$)$_2$ (**2**, 15.8 mg, 0.01 mmol) and NaBF$_4$ (11.0 mg, 0.1 mmol) was dissolved in acetone (5 mL) and was stirred at room temperature for 3 h. The resulting suspension was put in the glovebox freezer (−35 °C) for 30 min and then was poured into the precooled acetone (1 mL) solution of [Cp*$_2$Fe]PF$_6$ (5.0 mg, 0.11 mmol). The color of the suspension changed from

yellow to purple immediately. The reaction mixture was stored in the glovebox freezer (−35 °C) for 3 hours, during which course the vial was shaken violently every 30 min, to form a large amount of purple precipitate. After decanting the upper yellowish solution, the resulting purple precipitate was washed with Et_2O (3 mL × 3) until the washing liquid becomes almost colorless. The purple solid was put in the glovebox freezer (−35 °C) for 30 mins and then extracted with cold MeCN (2 ml) and fast filtered into a glass tube surrounded by cold Et_2O in a big glass vial. Purple crystals of **3-BF$_4$** (8.7 mg, 56%) were obtained after crystallization by layering Et_2O (8 mL) on top of this MeCN solution at −35 °C. **3-BF$_4$** could also be synthesized through counterion exchange in 65% yield by adding the acetone solution (3 mL) of **3** (17.3 mg, 0.01 mmol) to an acetone solution (3 mL) of $NaBF_4$ (11.0 mg, 0.1 mmol) at −35 °C. A similar workup procedure as described above after adding the oxidant was then followed. *Caution: Complex **3-BF$_4$** in solution is thermally sensitive. All the glassware used in the workup should be precooled (-35 °C), and the solution was always kept at −35 °C or only shortly under the glovebox environment using a cooling aluminum block.* ^{1}H NMR (in d_3-MeCN; Figure S15) and ATR-IR (powder; Figure S21) spectra are similar to those of complex **3**.

Crystallographic Data

X-Ray Structure Determination. Crystal data and details of the data collections are given in Tables S1, selected bond lengths and angles in Tables S2, molecular structures are shown in Figures S1–S3, S36 and S43. X-ray data were collected on a STOE IPDS II or a BRUKER D8-QUEST diffractometer (monochromated Mo-Kα radiation, λ = 0.71073 Å) by use of ω or ω and ϕ scans at low temperature. The structures were solved with SHELXT and refined on F^2 using all reflections with SHELXL.[3,4] Most non-hydrogen atoms were refined anisotropically. Hydrogen atoms were placed in calculated positions and assigned to an isotropic displacement parameter of 1.2 or 1.5 U_{eq}(C). Face-indexed absorption corrections were performed numerically with the program X-RED[5] or by the multi-scan method with SADABS.[6]

In **2**, PF_6^- was found to be disordered about two positions (occupancy factors: 0.884(2) / 0.116(2)) and Et_2O was found to be disordered about a center of inversion (refined at ½ occupancy). EADP constraints and SAME restraints were applied in case of PF_6^- and DFIX (d(C-O) = 1.43 Å, d(C-C) = 1.51 Å), SADI (d(H_3C···O)), RIGU, SIMU, DELU restraints in case of Et_2O.

In **3-BF$_4$**, two BF_4^- (occupancy factors: 0.695(4) / 0.305(4) & 0.750(4) / 0.250(4)) and one C_5H_3N group (occupancy factors: 0.718(7) / 0.282(7)) were found to be disordered. SAME restraints and EADP constraints were applied in case of one BF_4^- for the other SADI (d(B-F), d(F···F)) restraints and EADP constraints were applied, for the disordered C_5H_3N group SADI (d(H_2C-C^{ar})), SAME, SIMU and FLAT restraints. The unit cell of **3-BF$_4$** contains highly disordered solvent molecules (MeCN) for which no satisfactory model for a disorder could be found. The solvent contribution to the structure factors was calculated with PLATON SQUEEZE[7] and the resulting .fab file was processed with SHELXL using the ABIN instruction. The empirical formula and derived values are in accordance with the calculated cell content.

In **4**, one MeCN was found to be disordered about two positions (occupancy factors: 0.165(10) / 0.835(10)) and was refined using SAME restraints and EADP constraints. A disorder of two MeCN and one Et_2O was refined using fixed occupancy factors (MeCN : Et_2O : MeCN = 0.5 : 0.3 : 0.2) and SAME (for MeCN), DFIX (for Et_2O; d(C-C) = 1.51 Å, d(C-O = 1.43 Å, d(C···C/O = 2.2 Å)) restraints and EADP constraints. The atoms were refined isotropically.

5 was refined as non-merohedral twin with a BASF of 0.1175(9).

Table S1. Crystal data and refinement details for **2**, **3-BF$_4$**, **4** and **5**.

**Compound**	**2**	**3-BF$_4$**	**4**	**5**
empirical formula	$C_{60}H_{60}Cu_4F_{12}N_{21}O_{0.50}P_2S$	$C_{64}H_{64}B_3Cu_4F_{12}N_{24}S$	$C_{64.60}H_{66.10}Cu_4I_2N_{23.70}O_{0.30}S$	$C_{32}H_{33.50}Cu_2F_6N_{11.50}PS$
moiety formula	$C_{56}H_{52}Cu_4N_{20}S^{2+}$, 2($PF_6^-$), 0.5($C_4H_{10}O$), C_2H_3N	$C_{56}H_{52}Cu_4N_{20}S^{3+}$, 3($BF_4^-$), 4($C_2H_3N$)	$C_{56}H_{52}Cu_4N_{20}S^{2+}$, 2($I^-$), 3.7($C_2H_3N$), 0.3($C_4H_{10}O$)	$C_{29}H_{29}Cu_2N_{10}S^+$, PF_6^-, 1.5(C_2H_3N)
formula weight	1659.45	1716.04	1719.33	883.31
T [K]	133(2)	100(2)	133(2)	133(2)
crystal size [mm^3]	0.394 x 0.183 x 0.166	0.480 x 0.188 x 0.146	0.220 x 0.160 x 0.140	0.500 x 0.070 x 0.060
crystal system	triclinic	triclinic	triclinic	monoclinic
space group	P–1 (No. 2)	P–1 (No. 2)	P–1 (No. 2)	$C2/c$ (No. 15)
a [Å]	13.3729(5)	14.2508(4)	12.4715(3)	27.7088(10)
b [Å]	15.1431(6)	15.7435(5)	12.6794(3)	9.7213(2)
c [Å]	18.6725(6)	18.3107(6)	22.9321(6)	26.9238(10)
α [°]	79.152(3)	89.0050(10)	76.512(2)	90
β [°]	75.928(3)	88.6670(10)	86.049(2)	99.682(3)
γ [°]	64.273(3)	79.6190(10)	87.149(2)	90
V [Å^3]	3289.4(2)	4039.4(2)	3515.76(15)	7149.0(4)
Z	2	2	2	8
ρ [g·cm^{-3}]	1.675	1.411	1.624	1.641
F(000)	1682	1742	1720	3592
μ [mm^{-1}]	1.450	1.146	2.160	1.368
T_{min} / T_{max}	0.7025 / 0.9070[a]	0.75 / 0.85[b]	0.5535 / 0.8017[a]	0.7468 / 0.9403[a]
θ-range [°]	1.499 – 26.850	2.070 – 27.968	1.653 – 26.900	1.491 – 26.868
hkl–range	±16, ±19, ±23	±18, ±20, ±24	−13 to 15, ±16, ±28	−35 to 34, −12 to 11, ±34
measured refl.	35014	105360	49682	31187
unique refl. [R_{int}]	13922 [0.0238]	19385 [0.0546]	14882 [0.0265]	7602 [0.0563]
observed refl. ($I > 2\sigma(I)$)	11596	13286	12628	5831
data / restr. / param.	13922 / 78 / 953	19385 / 205 / 1061	14882 / 37 / 884	7602 / 0 / 488
goodness–of–fit (F^2)	1.037	1.044	1.111	1.087
$R1$, $wR2$ ($I > 2\sigma(I)$)	0.0303 / 0.0730	0.0566 / 0.1361	0.0311 / 0.0756	0.0408 / 0.0872
$R1$, $wR2$ (all data)	0.0414 / 0.0783	0.0879 / 0.1549	0.0422 / 0.0818	0.0664 / 0.0983
res. el. dens. [e·Å^{-3}]	−0.444 / 0.554	−0.921 / 1.395	−1.188 / 1.228	−0.328 / 0.501

[a]X-RED. [b]SADABS.

Table S2. Selected distances [Å] and angles [°].

	**2**	**3-BF$_4$**	**4**	**5**
Cu–C	1.950(2)–1.991(2)	1.950(3)–1.972(4)	1.957(3)–1.987(3)	1.935(3)–1.968(3)
Cu-N				2.443(3) / 2.610(3)
Cu–S1	2.3021(6)–2.3499(5)	2.1982(9)–2.3215(9)	2.3173(7)–2.3402(7)	2.2996(10) / 2.3056(10)
Cu···Cu	2.9718(5)–4.5867(4)	3.0952(6)–4.6374(7)	2.9818(5)–4.5559(6)	2.9794(6)
S1···Cu4[a]	0.6359(6)	0.1476(8)	0.6479(6)	
C–Cu–C	117.20(8)–130.37(8)	129.99(15)–131.68(16)	118.93(12)–130.73(11)	130.06(14) / 136.83(14)
Cu–S1–Cu	80.274(18)–160.80(3)	86.41(3)–175.76(5)	79.32(2)–156.22(4)	80.63(3)

[a]distance of the sulfur atom to the plane of the four copper atoms

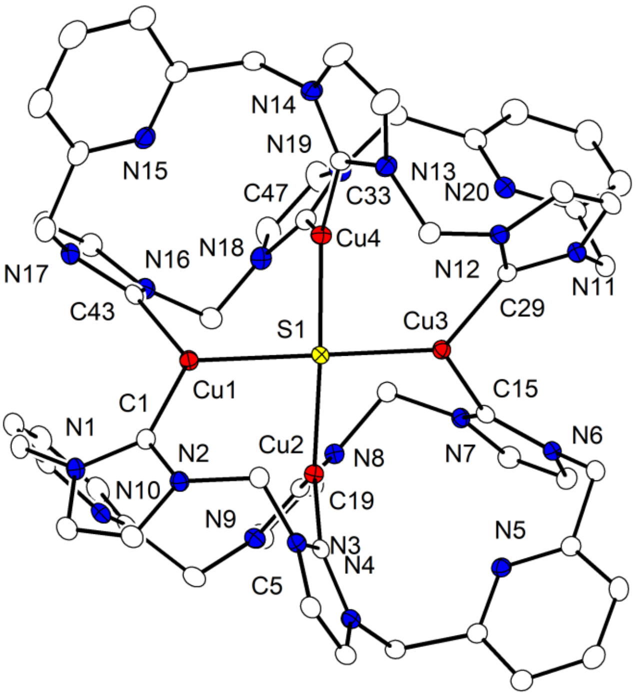

Figure S1. Plot (30% probability thermal ellipsoids, hydrogen atoms omitted) of the molecular structure of the cationic part of **2**. Selected bond lengths [Å] and angles [°]: Cu1–C43 1.961(2), Cu1–C1 1.973(2), Cu1–S1 2.3499(5), Cu2–C19 1.965(2), Cu2–C5 1.991(2), Cu2–S1 2.3187(6), Cu3–C15 1.962(2), Cu3–C29 1.978(2), Cu3–S1 2.3021(6), Cu4–C47 1.950(2), Cu4–C33 1.962(2), Cu4–S1 2.3083(6), Cu1···Cu2 3.1579(4), Cu1···Cu3 4.5867(4), Cu1···Cu4 4.5867(4), Cu2···Cu3 3.1883(4), Cu2···Cu4 4.2703(5), Cu3···Cu4 2.9719(4); C43–Cu1–C1 126.67(9), C43–Cu1–S1 118.11(6), C1–Cu1–S1 114.97(6), C19–Cu2–C5 130.37(8), C19–Cu2–S1 120.92(6), C5–Cu2–S1 108.67(6), C15–Cu3–C29 117.20(8), C15–Cu3–S1 125.88(6), C29–Cu3–S1 116.92(6), Cu3–S1–Cu4 80.274(18), Cu3–S1–Cu2 87.261(19), Cu4–S1–Cu2 134.71(3), Cu3–S1–Cu1 160.80(3), Cu4–S1–Cu1 92.67(2), Cu2–S1–Cu1 85.127(19).

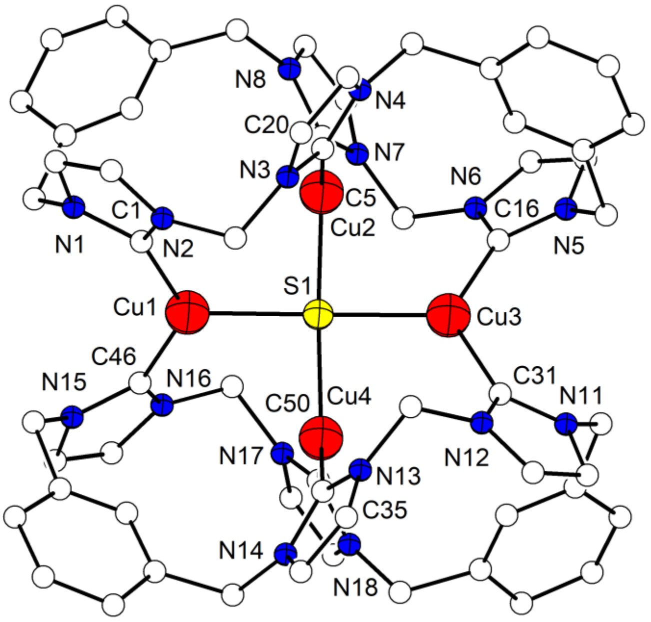

Figure S2. Plot (hydrogen atoms omitted) of the molecular structure of the cationic part of **3** showing the arrangement of the atoms. Selected bond lengths and angles are not given because of the low data quality ($R1$ =

0.18). Crystal data: monoclinic, $P2_1/n$, a = 14.7091(5) Å, b = 32.8214(10) Å, c = 19.3446(6) Å, β = 92.302(3)°, V = 9331.5(5) Å^3, Z = 4.

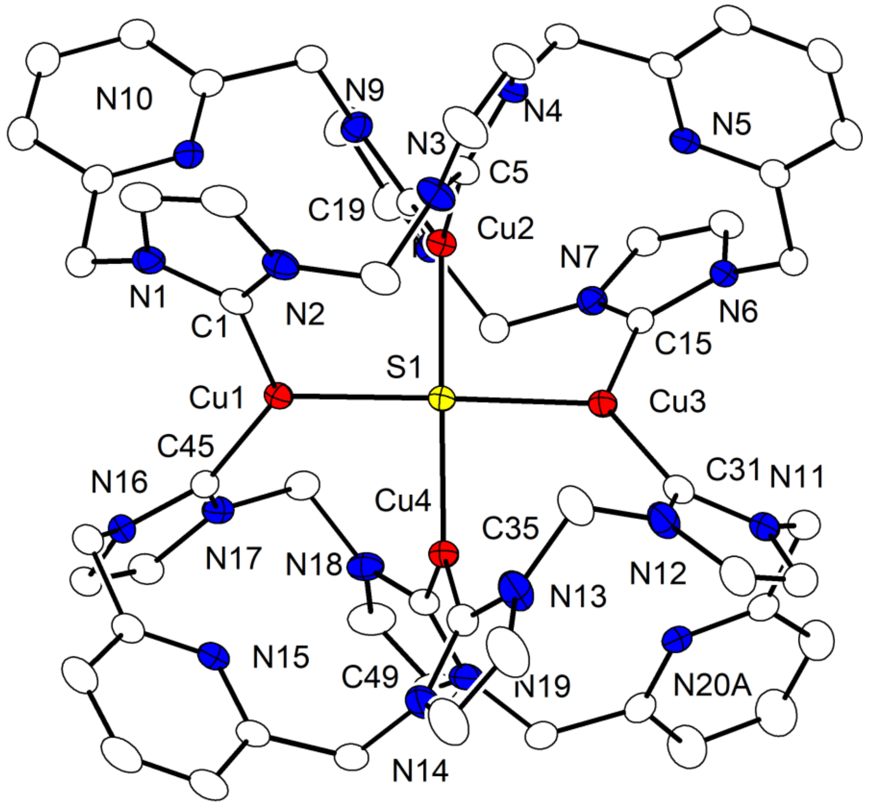

Figure S3. Plot (30% probability thermal ellipsoids, hydrogen atoms omitted) of the molecular structure of the cationic part of **3-BF$_4$**. Selected bond lengths [Å] and angles: Cu1–C45 1.950(3), Cu1–C1 1.960(4), Cu1–S1 2.3215(9), Cu2–C19 1.956(3), Cu2–C5 1.960(4), Cu2–S1 2.1982(9), Cu3–C31 1.956(4), Cu3–C15 1.957(3), Cu3–S1 2.3191(9), Cu4–C49 1.950(4), Cu4–C35 1.972(4), Cu4–S1 2.2005(9), Cu1···Cu2 3.1898(6), Cu1···Cu3 4.6374(7), Cu1···Cu4 3.2654(6), Cu2···Cu3 3.2129(6), Cu2···Cu4 4.3772(6), Cu3···Cu4 3.0952(6); C45–Cu1–C1 129.99(15), C45–Cu1–S1 115.81(10), C1–Cu1–S1 114.19(11), C19–Cu2–C5 131.33(14), C19–Cu2–S1 113.10(10), C5–Cu2–S1 115.42(11), C31–Cu3–C15 130.31(16), C31–Cu3–S1 115.63(12), C15–Cu3–S1 114.06(10), C49–Cu4–C35 131.68(16), C49–Cu4–S1 115.28(11), C35–Cu4–S1 113.02(13), Cu2–S1–Cu4 168.70(5), Cu2–S1–Cu3 90.63(3), Cu4–S1–Cu3 86.41(3), Cu2–S1–Cu1 89.74(3), Cu4–S1–Cu1 92.42(3), Cu3–S1–Cu1 175.76(5).

NMR Spectra

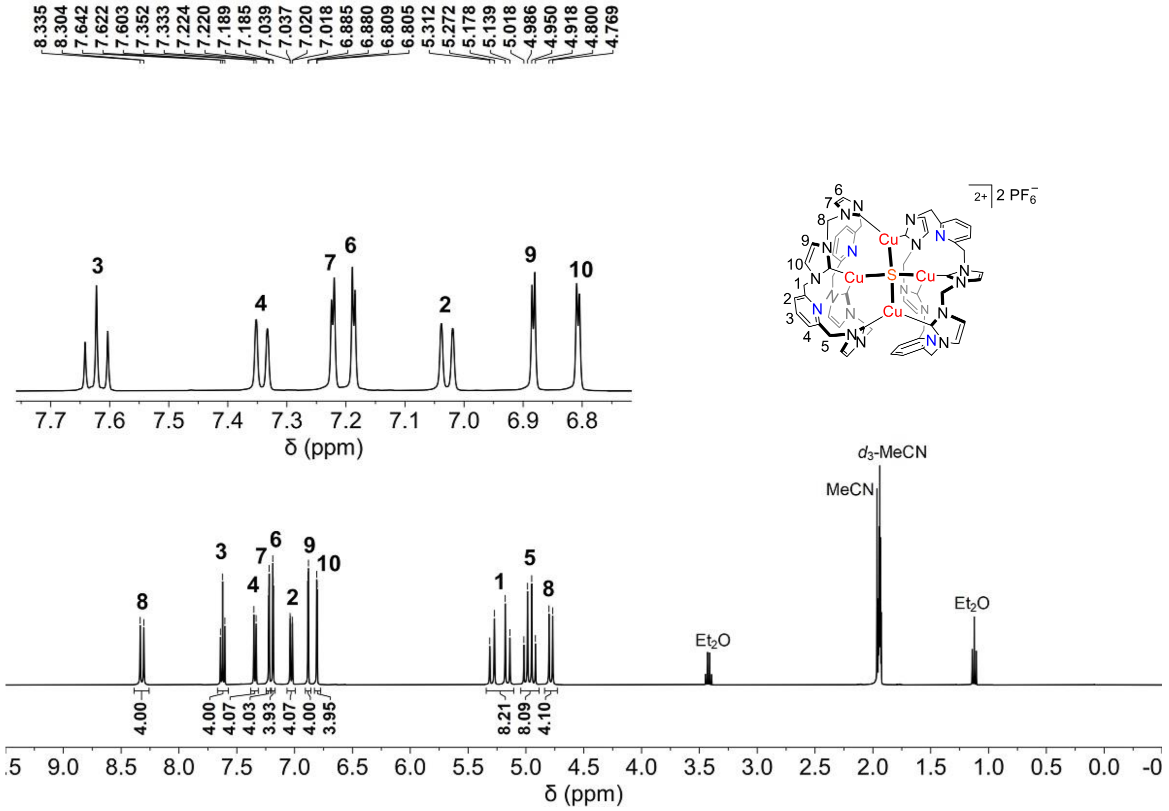

Figure S4. ^{1}H NMR spectrum of $[L_2Cu_4S](PF_6)_2$ (**2**) in d_3-MeCN (400 MHz, 298 K).

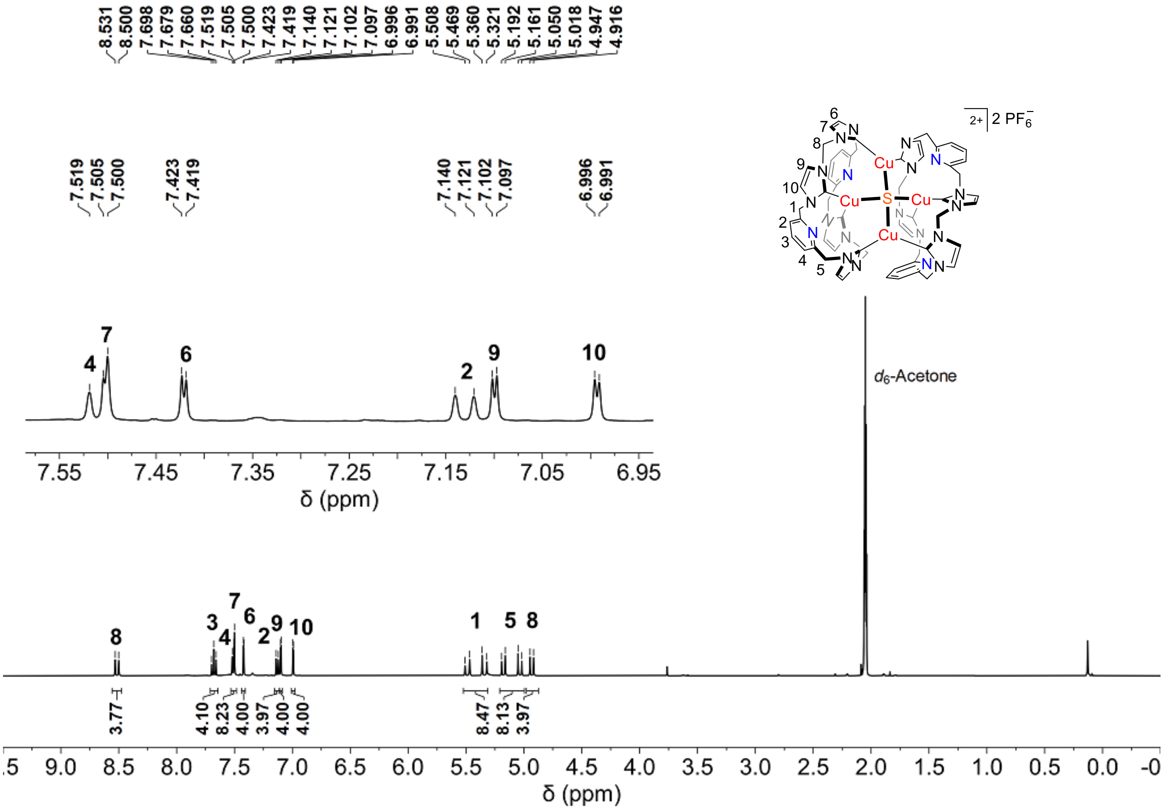

Figure S5. ^{1}H NMR spectrum of $[L_2Cu_4S](PF_6)_2$ (**2**) in d_6-Acetone (400 MHz, 298 K).

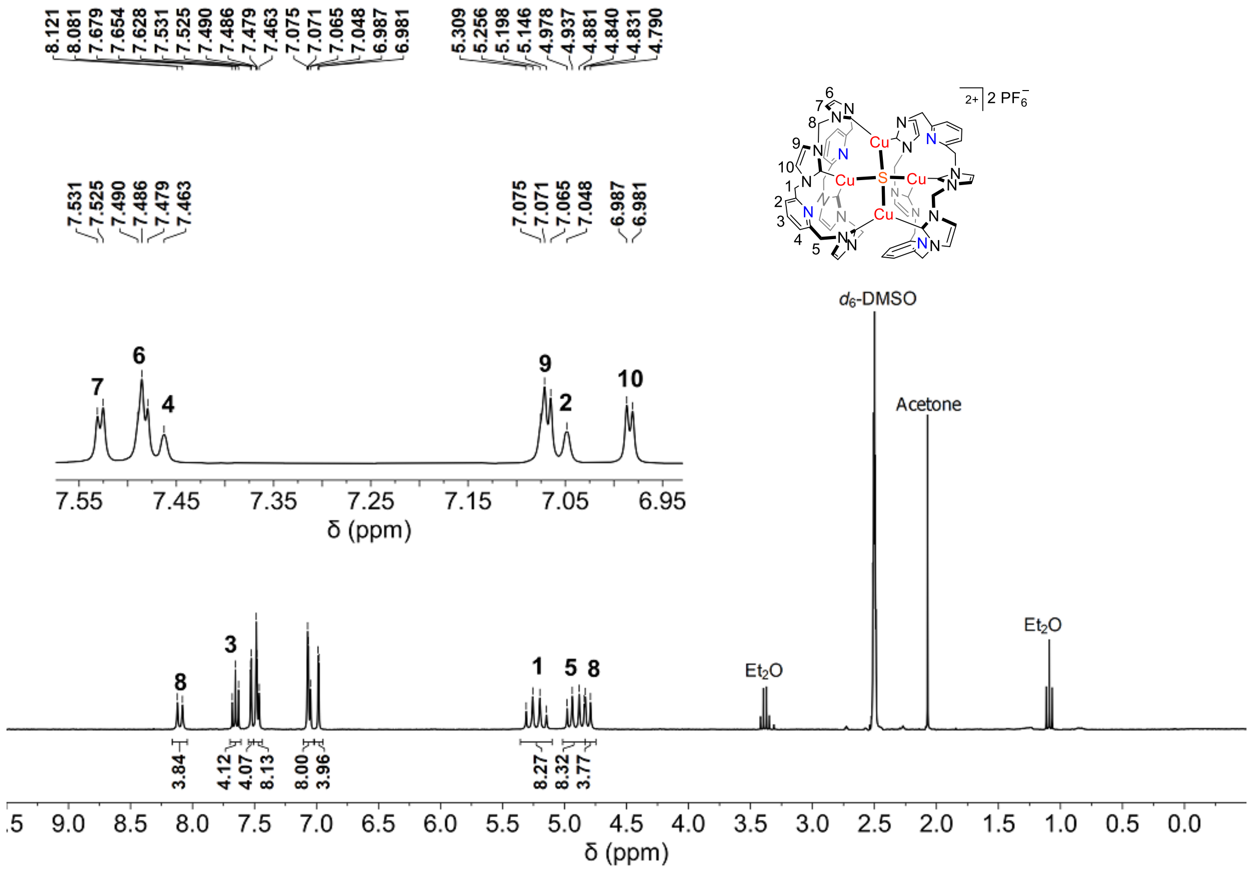

Figure S6. ^{1}H NMR spectrum of $[L_2Cu_4S](PF_6)_2$ (**2**) in d_6-DMSO (300 MHz, 298 K).

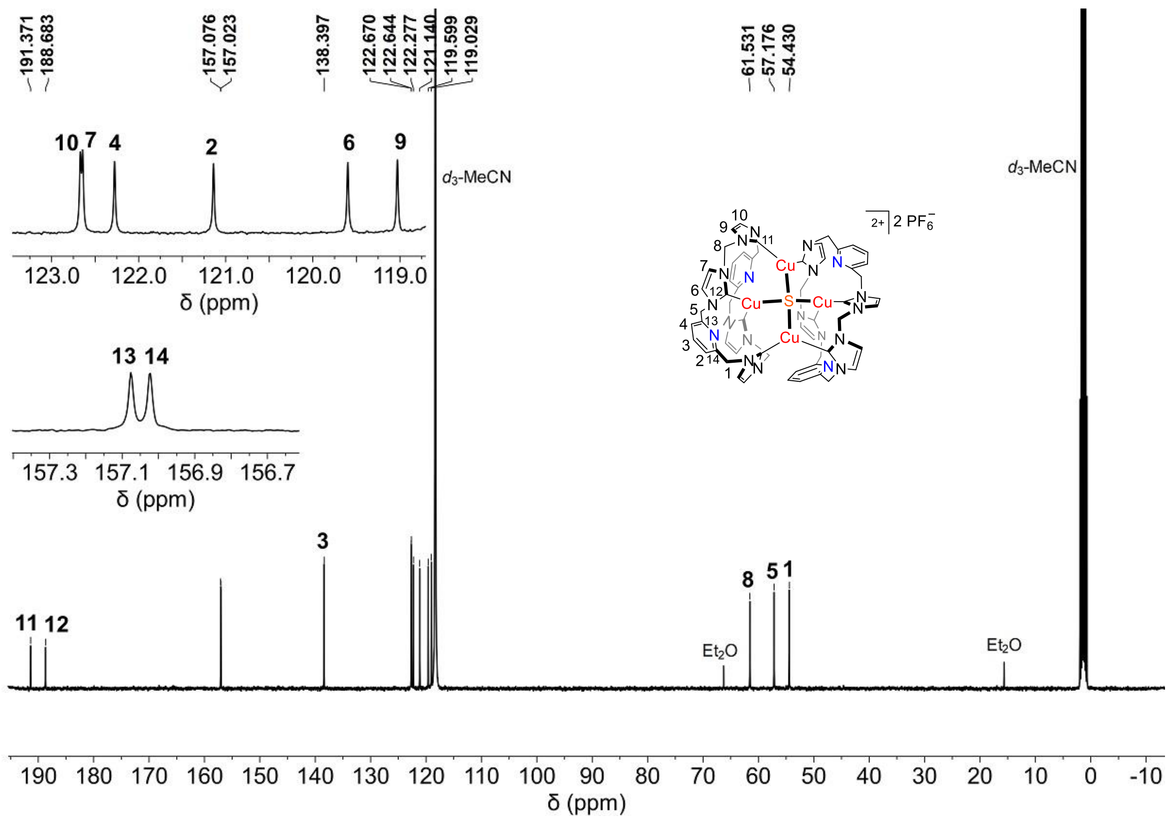

Figure S7. ^{13}C NMR spectrum of $[L_2Cu_4S](PF_6)_2$ (**2**) in d_3-MeCN (100 MHz, 298 K).

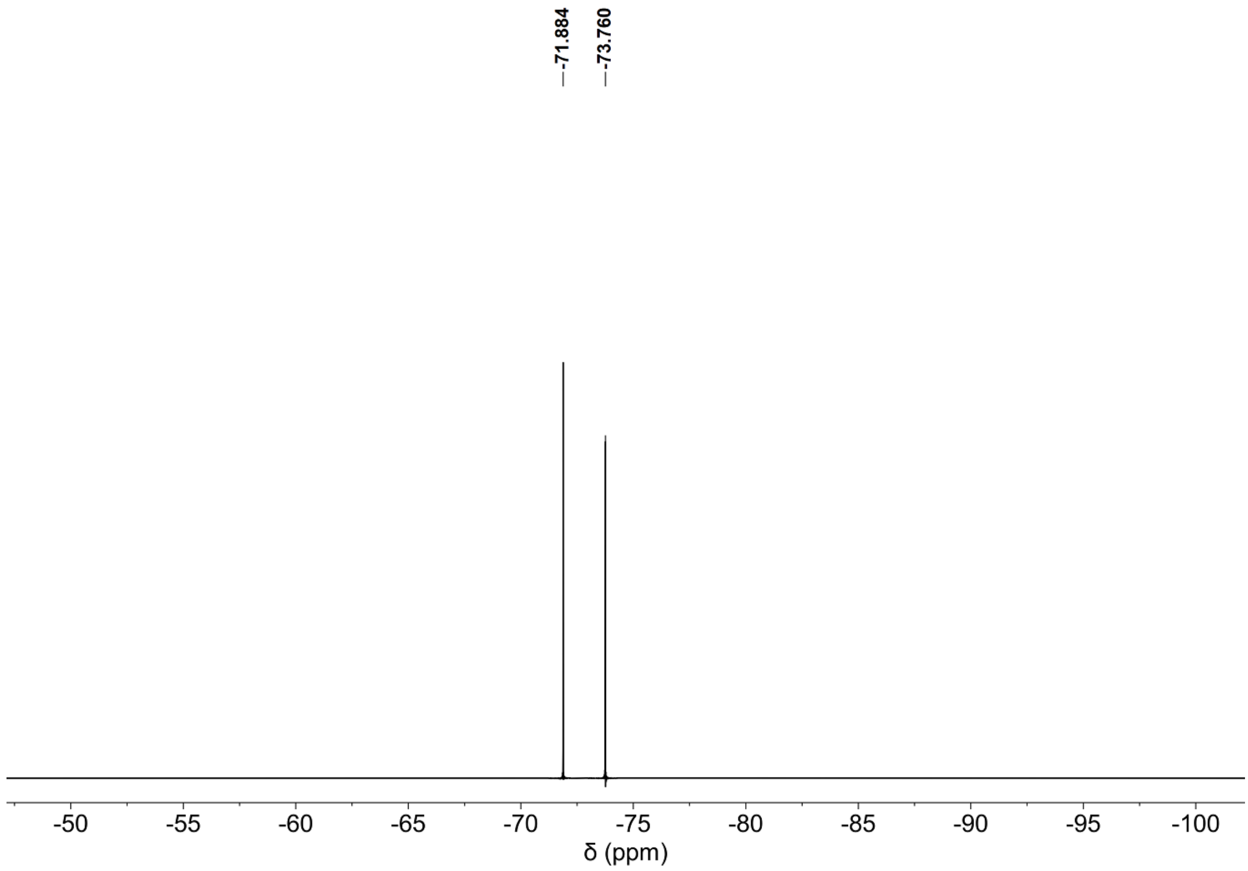

Figure S8. ^{19}F NMR spectrum of $[L_2Cu_4S](PF_6)_2$ (**2**) in d_3-MeCN (376 MHz, 298 K).

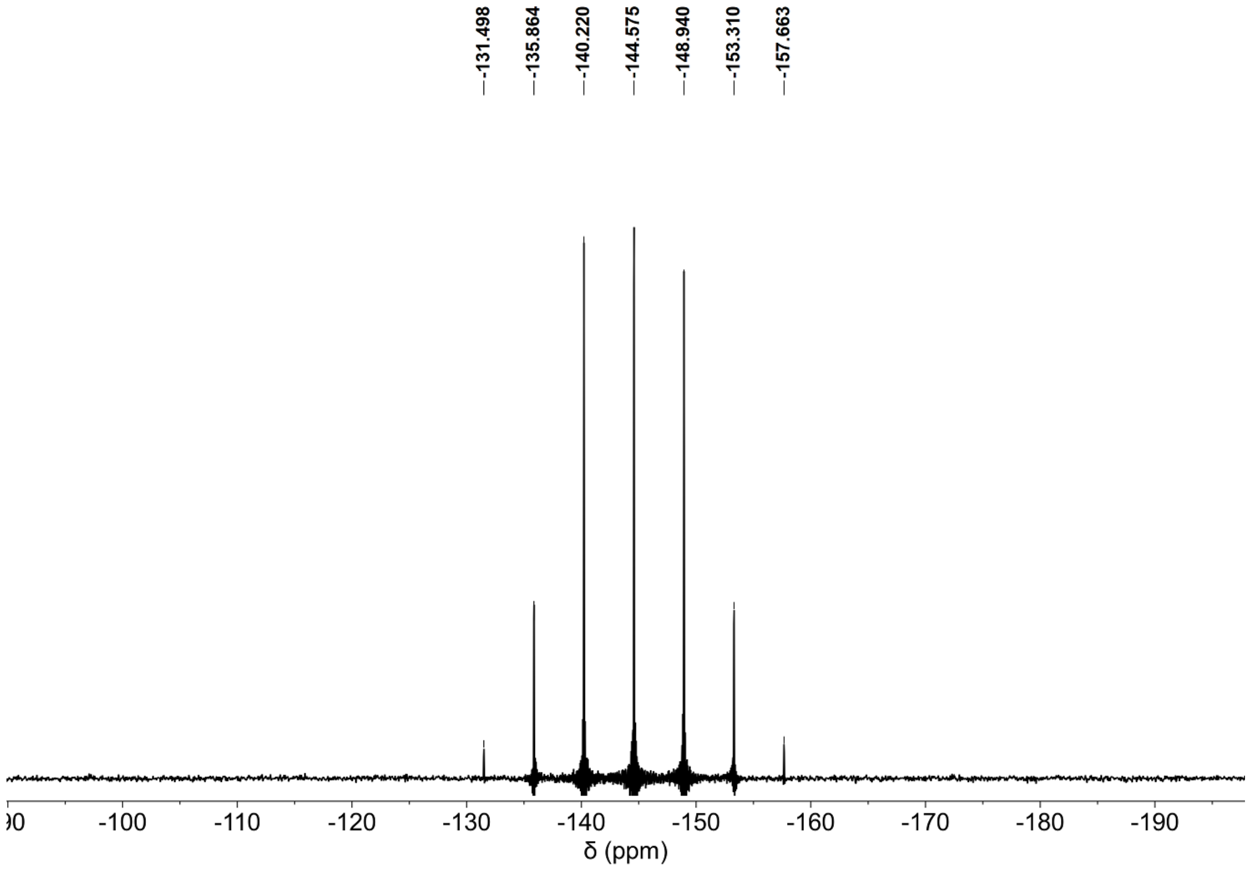

Figure S9. ^{31}P NMR spectrum of $[L_2Cu_4S](PF_6)_2$ (**2**) in d_3-MeCN (162 MHz, 298 K).

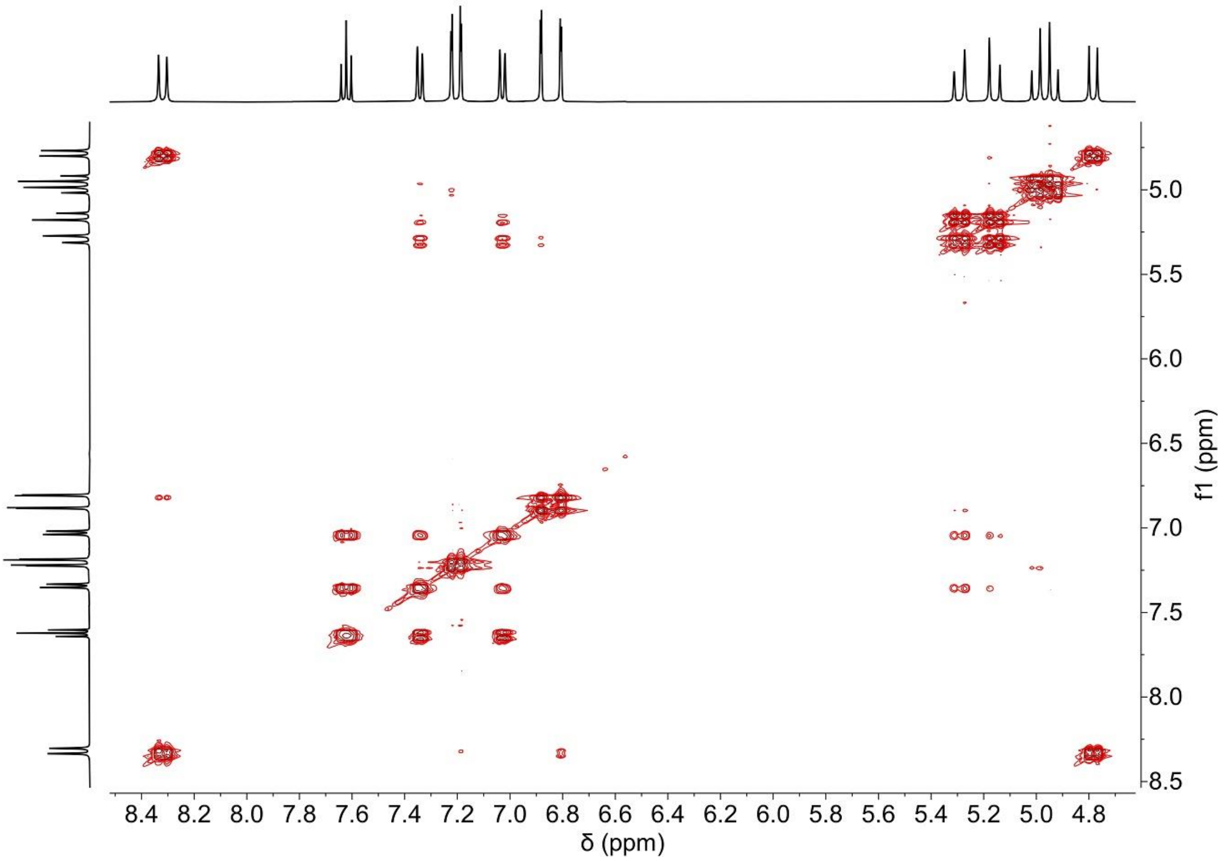

Figure S10. ^{1}H-^{1}H COSY spectrum of [L_2Cu_4S](PF_6)$_2$ (**2**) in d_3-MeCN (400, 400 MHz, 298 K).

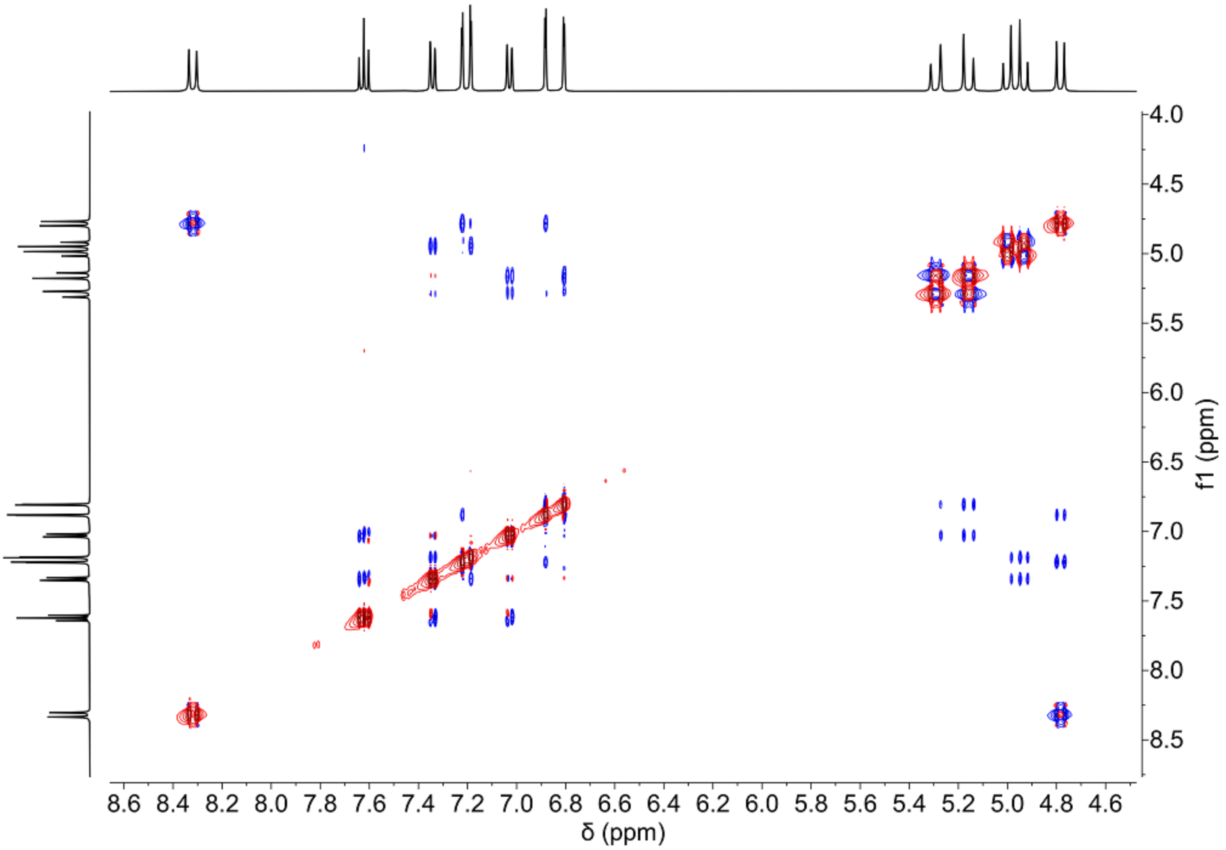

Figure S11. ^{1}H-^{1}H NOESY spectrum of [L_2Cu_4S](PF_6)$_2$ (**2**) in d_3-MeCN (400, 400 MHz, 298 K).

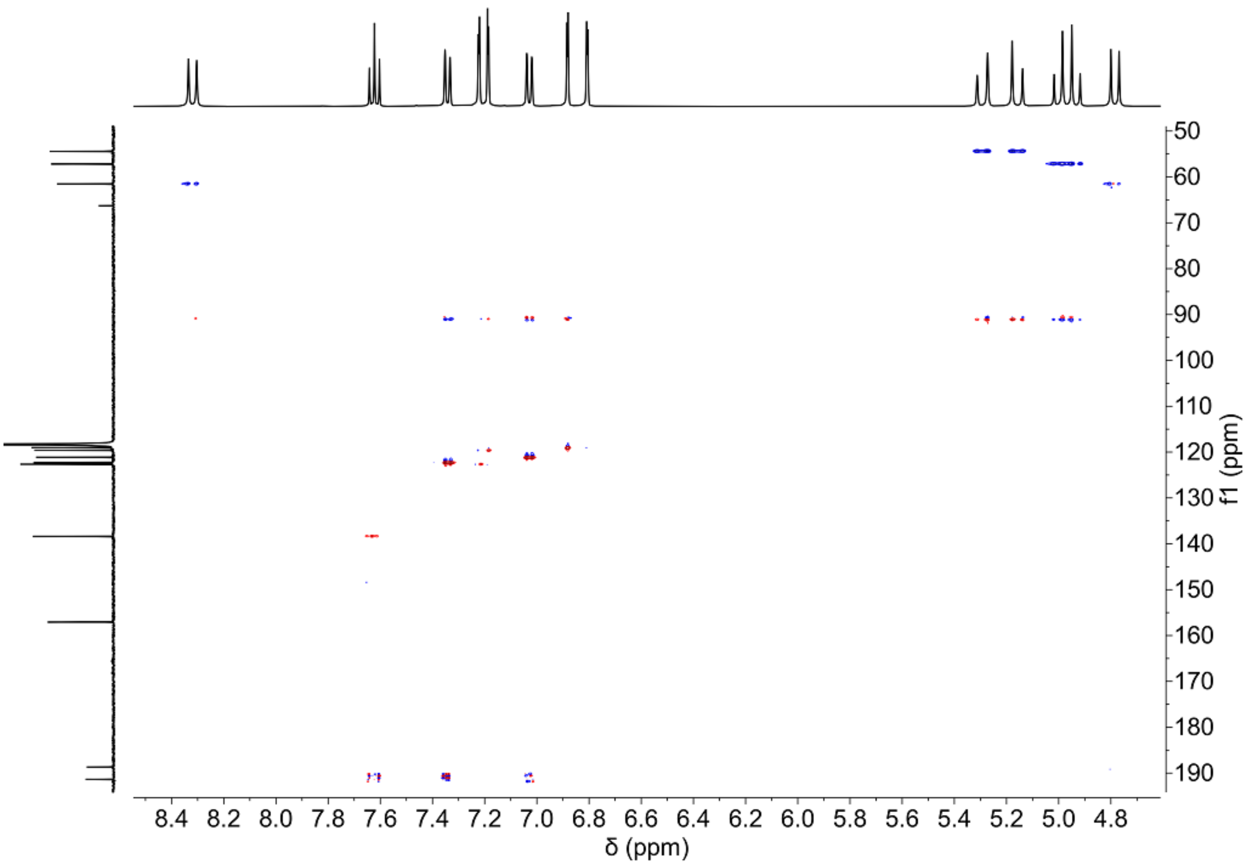

Figure S12. ^{1}H-^{13}C HSQC spectrum of $[L_2Cu_4S](PF_6)_2$ (**2**) in d_3-MeCN (400, 100 MHz, 298 K).

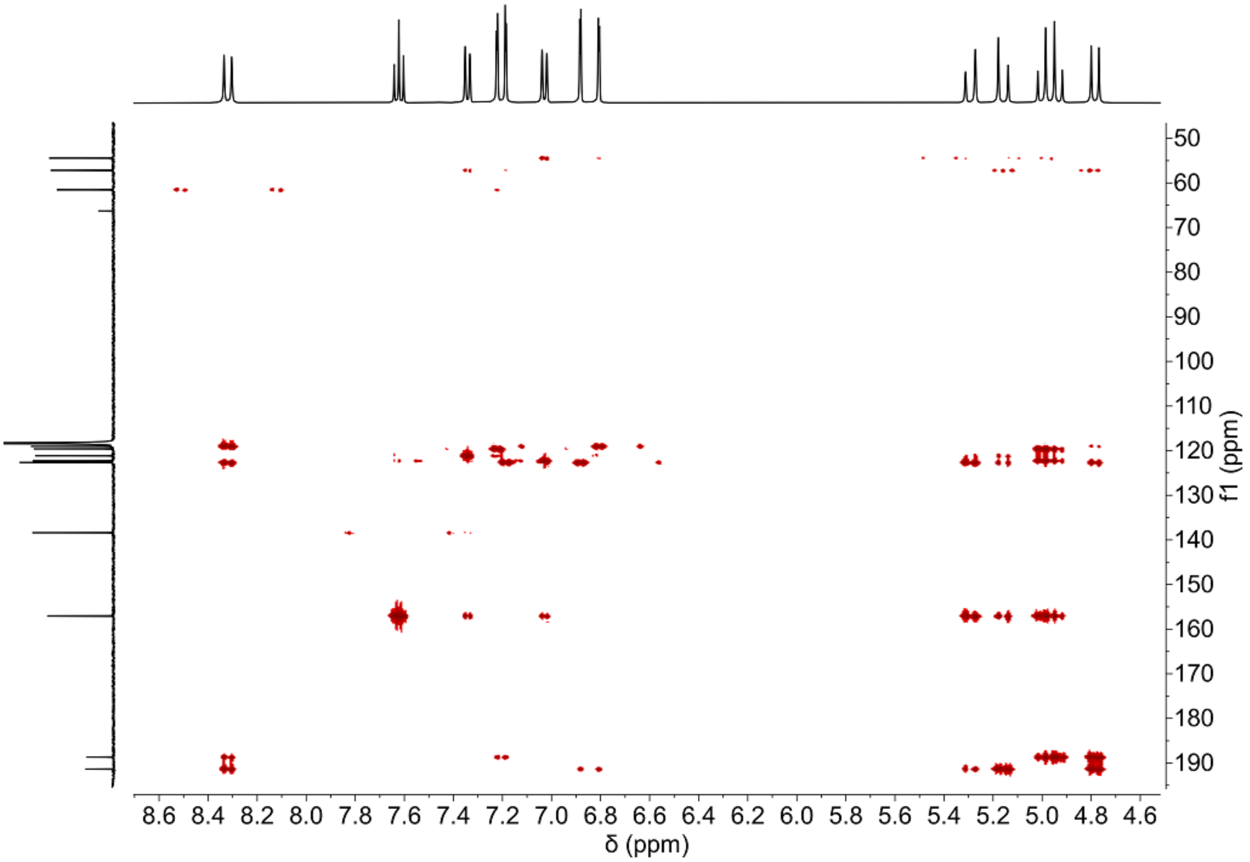

Figure S13. ^{1}H-^{13}C HMBC spectrum of $[L_2Cu_4S](PF_6)_2$ (**2**) in d_3-MeCN (400, 100 MHz, 298 K).

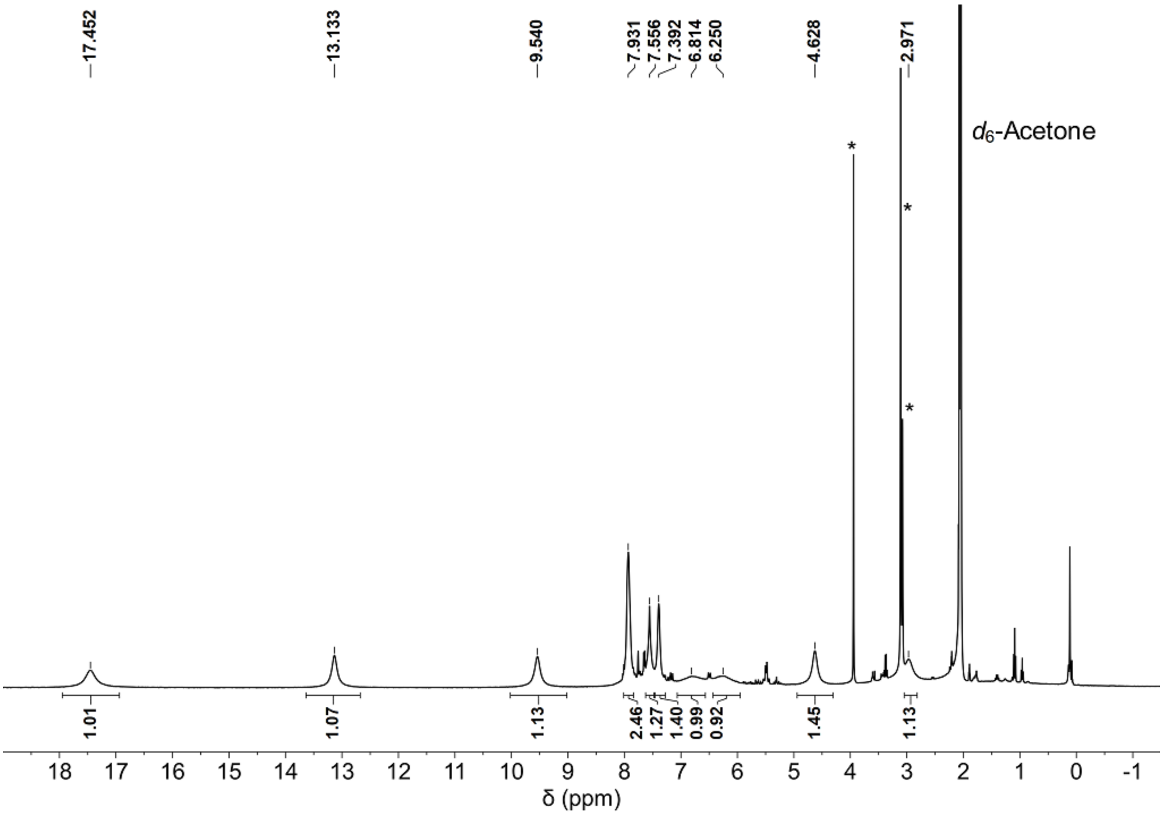

Figure S14. ^{1}H NMR spectrum of **3** prepared by in-situ oxidation of **2** with $[Cp^*_2Fe]PF_6$ in d_6-Acetone at 193 K (400K, 263 K). *Unknown species.

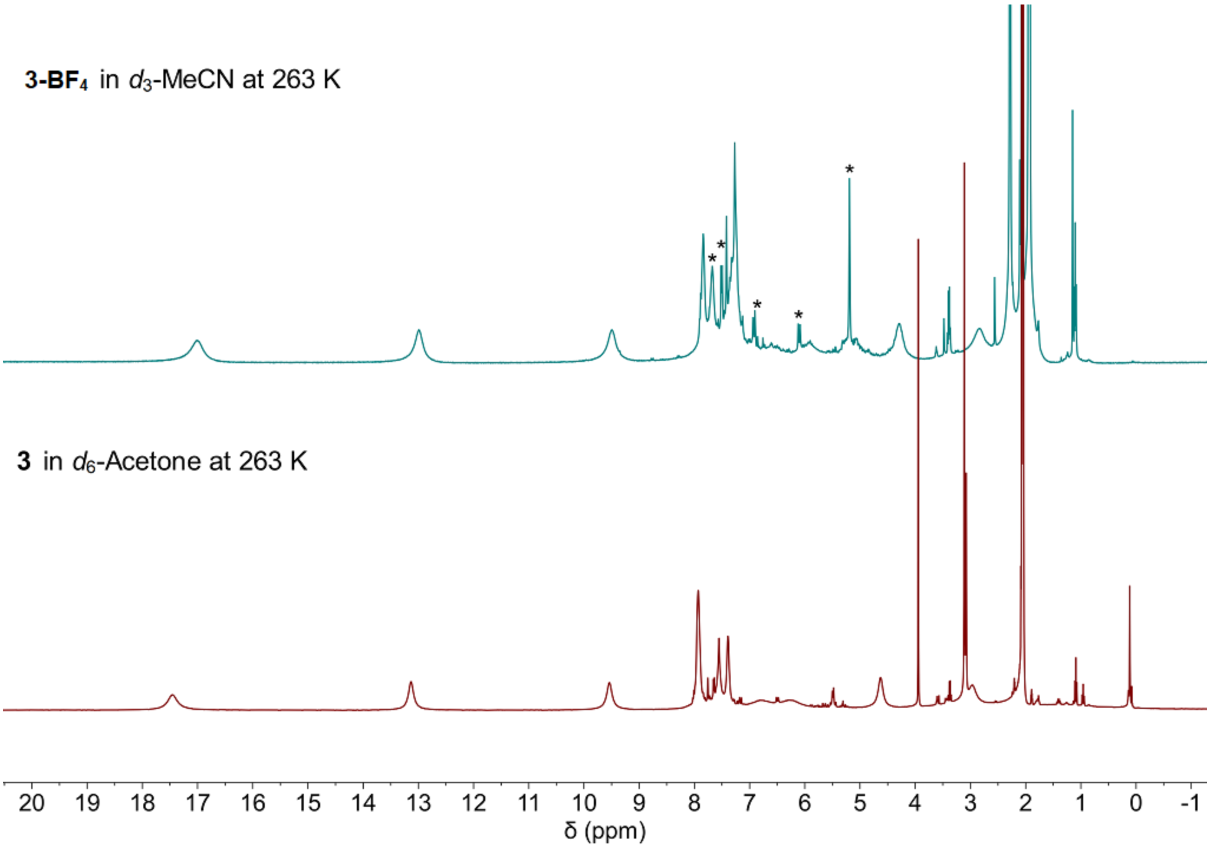

Figure S15. A comparison of the ^{1}H NMR spectra of **3-BF₄** in d_3-MeCN and **3** in d_6-acetone (400K, 263 K). Signals due to $[LCu_2](PF_6)_2$ (**1,** labeled with *) were observed because of the partial decomposition of **3/3-BF₄**.

Variable-Temperature ¹H NMR spectra

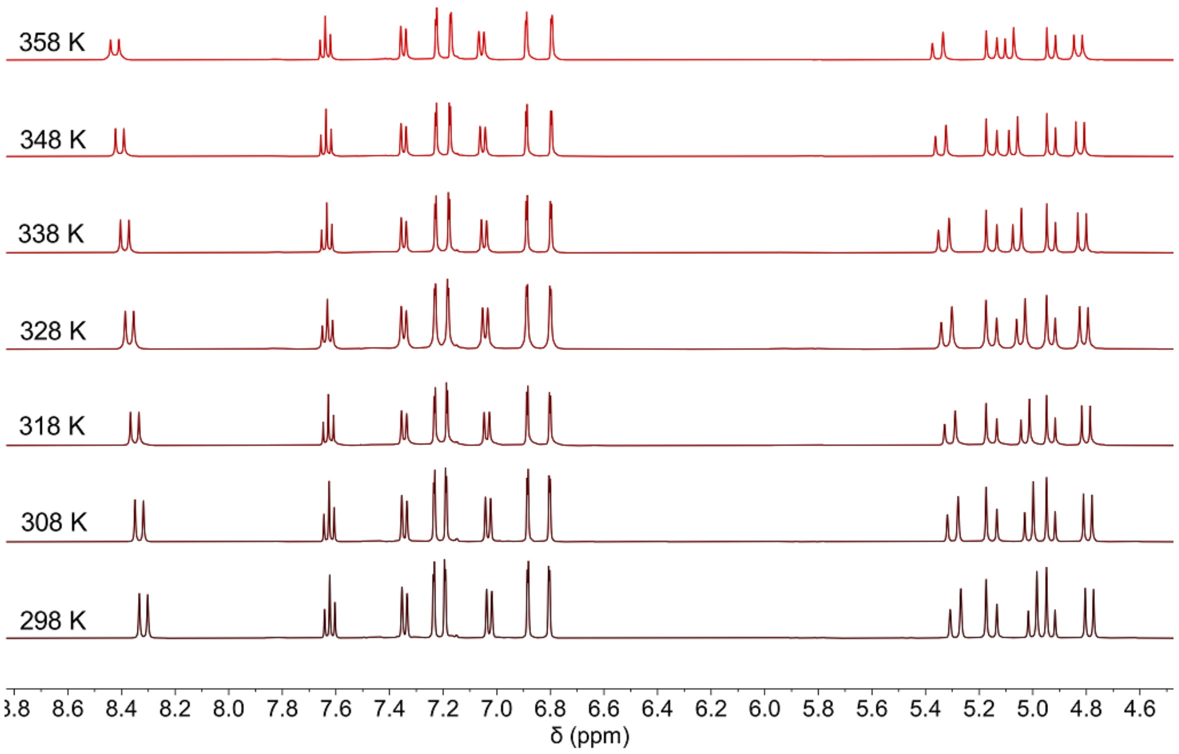

Figure S16. Variable-temperature (VT) ¹H NMR spectra (4.5–8.8 ppm) of $[L_2Cu_4S](PF_6)_2$ (**2**) in *d*3-MeCN (400 MHz, temperature range from 298 K to 358 K).

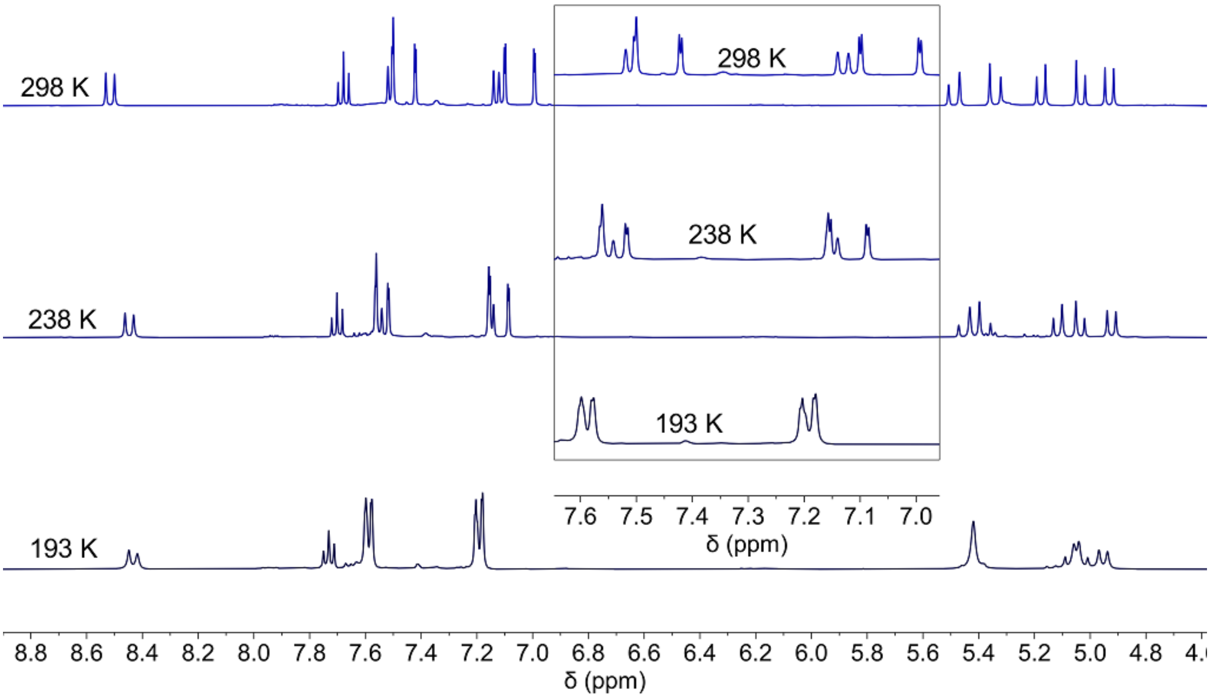

Figure S17. Variable-temperature (VT) ¹H NMR spectra (4.5–8.8 ppm) of $[L_2Cu_4S](PF_6)_2$ (**2**) in *d*3-Acetone (400 MHz, temperature range from 298 K to 193 K). Insert shows the signal at range of 6.9–7.7 ppm. The signals shift along with the decrease of the temperature, but no broadening was observed, indicating no conformational change of the **2** in acetone.

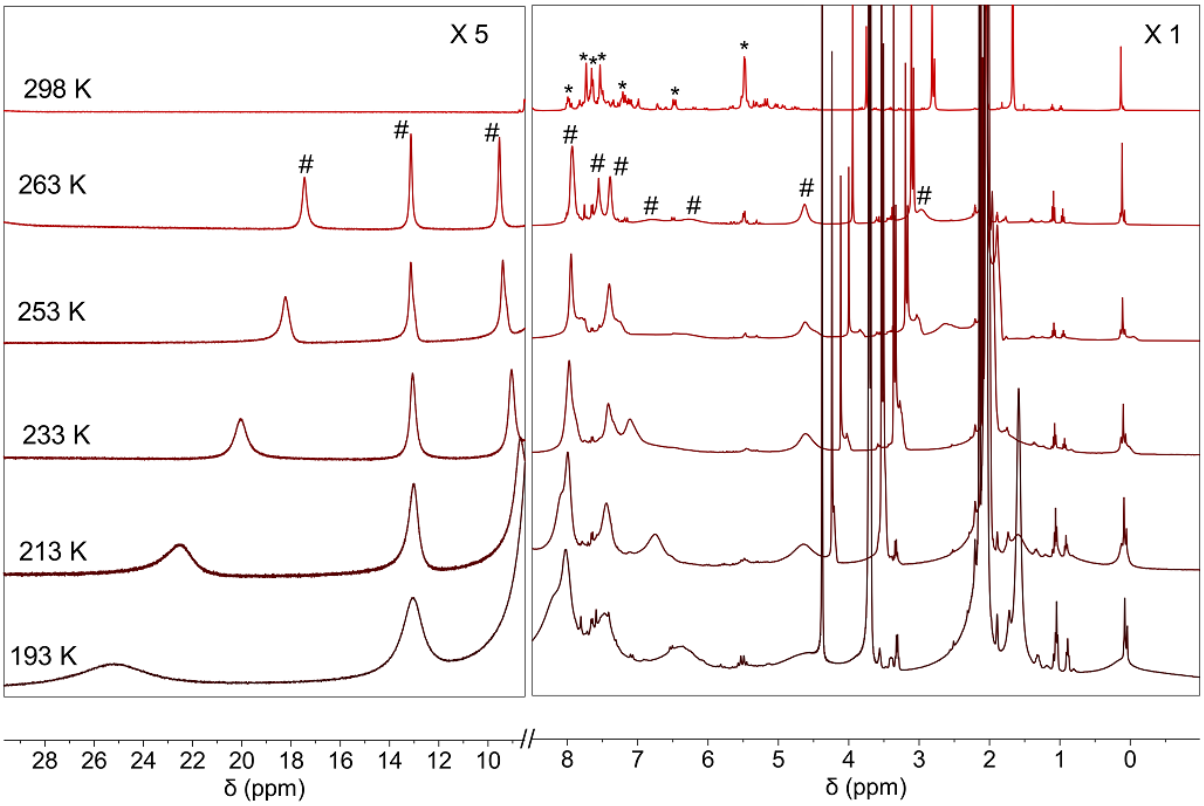

Figure S18. Variable-temperature (VT) ^{1}H NMR spectra (400 MHz) of $[L_2Cu_4S](PF_6)_3$ (**3**) prepared by in-situ oxidation of **2** with $[Cp^*_2Fe]PF_6$ in d_6-acetone at 193 K. The signal intensity in the range from 8.5 to 30 ppm (left) was scaled up by a factor of 5. The paramagnetic signals assigned to **3** (labeled with # in the spectrum at 263 K) disappeared at 298 K due to thermal decomposition of **3**, and the major decomposition product is $[LCu_2](PF_6)_2$ (**1**, labeled with *).

ESI Mass Spectrum of 2

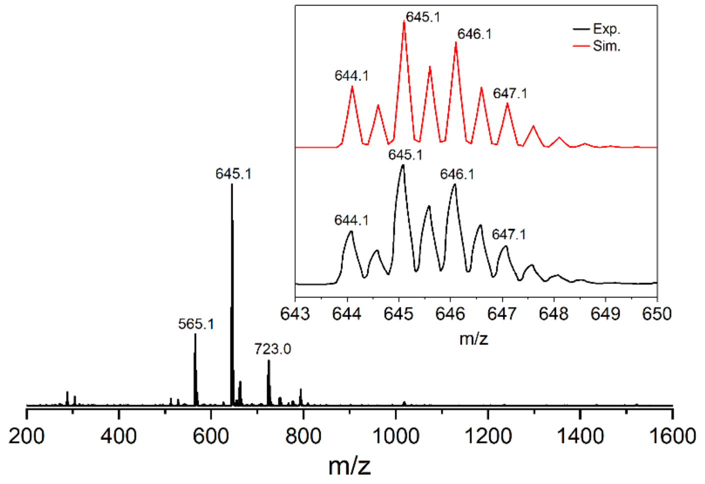

Figure S19. ESI mass spectrum (MeCN) of $[L_2Cu_4S](PF_6)_2$ (**2**). Insert shows the isotope pattern and a simulation for the peak around *m*/*z* = 645 $[M]^{2+}$. No peak for $[M-PF_6]^+$ (*m*/*z* = 1435) was observed.

IR Spectra of 2 and 3

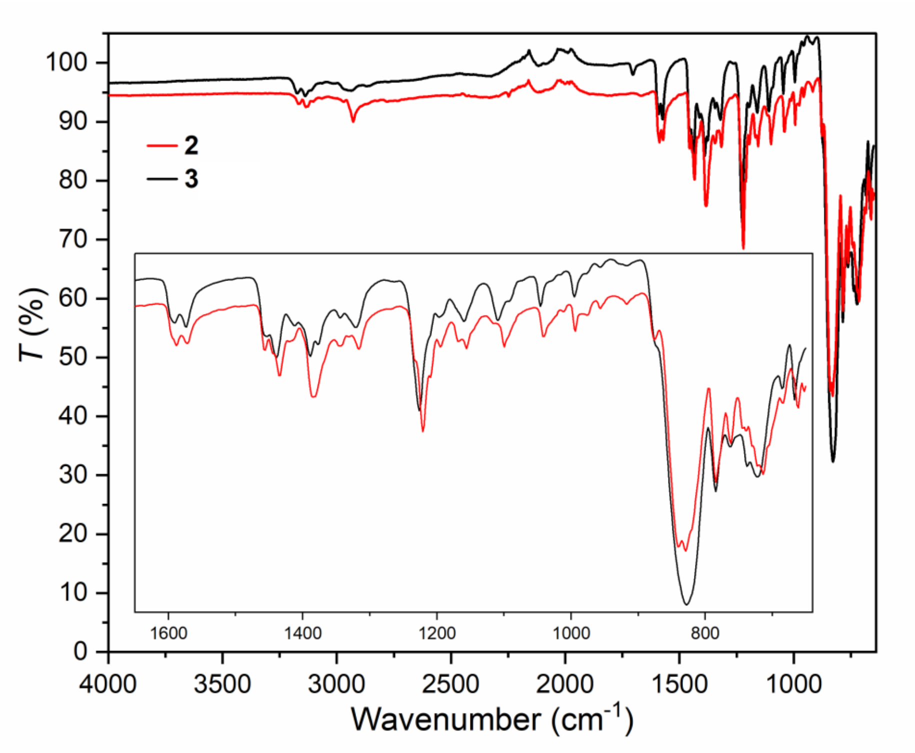

Figure S20. Comparison of ATR–IR spectra (solid, 650–4000 cm^{-1}) of **2** (red) and **3** (black). Insert shows the range from 650 to 1600 cm^{-1}.

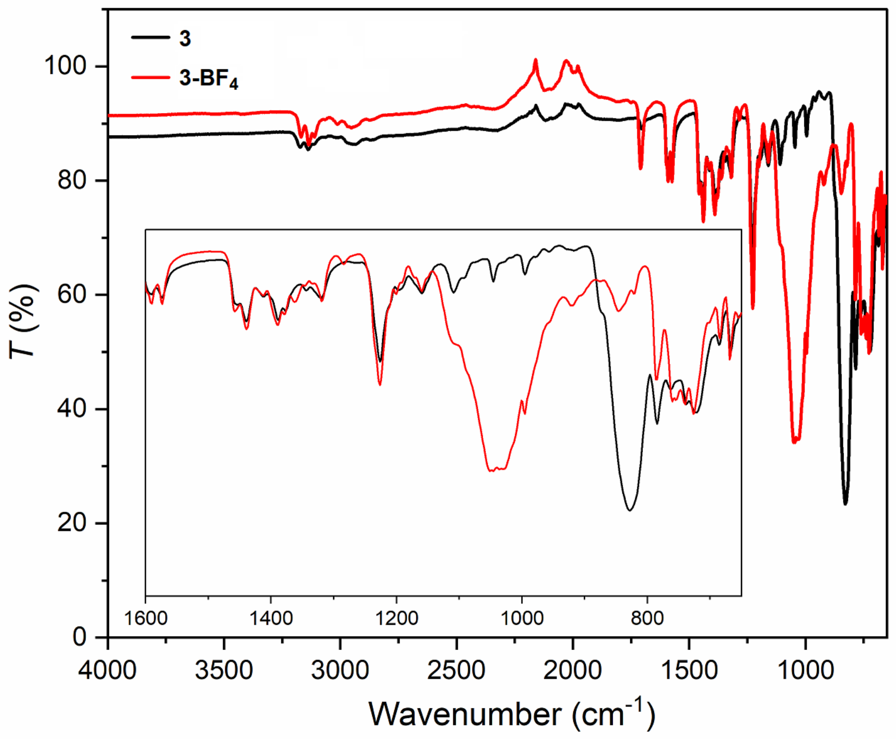

Figure S21. Comparison of ATR–IR spectra (solid, 650–4000 cm^{-1}) of **3** (black) and **3-BF$_4$** (red). Insert shows the range from 650 to 1600 cm^{-1}. The differences of the two spectra around 830 cm^{-1} and 1040 cm^{-1} are caused by the different counterions in **3** and **3-BF$_4$** (PF_6^- vs BF_4^-).

Magnetic Measurements

Temperature-dependent magnetic susceptibility measurements for **2** and **3** were carried out with a Quantum-Design MPMS-3 SQUID magnetometer equipped with a 7.0 T magnet in the range from 300 K to 2 K on a polycrystalline powdered sample under an applied magnetic field of 0.5 T. The crystalline solid sample was contained in a polycarbonate capsule and fixed in a nonmagnetic sample holder. The raw data file for the measured magnetic moment was corrected for the diamagnetic contribution of the capsule according to $M^{dia} = \chi_g \times m \times H$, with experimentally obtained gram susceptibilities of the capsule. The molar susceptibility data of the compounds were corrected for the diamagnetic contribution. Experimental data were modelled with the *julX* program[8] using a fitting procedure to the spin Hamiltonian: $\boldsymbol{\hat{H} = \mu_B g \vec{B} \cdot \vec{S}}$. Temperature-independent paramagnetism (TIP = 78·10^{-6} cm^3 mol^{-1} for **2** and 548·10^{-6} cm^3 mol^{-1} for **3**) was included according to $\chi_{calc} = \chi_{exp} +$ TIP. Effective magnetic moments were calculated according to $\mu_{eff} = \sqrt{\boldsymbol{7.997\chi_m T}}$.

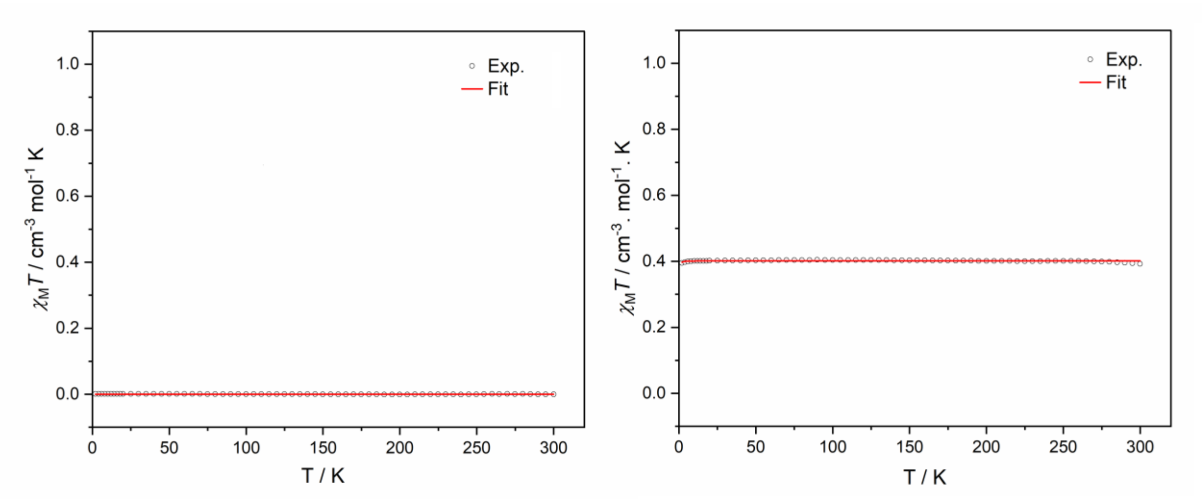

Figure S22. $\chi_M T$ vs T measurements for solid samples of (left) **2** and (right) **3** in the temperature range 300–2 K at 0.5 T. Best fitting parameters for the simulation of **3** (red solid line) with $S = ½$ are $g = 2.07$ and $TIP = 548 \cdot 10^{-6}$ cm^3 mol^{-1}.

EPR Spectroscopy

Continuous-wave X-band (~9.63 GHz) EPR spectra of **3** were measured on a Bruker Elexsys-E500 spectrometer equipped with an Oxford liquid helium flow cryostat. Spectra were collected in a dual-mode X-band resonator, operated in perpendicular mode (TE_{102}). All spectra were collected with 100 kHz field modulation at 6 G amplitude. All CW-EPR were simulated in Matlab 2022b with the EasySpin (v 6.0.0-dev49) package.[9] Q-band (34.0 GHz) Two-pulse (Hahn) echo detected EPR spectra were collected with a $\tau/2 - \pi - \tau -$ *echo* pulse sequence on a Bruker Elexsys-580 using a home-built up/down Q-band pulse conversion accessory and home-built TE_{011} microwave resonator. Cryogenic temperatures were maintained with an Oxford C-935 liquid helium cryostat.

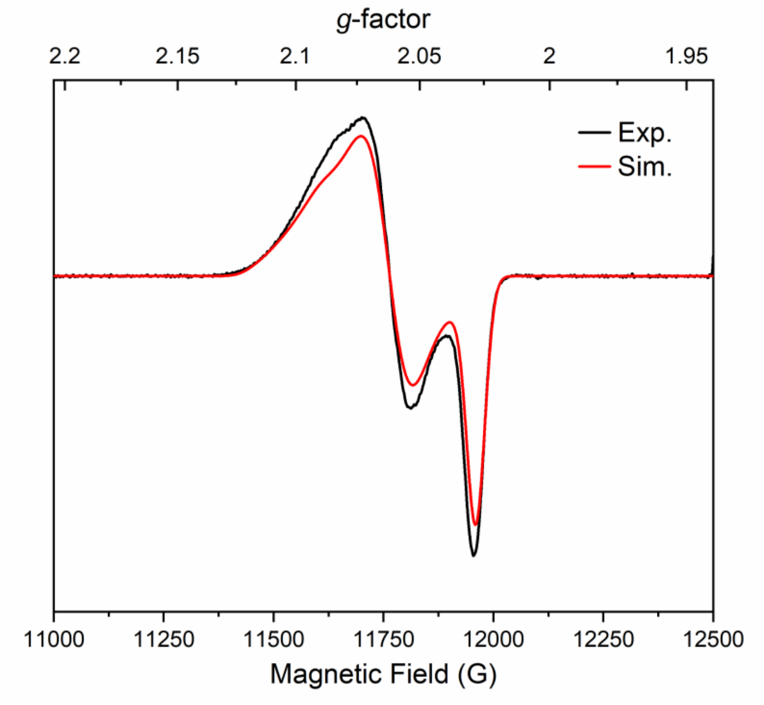

Figure S23. Numerical derivative of two-pulse echo-detected Q-band EPR spectrum of **3** at 15 K (black) and a simulation with the following parameters: g = [2.090, 2.064, 2.029], A(Cu) = [128, 44, 10] MHz for two equal Cu ions, linewidth 45 G (fwhm Gaussian), additional g-strain broadening of [0.01 0.01 0] (fwhm, Gaussian lineshape).

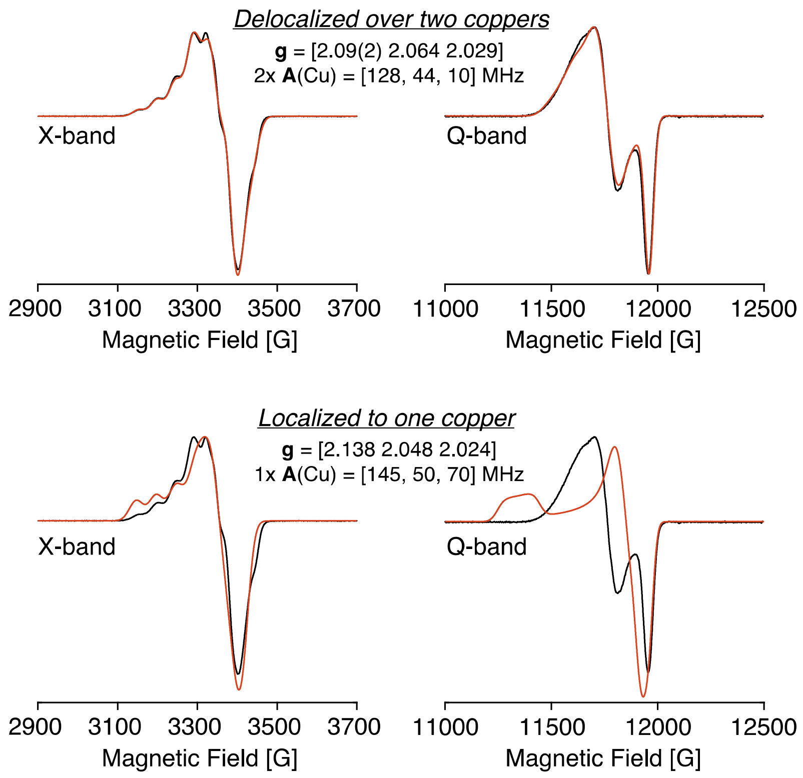

Figure S24. Simulations of X-band (9.63 GHz) and Q-band (34.0 GHz) EPR spectra of **3** where (top) the unpaired electron is either delocalized over two copper ions as represented by the inclusion of two equal copper hyperfine tensor interactions, or (bottom) the unpaired electron is localized on a single copper ion, represented by a single copper hyperfine tensor interaction. The simulation parameters for both X- and Q-band are detailed in the figure, X-band linewidth 34 G (fwhm), Q-band linewidth 45 G (fwhm).

Electrochemistry

Cyclic voltammetry (CV) experiments were performed at room temperature in a glovebox under an argon atmosphere with an Interface 1000B potentiostat. A common three electrode setup was used with a glassy carbon working electrode, a platinum wire as counter electrode, and a silver wire in [^{n}Bu$_4$N]PF$_6$ solution (I = 0.1 M) as pseudo reference electrode. 0.1 M [^{n}Bu$_4$N]PF$_6$ in MeCN was used as supporting electrolyte and was prepared in the glovebox. Ferrocene was used as an internal standard. The data were analyzed by Gamry Framework software.

The redox event at −0.65 V ($E_{1/2}$, vs Fc$^{+/0}$, 100 mV/s; Figure S25), assigned to the [L$_2$Cu$_4$(μ_4-S)]$^{3+/2+}$ redox couple, is reversible on the CV time scale as judged by the linear dependence of the currents I_f (forward peak) and I_r (return peak) on the square root of the scan rates (v$^{1/2}$) (Randles–Sevcik equation), the similar peak currents of forward and reverse scans (I_f/I_r = 0.86 at 100 mV/s), and the small peak-to-peak separation (76 mV at 100 mV/s).

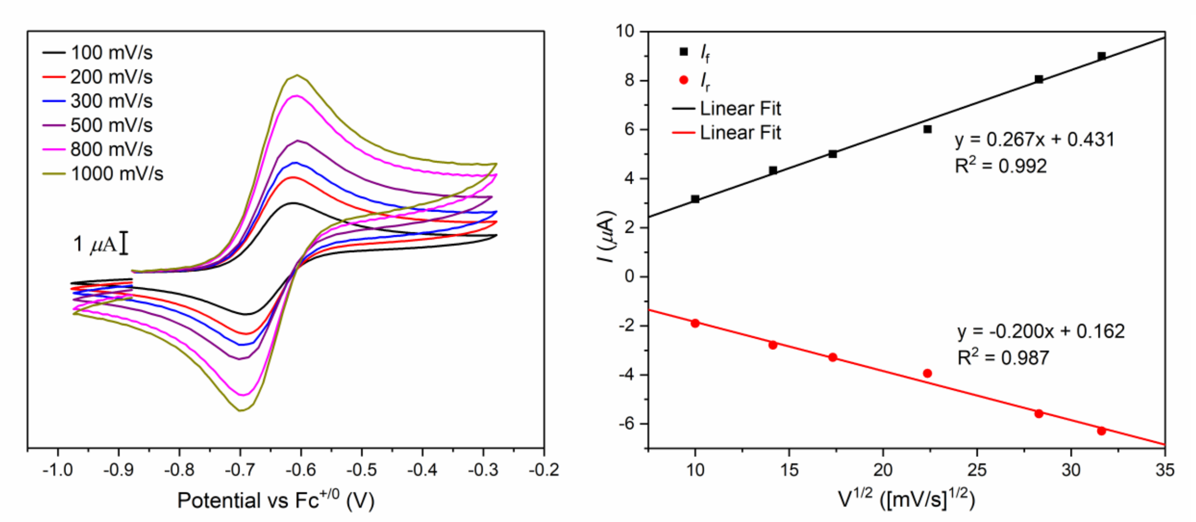

Figure S25. (Left) CV of **2** in MeCN at various scan rates with scan range from −0.5 to +1.3 V (vs Fc$^{+/0}$) and (right) the linear dependence of the currents I_f (forward peak) and I_r (return peak) on the square root of the scan rates (v$^{1/2}$).

UV-Vis-NIR spectroscopy

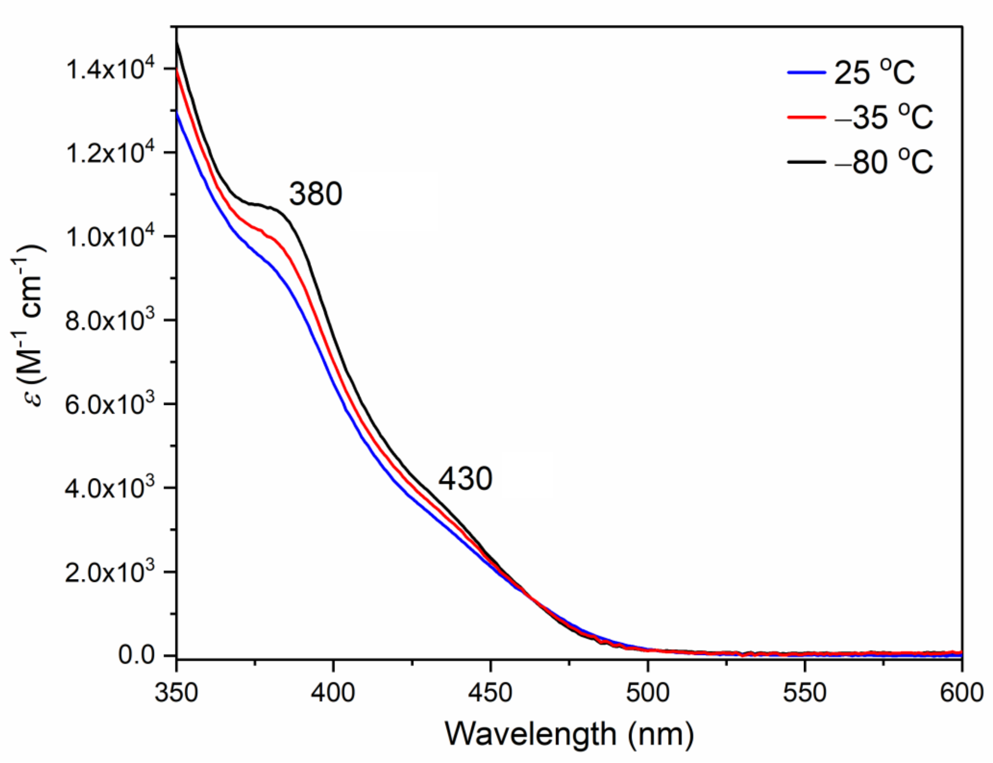

Figure S26. UV-vis spectra of **2** recorded in acetone at 25 °C (blue), −35 °C (red) and −80 °C (black). The positions of the absorption bands are labeled.

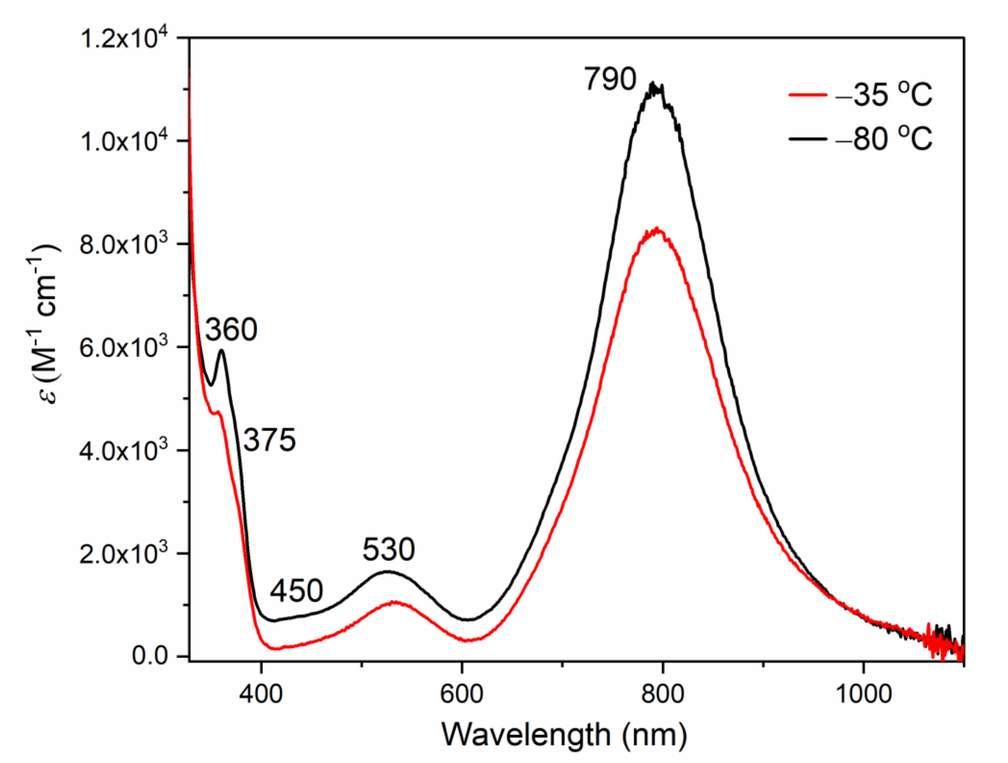

Figure S27. UV-vis-NIR spectra of **3** recorded in acetone at −35 °C (red) and −80 °C (black). The positions of the absorption bands are labeled.

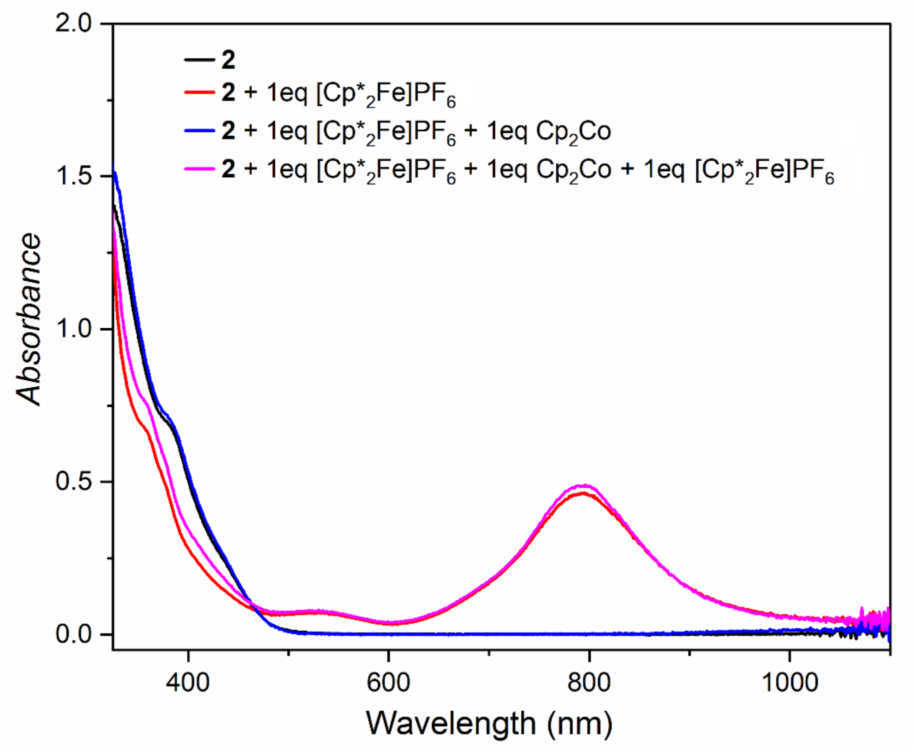

Figure S28. Redox interconversion between **2** (black) and **3** at −80 °C in acetone monitored by UV-vis-NIR spectra (red, the oxidation of **2** with $[Cp^*{}_2Fe]PF_6$; blue, the reduction of the in-situ formed **3** with Cp_2Co; pink, the re-oxidation of the formed **2** with $[Cp^*{}_2Fe]PF_6$.

DFT Calculations

Computational Details. All calculations were performed by using the ORCA quantum chemical program package (version 5.0.3).[10,11] Geometry optimizations (OPT) were performed with the B3LYP functional using the def2-TZVP basis set in combination with the auxiliary basis set def2/J,[12-16] and the CPCM (for TD-DFT) method has been applied to include solvent effects in the calculations.[17,18] The RIJCOSX approximations was used to accelerate the calculations.[19,20] The noncovalent interactions were considered via atom-pairwise dispersion corrections with Becke-Johnson (D3BJ) damping.[21] Coordinates from X-ray structural analyses (**2** and **3-BF$_4$**) were used as starting coordinates and the optimized coordinates of **2** and **3** is given in Table S9. TD-DFT calculations (80 roots) were carried out with the B3LYP functional using the same basis set combination.

Table S3. Comparison of the selected bond distances (Å) and bond angles (°) obtained from the optimized geometries (in MeCN) and experimental data of **1** and **3-BF$_4$**.

	**2**		**3-BF$_4$**	
	**Exp.**	**Opt.**	**Exp.**	**Opt.**
Cu–C	1.950(2)–1.991(2)	1.9848–2.0088	1.950(3)–1.972(4)	1.9819–1.9975
Cu–S	2.3021(6)–2.3499(5)	2.3640–2.3720	2.1982(9)–2.3215(9)	2.2472–2.3481
Cu···Cu	2.9718(5)–4.5867(4)	3.1658–4.6967	3.0952(6)–4.6374(7)	3.1943–4.6918
S···Cu$_4$^{a}	0.6359(6)	0.632	0.1476(8)	0.1476
C–Cu–C	117.20(8)–130.37(8)	124.17–134.54	129.99(15)–131.68(16)	130.94–134.03
Cu–S–Cu^b	134.71(3) / 160.80(3)	130.77 / 166.62	168.70(5) / 175.76(5)	169.22 / 176.01
τ_4(S)	0.46	0.44	0.11	0.10

^{a}distance of the sulfur atom to the plane of the four copper atoms. ^{b}Cu in opposite positions.

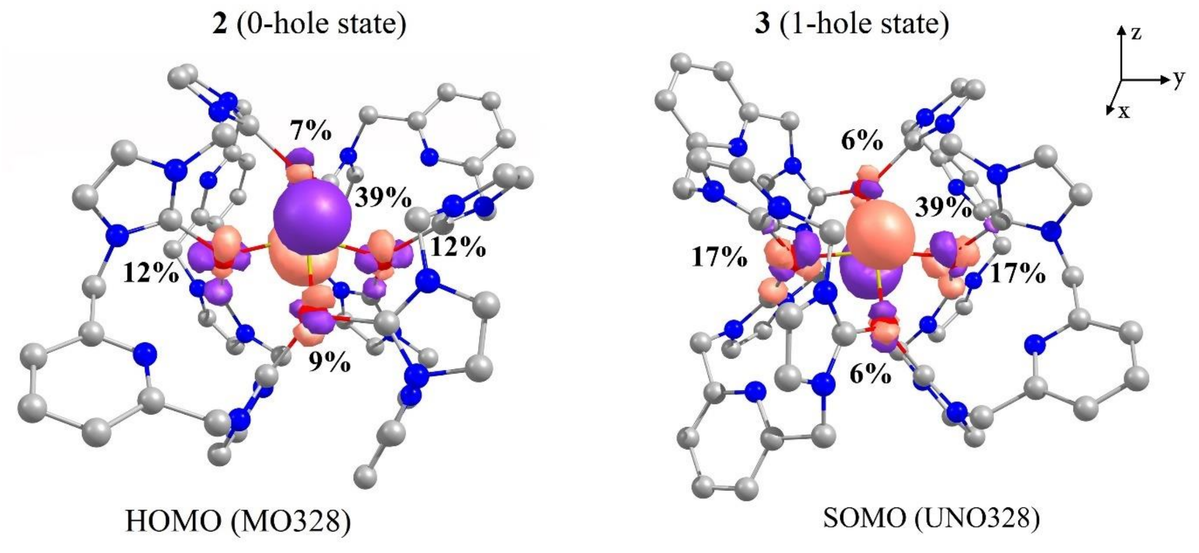

Figure S29. HOMO (MO328; left) and SOMO (UNO328; right) calculated for the cation of **2** (0-hole state) and **3-BF$_4$** (1-hole state), respectively; isosurface value = 0.05 au. The values of major orbital contributions are given.

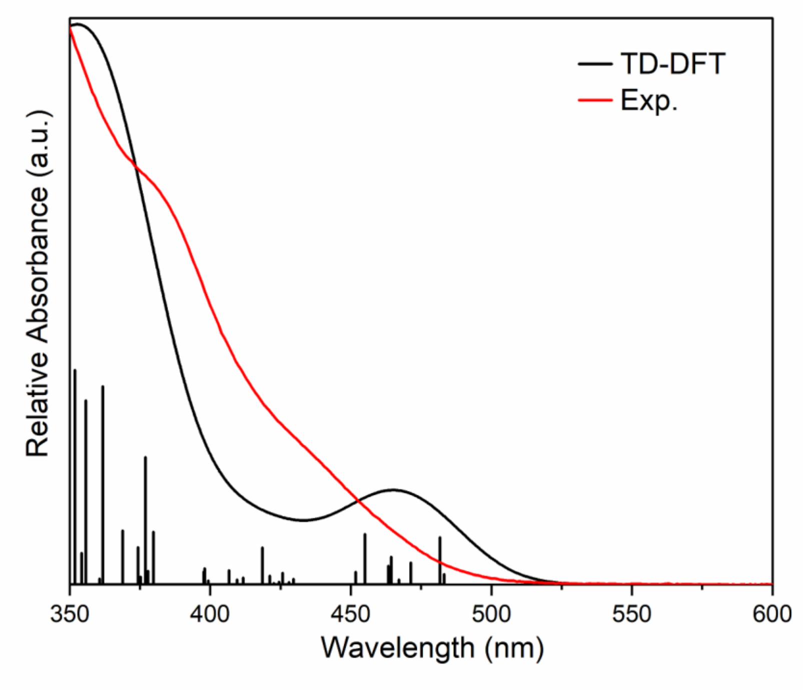

Figure S30. Comparison of experimental (in acetone, red) and TD-DFT calculated (in acetone, black) UV-Vis spectra for **2**. The calculated spectrum was convoluted using a Gaussian line shape function with a half-width of 40 nm. The principal contributions involved in the electronic transitions are described in Table S4 and Figure S31.

Table S4. Selected TD-DFT (B3LYP/TZVP level) calculated energies, oscillator strengths, and principal compositions of the electronic transitions of **2**.

States	Energy (cm^{-1})	Wavelength (nm)	Osc. Strength	Principal contributions
2	20759.9	481.7	0.014481500	327a → 329a (62%), 328a → 330a (14%)
7	21976.0	455.0	0.015513207	328a → 332a (94%)
15	23895.0	418.5	0.011321548	326a → 329a (63%), 326a → 331a (22%)
25	26532.2	376.9	0.039185816	326a → 335a (58%), 326a → 337a (23%), 326a → 331a (11%)
29	27645.5	361.7	0.060978655	327a → 339a (91%)
31	28115.6	355.7	0.056580275	328a → 340a (71%), 326a → 335a (9%)

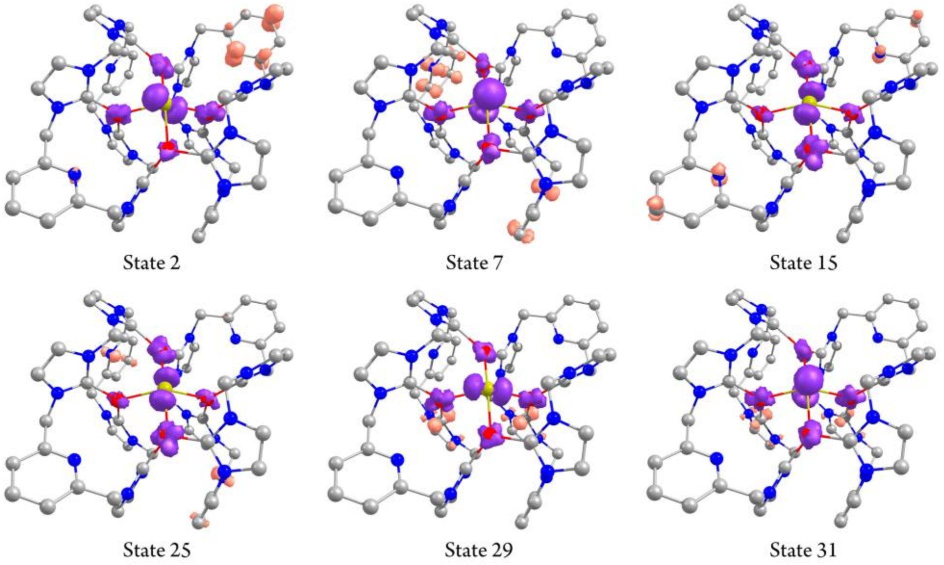

Figure S31. Plot of the TD-DFT difference densities for the electronic excitations (Table S4) calculated for the cation of **3-BF$_4$** (isosurface value = 0.006 au). Purple: negative densities; Orange: positive densities.

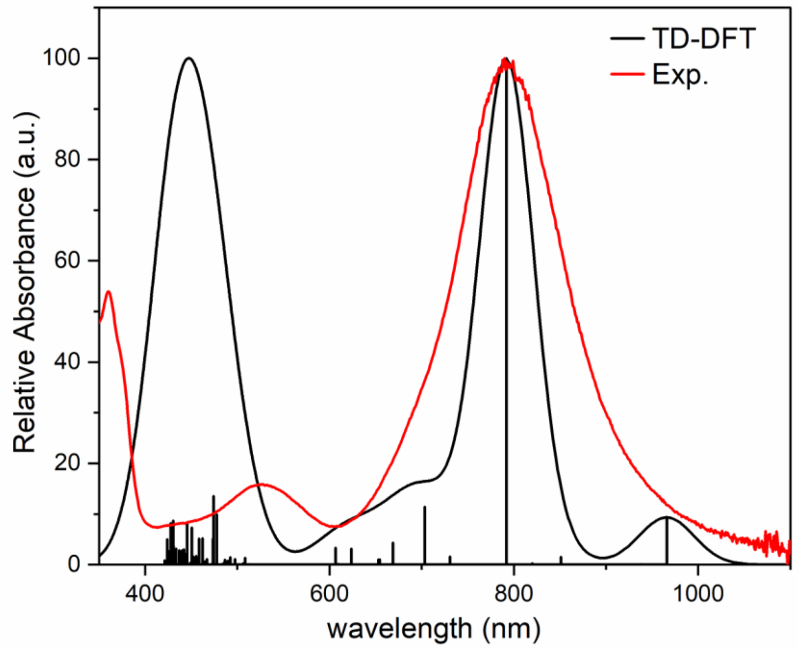

Figure S32. Comparison of experimental (in acetone, red) and TD-DFT calculated (in acetone, black) UV-vis-NIR spectra for **3-BF$_4$**. The calculated spectrum was convoluted using a Gaussian line shape function with a half-width of 70 nm and a shift of +110 nm was applied. The principal contributions involved in the electronic transitions are described in Table S5 and Figure S33.

Table S5. Selected TD-DFT (B3LYP/TZVP level) calculated energies, oscillator strengths, and principal compositions of the electronic transitions of **3-BF$_4$**.

States	Energy (cm^{-1})	Wavelength (nm)	Osc. Strength	Principal contributions
3	11683.9	855.9	0.018854447	323b → 328b (82%), 317b → 328b (5%)
7	14665.7	681.9	0.201178659	324b → 328b (87%)
9	16855.3	593.3	0.022993807	317b → 328b (45%), 315b → 328b (20%)
11	17898.5	558.7	0.008714820	315b → 328b (48%), 293b → 328b (18%), 317b → 328b (11%)
16	19463.0	513.8	0.006317925	311b → 328b (49%), 306b → 328b (22%), 300b → 328b (10%)
18	20132.7	496.7	0.006652829	308b → 328b (68%), 317b → 328b (9%)
31	27187.3	371.8	0.021158971	328a → 330a (49%), 328a → 333a (19%), 327b → 330b (13%)
32	27152.9	367.8	0.019887040	327a → 330a (51%), 327a → 333a (20%), 326b → 330b (12%)
33	27500.0	363.6	0.010279890	327b → 329b (93%)
42	28395.4	352.2	0.010403957	328a → 329a (89%)

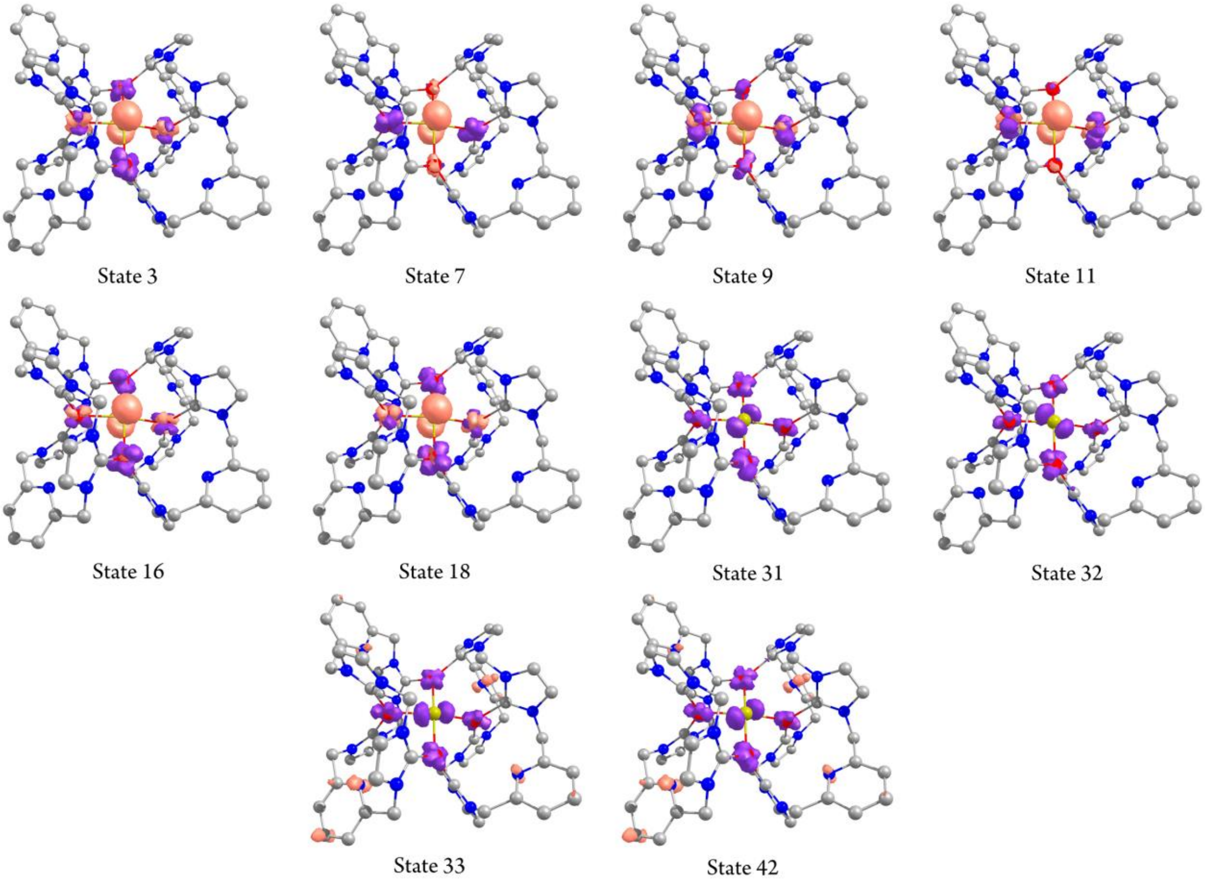

Figure S33. Plot of the TD-DFT difference densities for the electronic excitations (Table S5) calculated for the cation of **3-BF$_4$** (isosurface value = 0.008 au). Purple: negative densities; Orange: positive densities.

XES and XAS Data and Spectral Calculations

Sample Preparation for XAS and XES Measurements. For XAS experiments, solid samples were diluted with boron nitride (BN) to achieve a 2% (w/w) concentration of copper, then packed into 1 mm thick aluminum sample cells and sealed with 13 μm Kapton tape. For XES experiments, all samples were measured in the solid state. For Cu XES, the pure solids were ground to a fine powder and packed into 0.5 mm thick aluminum sample holders and sealed with 13 μm Kapton tape. For S XES, the pure solids were ground to a fine powder and packed into 0.5 mm thick aluminum sample holders and sealed with polypropylene.

Cu K-edge XAS. Cu K-edge X-ray absorption data were measured at the SAMBA beamline at Synchrotron SOLEIL (500 mA beam current). A double-crystal monochromator equipped with Si(220) crystals was utilized for upstream energy selection, providing an unattenuated flux of ~2.8×10^{11} photons/sec (ph/s) at the sample position. The X-ray beam was focused to an approximate beam spot size of 0.5×1.5 mm^2 (v × h). Samples were measured as solids diluted in BN, sealed with Kapton tape, followed by freezing and storage under liquid nitrogen until measurement. During measurement, sample temperature was maintained at 10 K to minimize photodamage.

Prior to collection of full spectra, a series of short, low-resolution scans at the near-edge region were collected to identify X-ray-induced photodamage. All data were collected by scanning the incident energy from 8829 to 9970 eV, and calibrated by simultaneous measurement of a Cu foil, for which the first inflection point of the Cu foil was set to 8980.3 eV. Three ionization chambers were positioned before the sample (I_0), after the sample (I_t), and after a reference foil (I_{ref}), and fluorescence data from the sample was recorded by a 35-element monolithic planar Ge pixel array detector.

Cu and S VtC XES Measurements. The Cu and S valence-to-core X-Ray Emission Spectroscopy (VtC XES) data collection was performed at the PINK tender X-ray beamline at BESSY II. A considerable gain in intensity of the photon beam was obtained by using a multilayer monochromator (≈ 80–100 eV band pass). The beam size on the sample was 30×500 μm^2 (v × h). All spectra were collected using two in-house designed energy dispersive von Hamos spectrometers. The analyzers were set up in a vertical dispersion direction, taking advantage of the small vertical beam size to improve the energy resolution. The sample environment was fixed at 22 K in a cryo-chamber, using Helium as the exchange gas, at a pressure of 8 mbar. The entrance window was made from 1μm graphenic carbon and for the exit window, a cold window of 8 μm Kapton was used and the window to the spectrometers were 13 μm Kapton.

For Cu Kβ XES measurements, a Si(444) 1 mm diced crystal with a bending radius of R = 247 mm dispersed incoming fluorescence radiation onto an Eiger detector with a 75 μm × 75 μm pixel size. The CCD detector accepted fluorescent radiation reflected from the crystal analyzer under 60.7°–68° Bragg's angles that corresponded to an energy window of 8535–9075 eV (spanning the Kβ mainline and valence-to-core regions). The spectrometer resolution was about 1 eV. The excitation energy was 9500 eV and incoming photon flux ≈ 8×10^{12} ph/s using atmospheric von Hamos spectrometer. Cu Kβ XES damage scans and assessments were performed for all samples. In all cases, the data were collected with continuous sample motion. Samples were scanned at a rate of 100 μm/s, resulting in a total exposure time of 0.50 s/pass and the total exposure was 20 × 0.5 = 10 s per sample, with a total measurement time of 2 h. None of the samples exhibited damage or changes in the Kβ emission spectra within the measurement scan periods. XES data were processed and the integrated

intensity of the Kβ main line was set to 1.0. For the energy calibration procedure Cu, Zn and Ho foils were used. The energy points used for the energy calibration were: Cu Kβ: 8905.42, Zn: Kα_1 8638.86, Kα_2 8615.78, Ho Lα_1: 6719.8 eV (X-ray data booklet). The Ho Lα spectrum was collected from Si(333) reflection of the same crystal without any rearrangements of the photon beam or the spectrometer. To define the peak positions Zn K$\alpha_{1,2}$ and Cu Kβ were fitted with split Voigt function. Ho Lα_1 line position was set to maximum of intensity. Energies was translated into Bragg angles and a fit with tangential function was applied.

The S-Kβ XES spectra were collected using a Si(111) crystal with a bending radius of R = 247 mm dispersed incoming fluorescence radiation onto a CCD detector with a 26 μm × 26 μm pixel size. The CCD detector accepted fluorescent radiation reflected from the crystal analyzer under 52°–54° Bragg's angles that corresponded to an energy window of 2436–2500 eV. In the current configuration, the detector resolution was 0.06 eV/pix. The spectrometer resolution was about 0.5 eV. The excitation energy was 4000 eV and incoming photon flux was ≈ 3 × 10^{13} ph/s in the vacuum von Hamos spectrometer. Measurements were done using zigzag mode, when samples were irradiated from spot to spot over the sample area (moment dose 1s at spot, 4 passes, total dose 4s). This was replicated three times at different sample areas. Total measurement time for each sample was ≈ 3 × 15 min = 45 min. None of the samples exhibited damage or changes in VtC XES spectra the measurement scan periods. XES data were processed and the integrated intensity of the Kβ main line was set to 1.0. Calibration of the energy scale was done with three reference samples: Na_2SO_3 and Na_2SO_4 powders, and Ni foil. Ni K$\alpha_{1,2}$ emission spectrum was collected at higher excitation energy of 9500 eV using Si(333) reflection of the same crystal. The reference emission spectra were fitted with 5 and 4 Voigt profiles, and 2 asymmetric Lorentzian profiles, respectively. The reference energies are presented in Table S6. For the energy calibration we converted energies of the tabulated peaks to Bragg angles and fit positions of the picked peaks with a tangential function.

Table S6. Reference energies (eV) used for the S-Kβ XES energy calibration[22,23]

	Kβ'	K$\beta_{1,3}$	Kβ_{XX}	Kα_1	Kα_2
Na_2SO_3	2451.84	2465.83	2472.31		
Na_2SO_4	2452.91	2467.15			
Ni foil				7478.26	7461.04

Computational details. All XES and XAS spectra were calculated within the ORCA code utilizing previously published protocols.[24,25] All DFT and TDDFT were performed with the ORCA electronic structure package 5.0.3.[10,11] Geometry optimization calculations were carried out at the B3LYP[12,13] level of theory, using def2-variants of Ahlrichs' allelectron Gaussian triple-ζ valence polarized recontracted basis set (def2-TZVP)[15] on all atoms and the AutoAux basis option for ORCA.[26] The calculations employed the resolution of identity (RI-J) algorithm for the computation of the Coulomb terms and the 'chain of spheres exchange' (COSX) algorithm for the calculation of the exchange terms[20] and a tight self-consistent field (SCF) convergence threshold was chosen via the "TightSCF" keyword. Defgrid2 was used during the SCF iterations and for the final energy evaluation after SCF convergence. The conductor-like polarizable continuum model (CPCM) was used for charge compensation in all calculations of complexes carrying a net positive/negative charge.[17] Cu Kβ and S Kβ VtC XES were calculated using the features described above, with the exception that a scalar relativistic basis set (ZORA-def2-TZVP)[15] was employed. In the XES block, "CoreOrb" dictates the orbitals that will be the electron acceptors. All virtual orbitals

were chosen as potential acceptor orbitals. For transition metal XES spectra, the metal 1s orbital is usually orbital 0, and is thus selected. OrbOp defines the operators of the electrons ($\alpha = 0$, $\beta = 1$) that will be calculated, both of which were selected. CoreOrbSOC defines which core orbitals ($\alpha = 0$, $\beta = 1$) are treated with spin-orbit coupling (SOC), both of which were selected to accommodate the OrbOp selection. A 2.0 eV full-width half-max Gaussian broadening and −34.5 eV energy shift was applied when plotting all calculated Cu and S XES spectra.

Time-dependent DFT (TD-DFT) calculations used the B3LYP functional, ZORA-def2-TZVP basis set, AutoAux and the zeroth-order regular approximation (ZORA) to account for scalar relativistic effects.[27,28] TD-DFT calculated XAS were shifted by −14.4 eV and a 2.0 eV full-width half-max Gaussian broadening was applied when plotting all calculated XAS spectra.

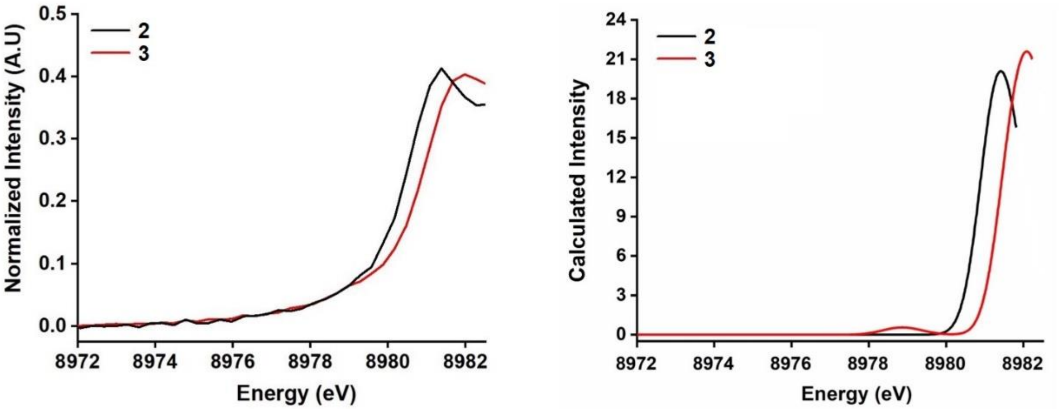

Figure S34. Experimental (left) and TD-DFT calculated (right, B3LYP/def2-TZVP) Cu K-edge XAS spectra for complexes **2** and **3**.

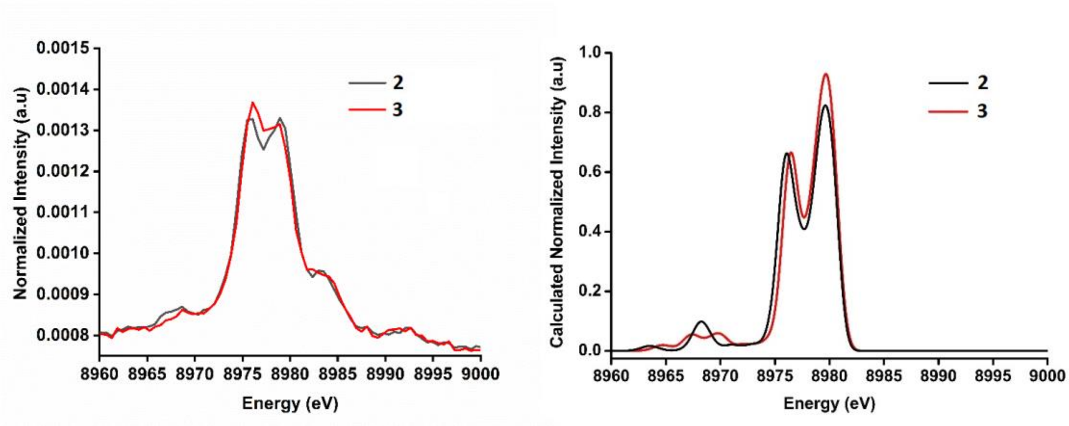

Figure S35. Experimental (left) and DFT calculated (right, B3LYP/def2-TZVP) Cu VtC Kβ XES spectra for complexes **2** and **3**.

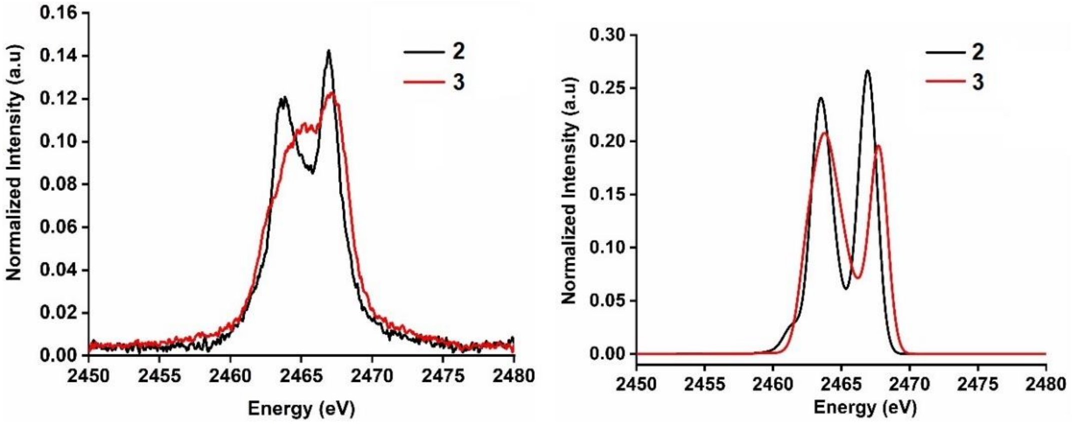

Figure S36. Experimental (left) and DFT calculated (right, B3LYP/def2-TZVP) S VtC Kβ XES spectra for complexes **2** and **3**.

Table S7. Analyses of Loewdin charge (e) and Loewdin spin density for **2** (0-hole state) and **3** (1-hole state) with respect to Cu and S at the B3LYP/def2-TZVP level.

Complex	Loewdin charge (e)					Loewdin spin density				
	Cu1	Cu2	Cu3	Cu4	S	Cu1	Cu2	Cu3	Cu4	S
**2** (0-hole)	−0.32	−0.32	−0.32	−0.32	0.46					
**3** (1-hole)	−0.28	−0.28	−0.28	−0.28	0.66	0.05	0.19	0.05	0.18	0.41

Table S8. Molecular orbital (MO) analyses for **2** (0-hole state) and **3** (1-hole state) at the B3LYP/def2-TZVP level.

Complex	% Cu in HOMO	% Cu in LUMO	% S in HOMO	% S in LUMO	Δ (HOMO to LUMO)
**2** (0-hole)	45.3%	2.4%	40%	0.6%	2.98 eV
**3** (1-hole)	59%	49%	18%	35%	1.81 eV

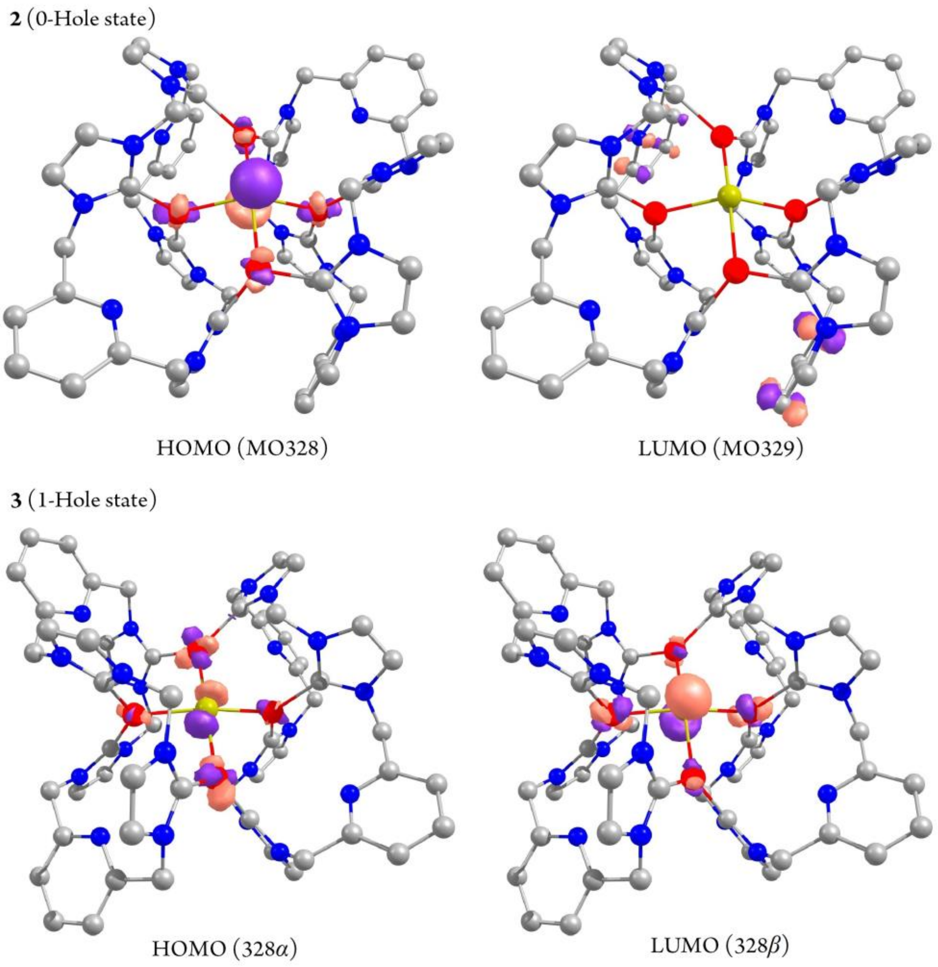

Figure S37. The HOMO and LUMO calculated (B3LYP/def2-TZVP) for **2** (0-hole state) and **3** (1-hole state), isosurface value = 0.08 au.

Reactivity Studies of Complex [L_2Cu_4S](PF_6)$_2$ (2)

Reaction of 2 with N_2O

At room temperature, a d_3-MeCN (0.5 mL) solution of [L_2Cu_4S](PF_6)$_2$ (**2**, 5.0 mg, 3.2 μmol) was prepared in a N_2-filled glovebox, and then transferred into a *J. Young* NMR Tube. The NMR tube was connected to the Schlenk line and an N_2O cylinder with a T-junction. After three freeze-vacuum-thaw cycles, 1 atm of N_2O was injected into the NMR tube at room temperature. No color change was observed, and ^{1}H NMR spectra collected at both room temperature (298 K) and low temperature (238 K) are identical to the spectra of **2** under an N_2 atmosphere at the same temperatures. The reaction was also tried in other less coordinating solvents, e.g., d_6-acetone and d_3-$MeNO_2$, and in the presence of the Cp_2Co as an electron donor, but still, no change of the ^{1}H NMR spectra was observed. Those results indicate that **2** is inert to N_2O at the conditions tested.

Reaction of 2 with [^{n}Bu$_4$N]N_3 and [^{n}Bu$_4$N]NO_2

At room temperature, to a solution of [L_2Cu_4S](PF_6)$_2$ (**2**, 10.0 mg, 6.3 μmol) in MeCN (2 mL) was added ^{n}Bu$_4$N]N_3 (5.3 mg, 18.9 μmol) or [^{n}Bu$_4$N]NO_2 (5.5 mg, 18.9 μmol). The reaction mixture was stirred at room temperature overnight, and the color of the solution remained yellow during that time. After filtration, the resulting solution was left for crystallization through Et_2O diffusion at room temperature. In both reactions, a large amount of yellow crystals was obtained which have the same unit cell as **2**, as confirmed by XRD. ^{1}H, ^{31}P and ^{19}F NMR spectra of these crystals are identical with those of **2** indicating that no reaction of **2** with [^{n}Bu$_4$N]N_3 or [^{n}Bu$_4$N]NO_2 took place.

Reaction of 2 with KI

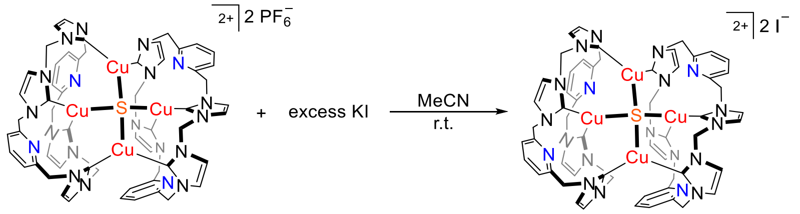

2 **4**

At room temperature, to a solution of [L_2Cu_4S](PF_6)$_2$ (**2**, 10.0 mg, 6.3 μmol) in MeCN (2 mL) was added KI (3.1 mg, 18.9 μmol). The reaction mixture was stirred at room temperature overnight, and the color of the solution remained yellow during that time. After filtration, the resulting solution was left for crystallization through Et_2O diffusion at room temperature, and yellow crystals of [L_2Cu_4S]I_2 (**4**) were obtained (92%, 8.9 mg). The replacement of counterion PF_6^- by I^- was confirmed by X-ray diffraction (Figure S38), and by ^{31}P and ^{19}F NMR analyses where no ^{13}P and ^{19}F resonances were observed. The ^{1}H and ^{13}C NMR as well as UV-vis spectra of **4** are nearly identical to those of **2** (Figures S39 and S40). ^{1}H NMR (400 MHz, d_3-MeCN, 298 K): δ (ppm) 8.31 (d, J = 12.4 Hz, 4H, NHC–CH_2–NHC), 7.63 (t, J = 7.6 Hz, 4H, CH–p–Py), 7.35 (d, J = 7.2 Hz, 4H, CH–m–Py), 7.27 (d, J = 1.6 Hz, 4H, CH-imidazolyl backbone), 7.21 (d, J = 1.6 Hz, 4H, CH-imidazolyl backbone), 7.03 (d, J = 7.6 Hz, 4H, CH–m–Py), 6.90 (d, J = 1.6 Hz, 4H, CH-imidazolyl backbone), 6.81 (d, J = 2.0 Hz, 4H, CH-imidazolyl backbone), 5.29/5.16 (ABq, 8H, J = 16 Hz, Py–CH_2–NHC), 5.00/4.94 (ABq, 8H, J = 12.8 Hz, Py–CH_2–NHC), 4.80 (d, J = 12.4 Hz, 4H, NHC–CH_2–NHC). ^{13}C NMR (100 MHz, d_3-MeCN, 298 K): δ (ppm) 191.4/188.7 (NCN), 157.1/157.0 (CH-o-Py), 138.4 (CH–p–Py), 122.69/122.65 (CH-imidazolyl backbone), 122.3/121.1 (CH–m–Py), 119.6/119.0 (CH-imidazolyl

backbone), 61.5 (NHC–*C*H_2–NHC), 57.2/54.4 (Py–*C*H_2–NHC). Anal. Calcd. for $C_{56}H_{52}Cu_4N_{20}SI_2$: C 43.53, H 3.39, N 18.13, S 2.07; Found: C 43.13, H 3.60, N 18.10, S 1.72. ESI–MS spectrum of **4** shows the peak at m/z = 645.1 $[M]^{2+}$ with the identical isotope pattern with **2** (Figure S19). Absorption spectrum (MeCN, 298 K): λ_{max} = 320, 380 and 430 nm.

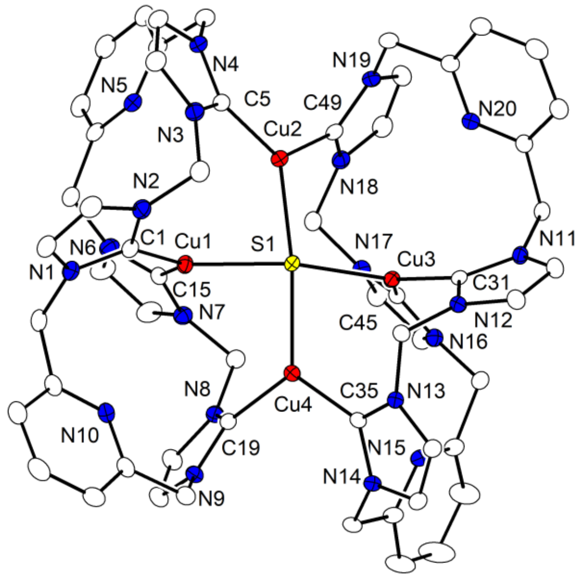

Figure S38. Plot (30% probability thermal ellipsoids, hydrogen atoms omitted) of the molecular structure of the cationic part of **4**. Selected bond lengths [Å] and angles [°]: Cu1–C15 1.965(3), Cu1–C1 1.987(3), Cu1–S1 2.3339(7), Cu2–C49 1.957(3), Cu2–C5 1.966(3), Cu2–S1 2.3385(8), Cu3–C45 1.967(3), Cu3–C31 1.979(3), Cu3–S1 2.3402(7), Cu4–C19 1.961(3), Cu4–C35 1.971(3), Cu4–S1 2.3173(7), Cu1···Cu2 2.9820(5), Cu1···Cu3 4.3715(5), Cu1···Cu4 3.2423(6), Cu2···Cu3 3.4710(6), Cu2···Cu4 4.5559(6), Cu3···Cu4 2.9943(5); C15–Cu1–C1 128.16(12), C15–Cu1–S1 121.39(8), C1–Cu1–S1 110.39(9), C49–Cu2–C5 125.27(13), C49–Cu2–S1 116.35(8), C5–Cu2–S1 118.13(10), C45–Cu3–C31 130.73(11), C45–Cu3–S1 119.34(8), C31–Cu3–S1 109.86(8), C19–Cu4–C35 118.93(12), C19–Cu4–S1 123.47(8), C35–Cu4–S1 117.55(8), C19–Cu4–Cu3 132.73(9), C35–Cu4–Cu3 88.73(8), Cu4–S1–Cu1 88.39(3), Cu4–S1–Cu2 156.22(4), Cu1–S1–Cu2 79.32(2), Cu4–S1–Cu3 80.02(2), Cu1–S1–Cu3 138.54(3), Cu2–S1–Cu3 95.79(3). The shortest Cu···I distance found in the unit cell is around 7.007 Å which excludes any direct Cu–I interaction.

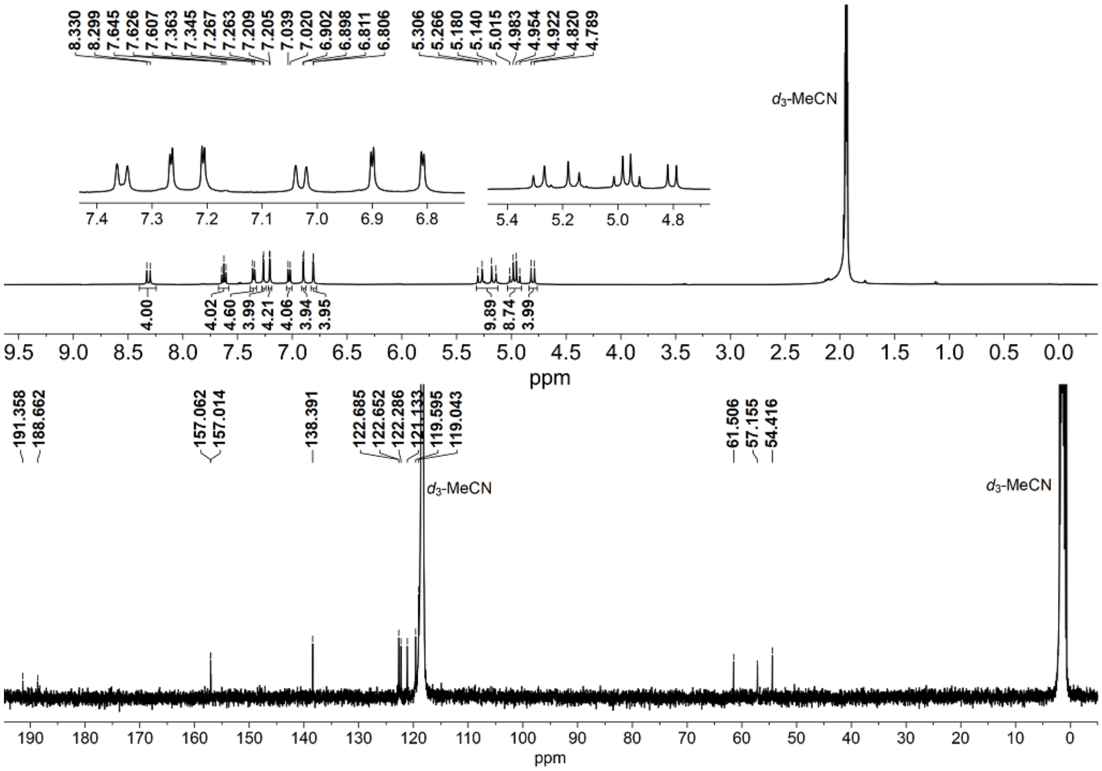

Figure S39. ^{1}H (top, 400 MHz) and ^{13}C (bottom, 100 MHz) NMR spectra of $[L_2Cu_4S]I_2$ (**4**) in d_3-MeCN (298 K).

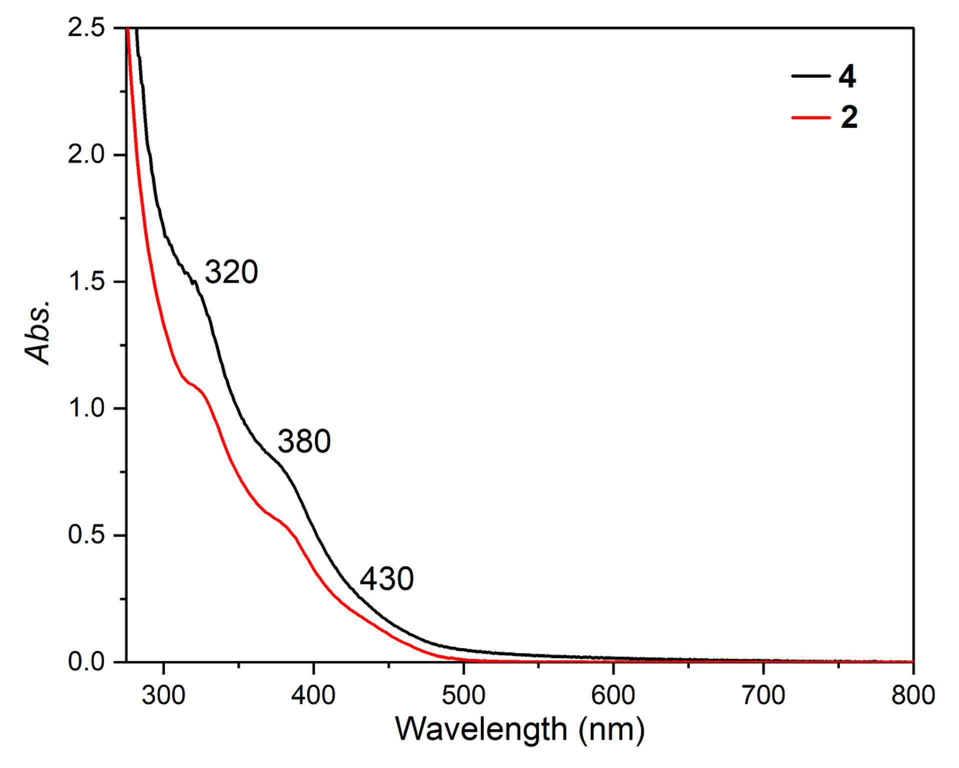

Figure S40. Comparison of UV-vis spectra of **4** (black) and **2** (red) recorded in MeCN at 298 K.

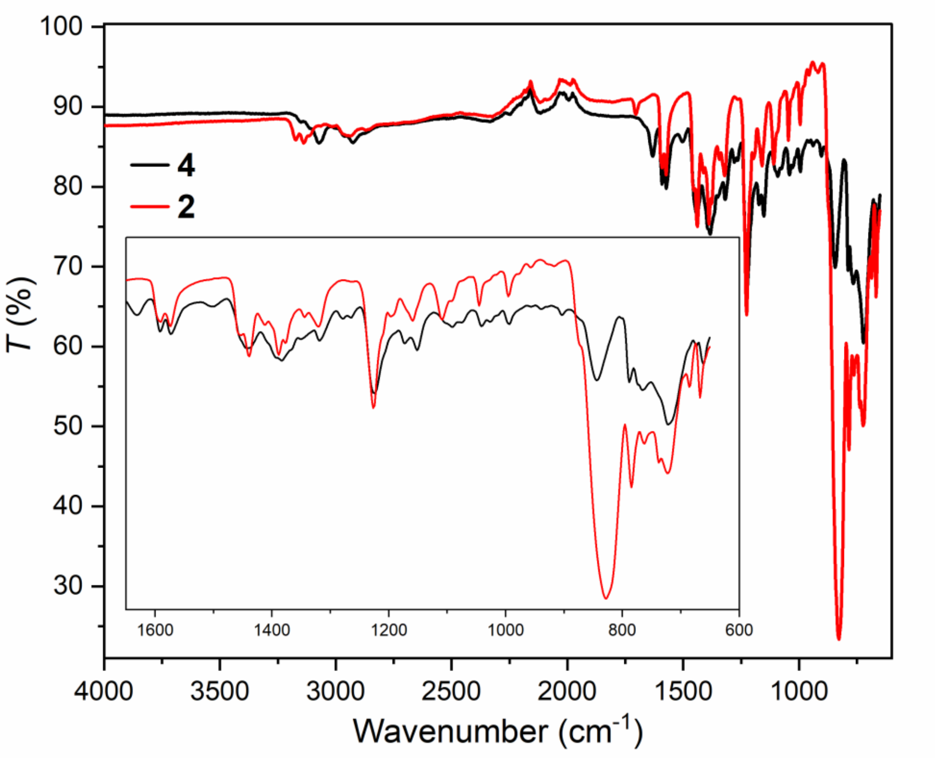

Figure S41. Comparison of ATR–IR spectra (solid, 650–4000 cm^{-1}) of **2** (red) and **4** (black). Insert shows the range from 650 to 1650 cm^{-1}. The intense peak at 830 cm^{-1} due to PF_6^- disappeared in the spectrum of **4**, which indicates the absence of PF_6^-.

Reaction of 2 with [Me3O]BF4

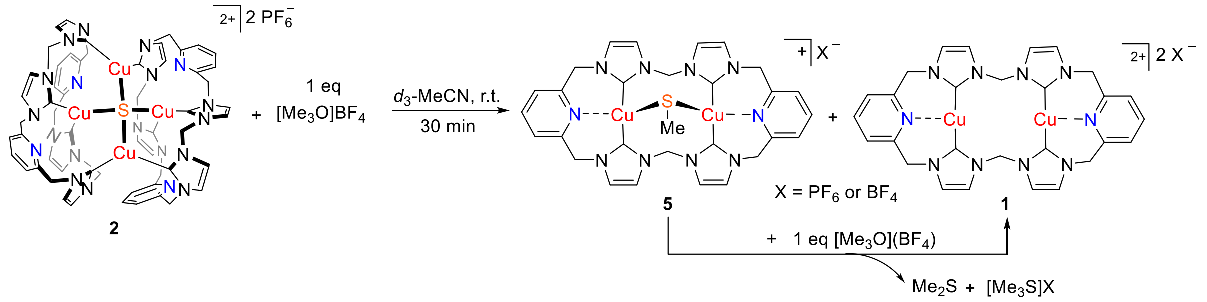

At −35 °C, to a d_3-MeCN (0.5 mL) solution of $[L_2Cu_4S](PF_6)_2$ (**2**, 5.0 mg, 3.2 μmol) in a *J. Young* NMR tube was added $[Me_3O]BF_4$ (120 μL, 26.4 mM in d_3-MeCN, 3.2 μmol) with a Hamilton syringe and 1,3,5-trimethoxybenzene (1.2 mg, 7.1 μmol) as an internal standard. The reaction mixture was allowed to warm up to room temperature and monitored by ^{1}H NMR spectroscopy. During the course of the reaction, the color of the solution remained yellow. After 10 min, ^{1}H NMR analysis shows the nearly complete consumption of **2** and the formation of $[LCu_2(\mu\text{-SMe})]X$ (**5**) (X = PF_6 or BF_4) in 67% yield (based on sulfur, 2.1 μmol) and Me_2S in 15% yield (based on sulfur, 0.5 μmol, δ (ppm) = 2.08); a small peak due to the formation of $[Me_3S]X$ (X = PF_6 or BF_4, δ (ppm) = 2.78) was observed (see Figure S42). Then another equivalent (slight excess) of $[Me_3O]BF_4$ was added (144 μL, 26.4 mM in d_3-MeCN, 3.8 μmol) to the reaction mixture at room temperature. During the course of the reaction, the color of the solution turned to orange. After 10 min, ^{1}H NMR analysis shows the formation of Me_2S in 46% yield (based on sulfur, 1.5 μmol) and $[Me_3S]X$ (X = PF_6 or BF_4) in 28% yield (based on sulfur, 0.9 μmol), and the clean formation of $[LCu_2]X_2$ (**1**) (X = PF_6 or BF_4, see Figure S42). The presence of **1** made the isolation of pure **5** difficult. Further characterization of **5** was done by using a sample synthesized independently through the reaction of **1** with MeSNa. The ^{1}H NMR and ESI mass data of the reaction mixture are essentially identical with the data for the pure sample of **5**.

Note: Signals due to complexes **5** and **1** are expected to be observed in the ^{1}H NMR spectrum of the reaction of **2** with 1 equiv $[Me_3O]BF_4$. However, only one set of signals assigned to the macrocyclic ligand was observed, and the chemical shift of each peak is in-between the corresponding peaks of pure samples of **5** and **1** (Figure S43). In addition, integration shows the ratio of the macrocycle and μ-*SMe* is roughly 2 : 1 (Figure S42). The ESI mass spectrum of the reaction mixture (**2** with 1 equiv $[Me_3O]BF_4$, 10 min) shows that both major peaks due to **5** and **1** were observed (Figure S44). Thus, we speculate that there is an interconversion between **1** and **5** via the exchange of μ-SMe (Scheme S2), which is fast on the NMR timescale. The ^{1}H NMR spectrum of a 1 : 1 mixture sample of pure **5** and **1** shows only one set of signals due to the macrocyclic ligand with chemical shifts similar to those of the reaction mixture of **2** with 1 equiv $[Me_3O]BF_4$ (Figure S43).

Scheme S2. The interconversion between **1** and **5**.

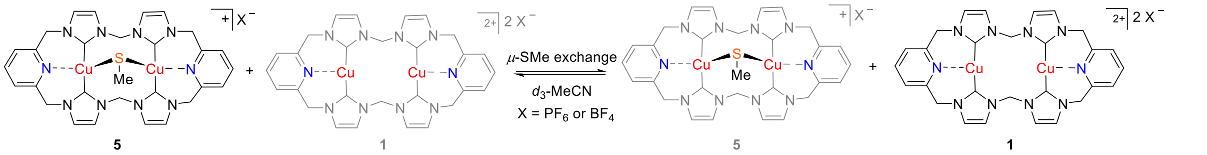

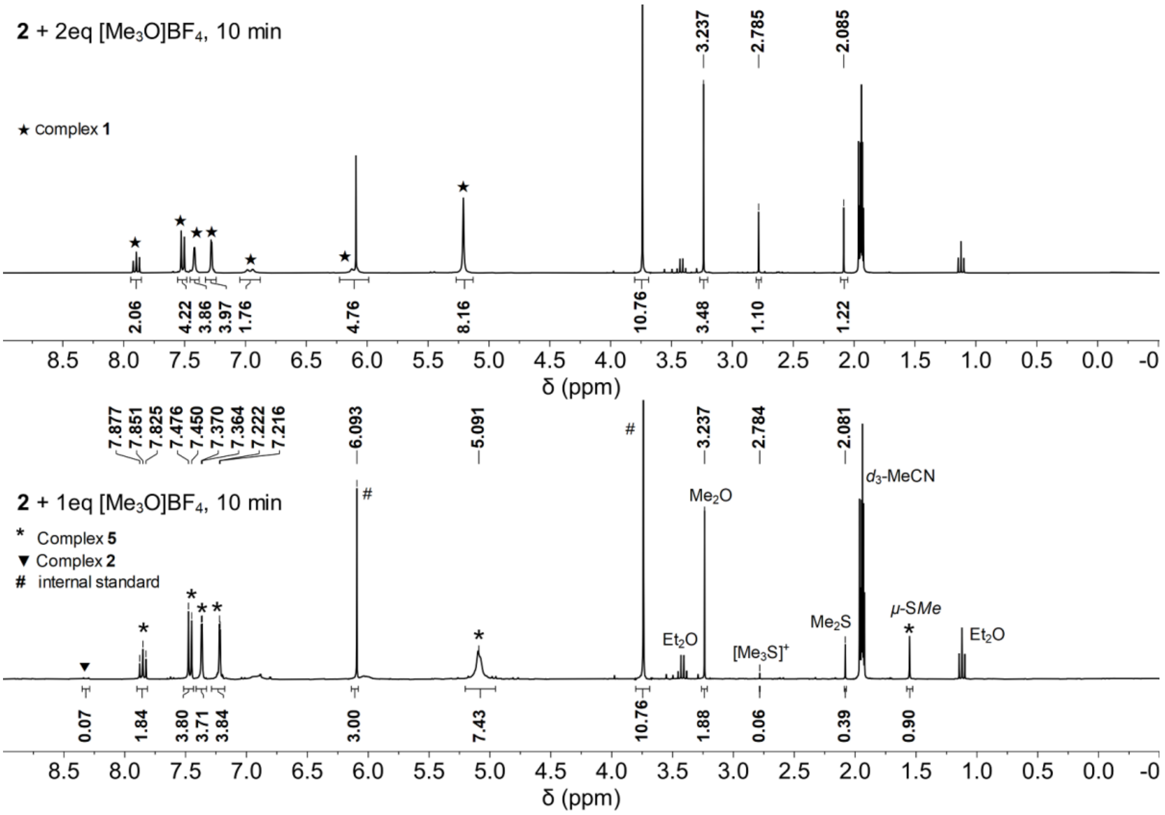

Figure S42. ^{1}H NMR spectra of the reaction of **2** with $[Me_3O]BF_4$ at room temperature in d_3-MeCN (298 K).

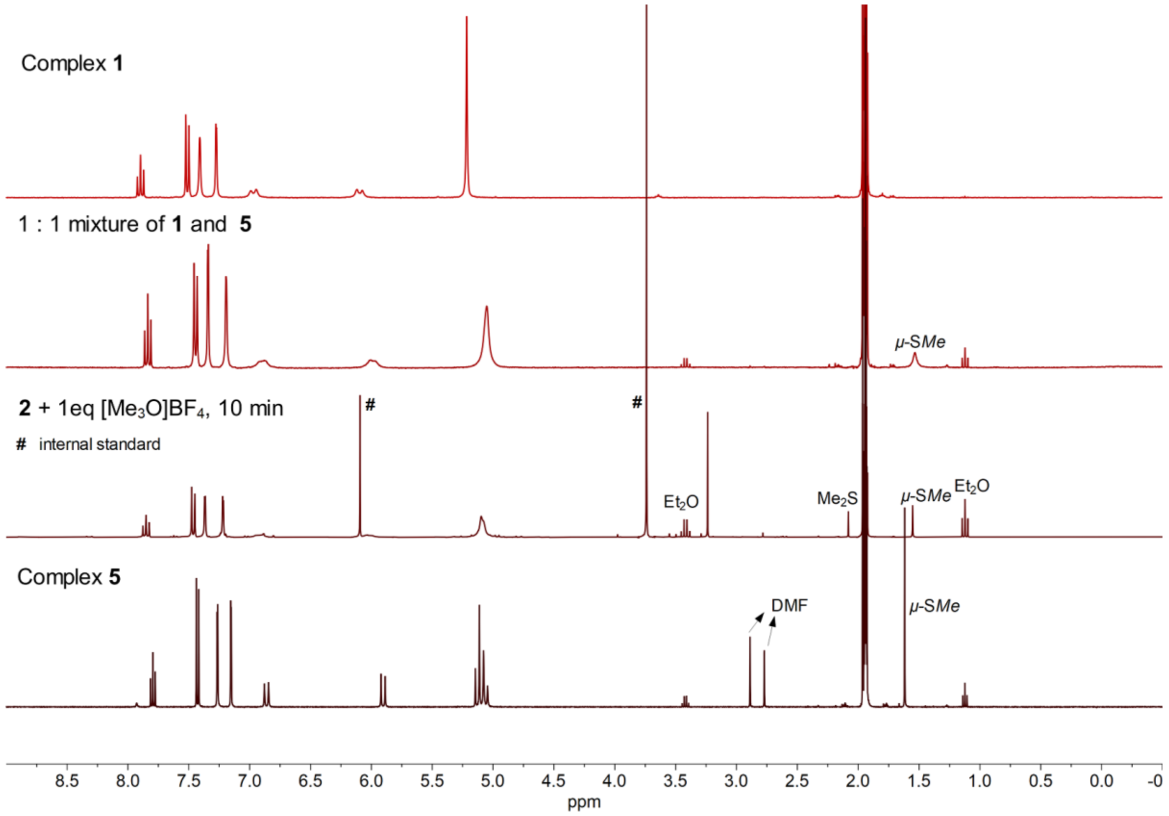

Figure S43. Comparison of ^{1}H NMR spectra of complexes **1**, **5**, the mixture of **1** and **5**, and the reaction of **2** with 1 equiv $[Me_3O]BF_4$ at room temperature, suggesting fast interconversion of **1** and **5**.

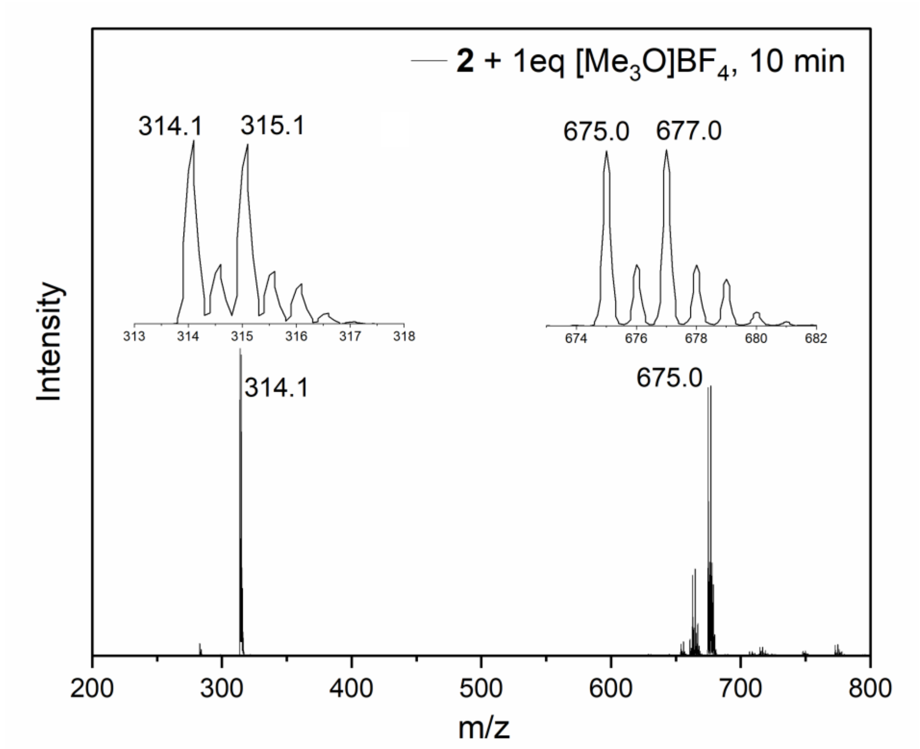

Figure S44. ESI–MS spectrum (MeCN) of the reaction mixture (**2** with 1 equiv $[Me_3O]BF_4$, 10 min) showing the formation of **1** (m/z = 314.1 $[M]^{2+}$) and **5** (m/z = 675.0 $[M]^{+}$). Inserts show the isotope pattern for the peaks around m/z = 314 and 675.

Preparation of $[LCu_2(\mu\text{-SMe})]PF_6$ (5). At room temperature, to the MeCN (7 mL) solution of $[LCu_2](PF_6)_2$ (**1**, 36.8 mg, 0.04 mmol) was added MeSNa (2.8 mg, 0.04 mmol). The reaction mixture was stirred at room temperature for 3 days, and during the course, the color of the solution remained yellow with gradual formation of a yellow precipitate. The resulting suspension was filtered through a funnel with fine fritted disc, and the yellow solid was washed with MeCN (2 mL × 3) and Et_2O (2 mL × 2), and then dried under vacuum to give $[LCu_2(\mu\text{-SMe})]PF_6$ (**5**) as a yellow powder (20.6 mg, 76%). Yellow crystals of **5** suitable for X-ray diffraction were obtained by slow diffusing of Et_2O into the filtrate at room temperature. ^{1}H NMR (400 MHz, d_3-MeCN, 298 K): δ (ppm) 7.80 (t, J = 7.7 Hz, 2H, C*H*-*p*-Py), 7.43 (d, J = 7.7 Hz, 4H, C*H*-*m*-Py), 7.26 (d, J = 1.9 Hz, 4H, C*H*-imidazolyl backbone), 7.15 (d, J = 1.9 Hz, 4H, C*H*-imidazolyl backbone), 6.86/5.90 (ABq, 4H, J = 13.5 Hz, NHC-CH_2-NHC), 5.13/5.06 (ABq, 8H, J = 13.2 Hz, NHC-CH_2-NHC), 1.62 (s, 3H, μ-S*Me*). ^{13}C NMR (100 MHz, d_3-MeCN, 298 K): δ (ppm) 190.8 (N*C*N), 155.0 (*C*H-*o*-Py), 139.9 (*C*H-*p*-Py), 123.9 (*C*H-*m*-Py), 123.7 (*C*H-imidazolyl backbone), 118.7 (*C*H-imidazolyl backbone), 66.3 (NHC-*C*H_2-NHC), 56.4 (Py-*C*H_2-NHC), 19.2 (μ-S*Me*). ^{19}F NMR (376 MHz, d_3-MeCN, 298 K): δ (ppm) −73.0 (d, $J_{P\text{-}F}$ = 705.0 Hz). ^{31}P NMR (162 MHz, d_3-MeCN, 298 K): δ (ppm) −144.6 (hepta, $J_{F\text{-}P}$ = 704.1 Hz). Anal. Calcd. for $C_{29}H_{29}CuN_{10}SPF_6{\cdot}2MeCN$: C 43.85, H 3.90, N 18.60, S 3.55; Found: C 43.71, H 4.20, N 18.33, S 4.13. ESI–MS (MeCN) m/z (%): 675.0 (100) for $[C_{29}H_{29}Cu_2N_{10}S]^+$. Absorption spectrum (MeCN): λ_{max}, nm (ε, M^{-1} cm^{-1}) 310 (14300), 400 (3400). ATR–IR (powder, cm^{-1}): ν = 3167 (w), 3134 (w), 2936 (w), 2897 (w), 2828 (w), 1594 (w), 1578 (w), 1469 (w), 1445 (m), 1393 (m), 1378 (w), 1363 (w), 1346 (w), 1331 (w), 1318 (w), 1281 (w), 1268 (w), 1223 (m), 1169 (w), 1147 (w), 1113 (w), 1092 (w), 1038 (w), 1025 (w), 999 (w), 962 (w), 938 (w), 924 (w), 908 (w), 898 (w), 876 (w), 833 (s), 818 (s), 789 (m), 763 (m), 747 (m), 729 (s), 718 (s), 699 (m), 675 (w), 667 (w), 660 (w).

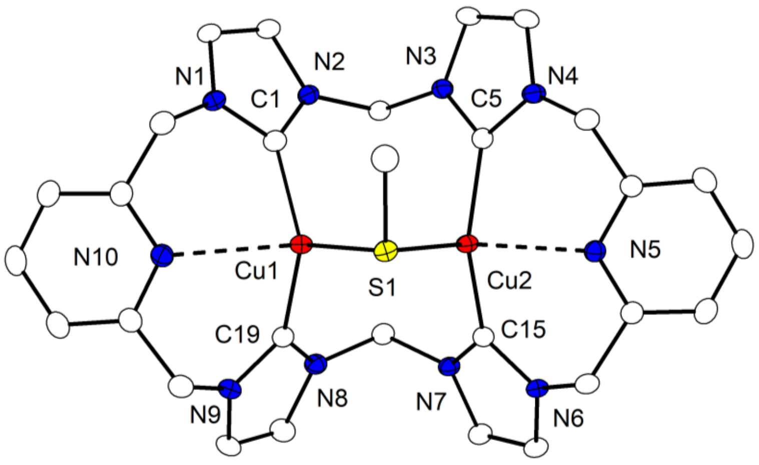

Figure S45. Plot (30% probability thermal ellipsoids, hydrogen atoms omitted) of the molecular structure of the cationic part of **5**. Selected bond lengths [Å] and angles [°]: Cu1–C19 1.957(3), Cu1–C1 1.968(3), Cu1–N10 2.610(3), Cu1–S1 2.2996(10), Cu2–C15 1.935(3), Cu2–C5 1.943(3), Cu2–S1 2.3056(10), Cu2–N5 2.443(3), Cu1···Cu2 2.9794(6); C19–Cu1–C1 130.06(14), C19–Cu1–S1 116.16(10), C1–Cu1–S1 112.43(10), C15–Cu2–C5 136.83(14), C15–Cu2–S1 114.76(10), C5–Cu2–S1 106.64(10), C15–Cu2–N5 88.74(12), C5–Cu2–N5 89.21(12), S1–Cu2–N5 108.83(7), C15–Cu2–Cu1 99.01(10), C5–Cu2–Cu1 98.50(10), Cu1–S1–Cu2 80.63(3).

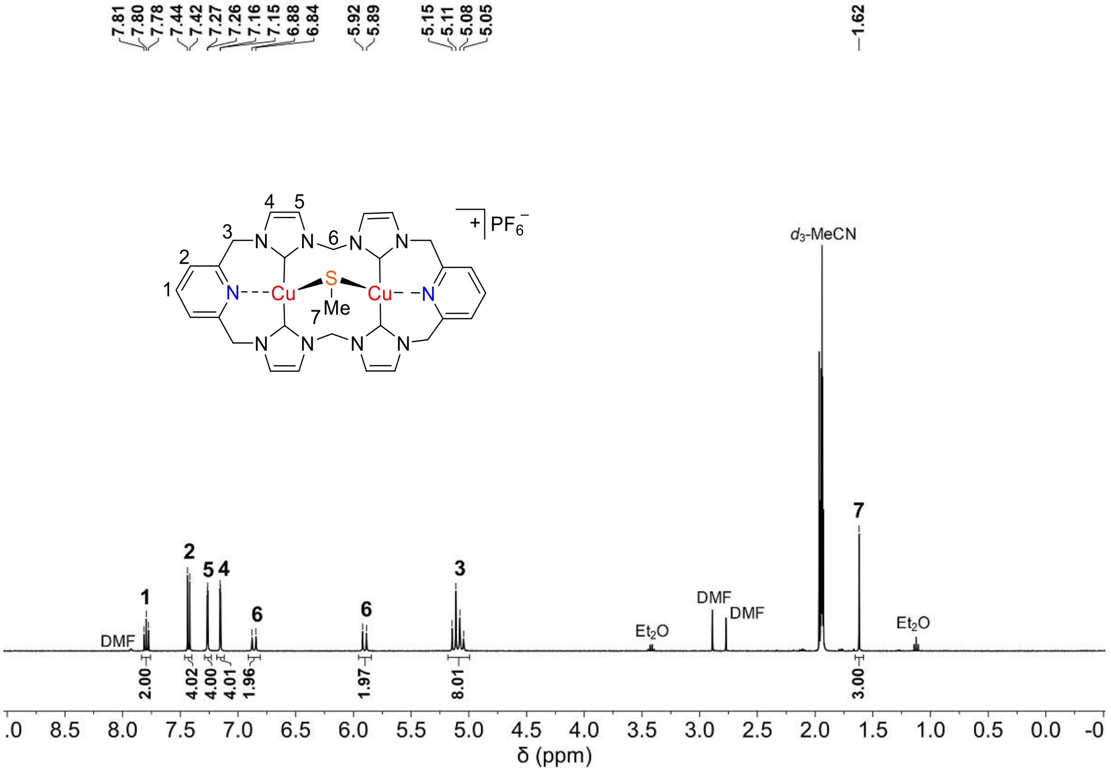

Figure S46. ^{1}H NMR spectrum of [LCu$_2$(μ-SMe)]PF$_6$ (**5**) in d_3-MeCN (400 MHz, 298 K).

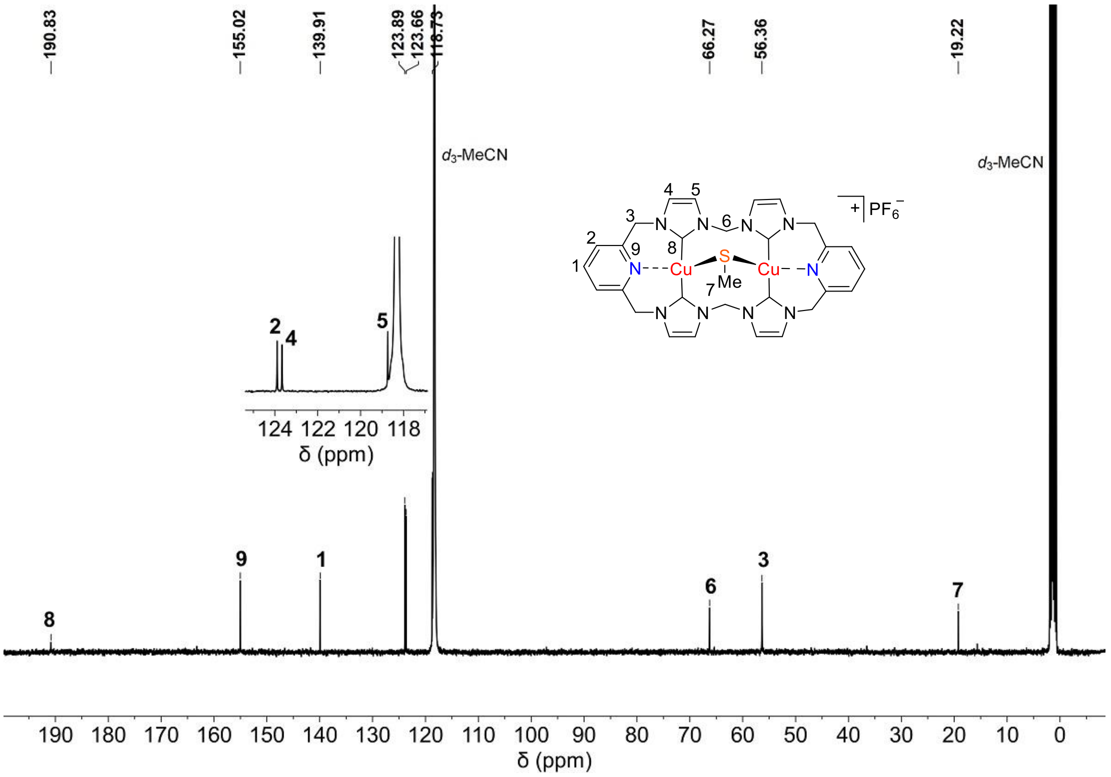

Figure S47. ^{13}C NMR spectrum of [LCu$_2$(μ-SMe)]PF$_6$ (**5**) in d_3-MeCN (100 MHz, 298 K).

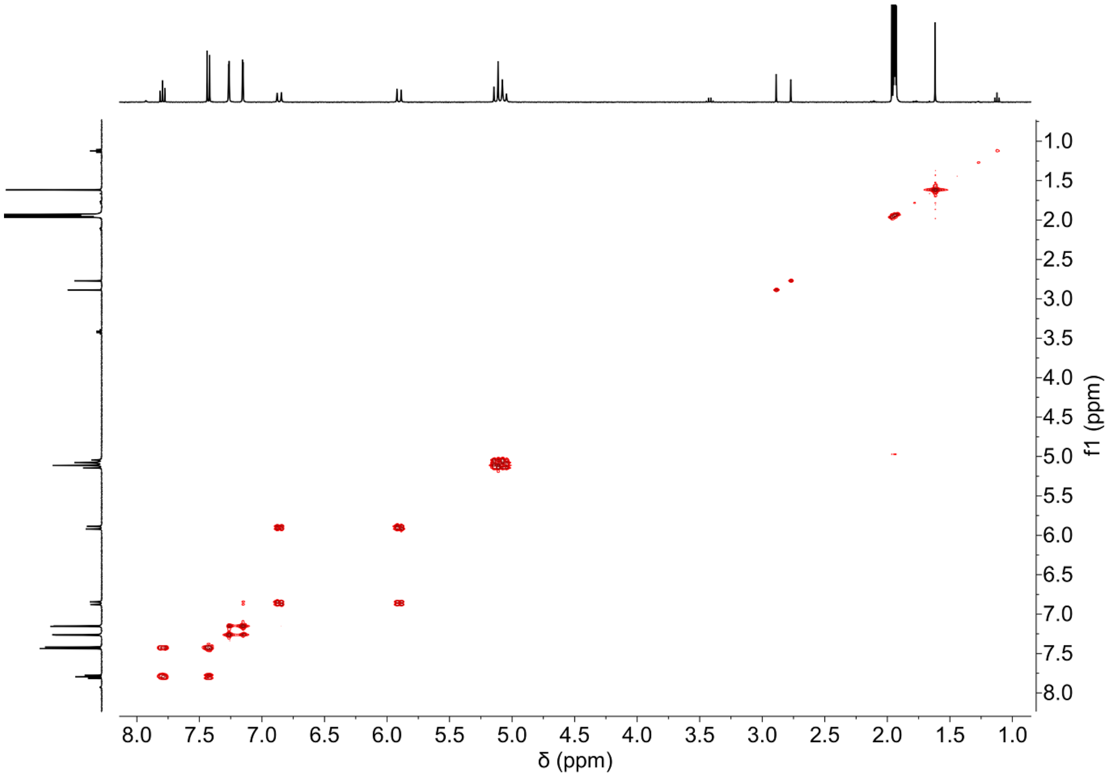

Figure S48. ^1H-^1H COSY spectrum of [LCu$_2$(μ-SMe)]PF$_6$ (**5**) in d_3-MeCN (400, 400 MHz, 298 K).

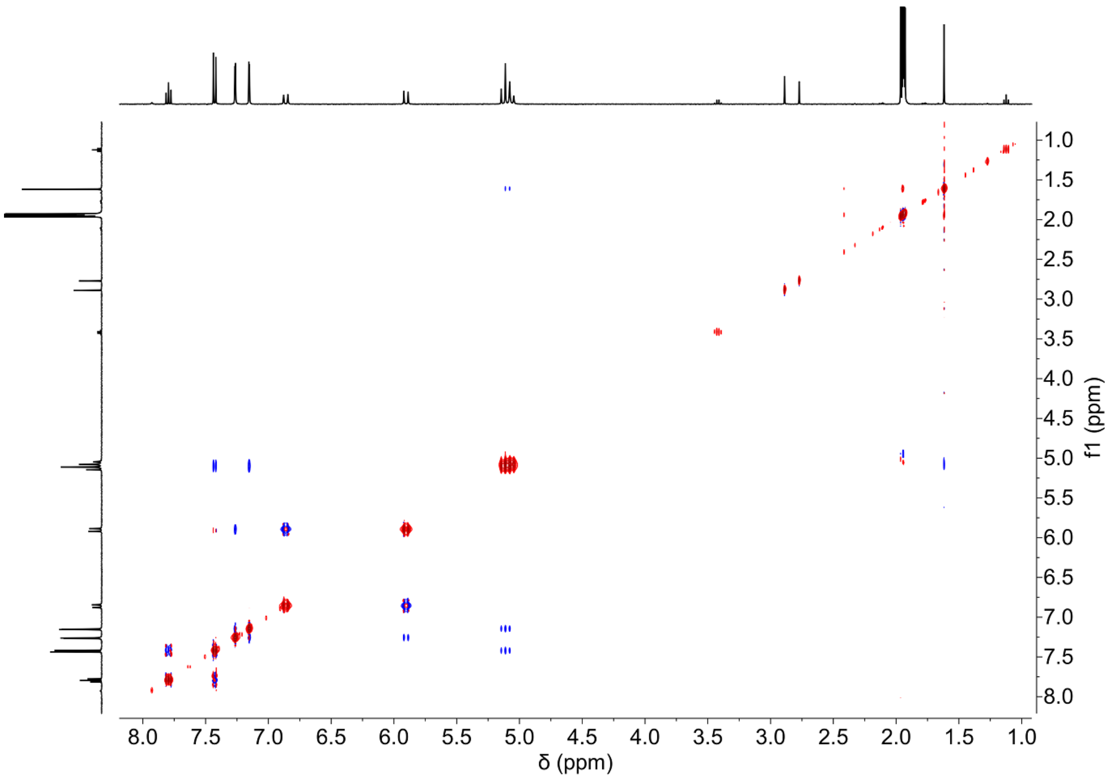

Figure S49. ^{1}H-^{1}H NOESY spectrum of [LCu_2(μ-SMe)]PF_6 (**5**) in d_3-MeCN (400, 400 MHz, 298 K).

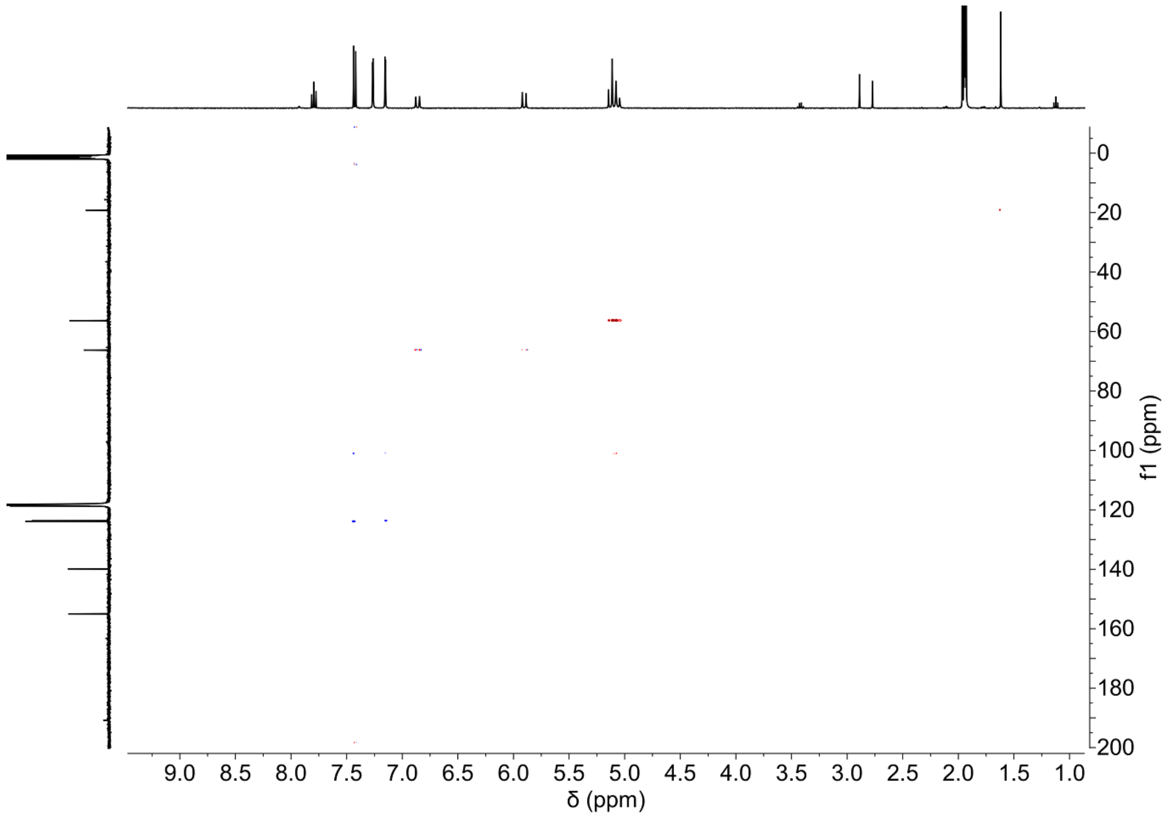

Figure S50. ^{1}H-^{13}C HSQC spectrum of [LCu_2(μ-SMe)]PF_6 (**5**) in d_3-MeCN (400, 100 MHz, 298 K).

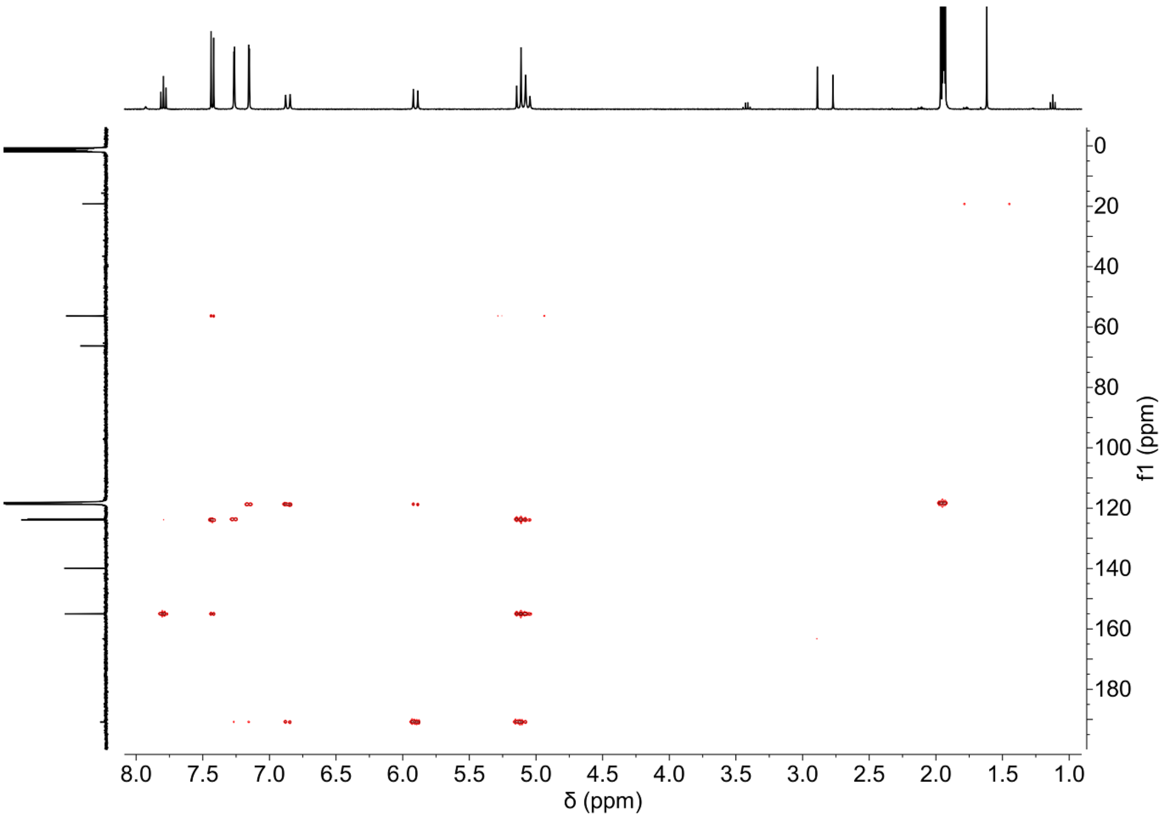

Figure S51. ^{1}H-^{13}C HMBC spectrum of [LCu$_2$(μ-SMe)]PF$_6$ (**5**) in d_3-MeCN (400, 100 MHz, 298 K).

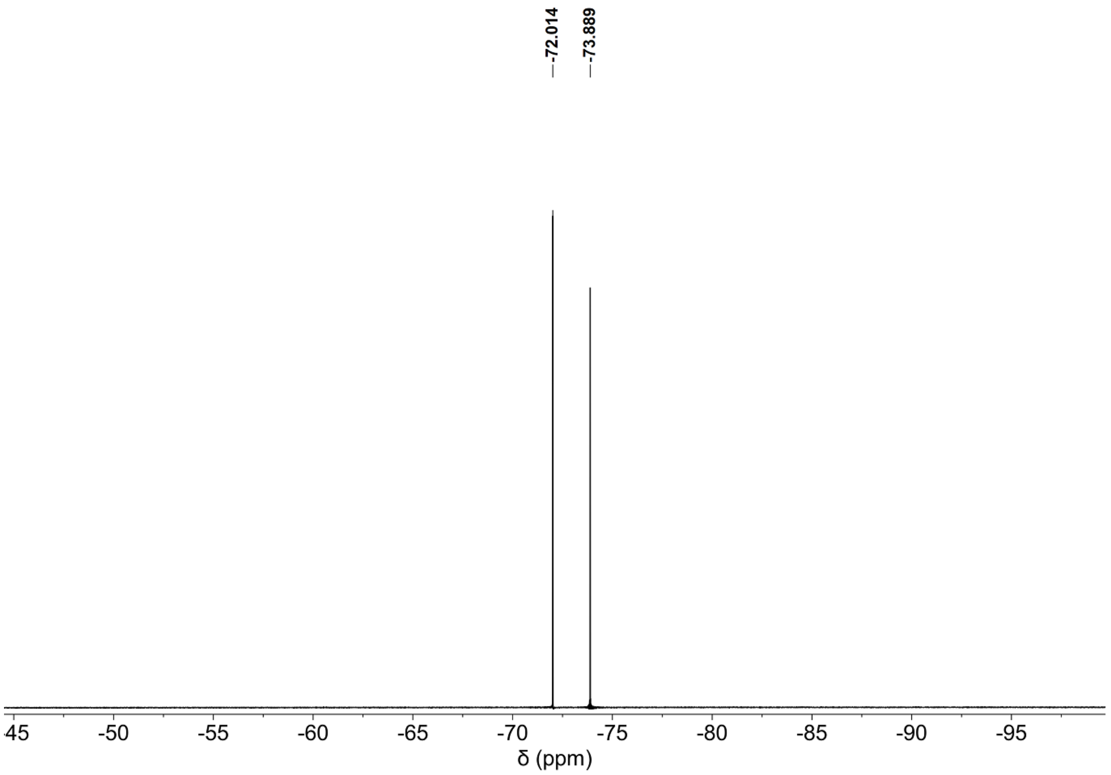

Figure S52. ^{19}F NMR spectrum of [LCu$_2$(μ-SMe)]PF$_6$ (**5**) in d_3-MeCN (376 MHz, 298 K).

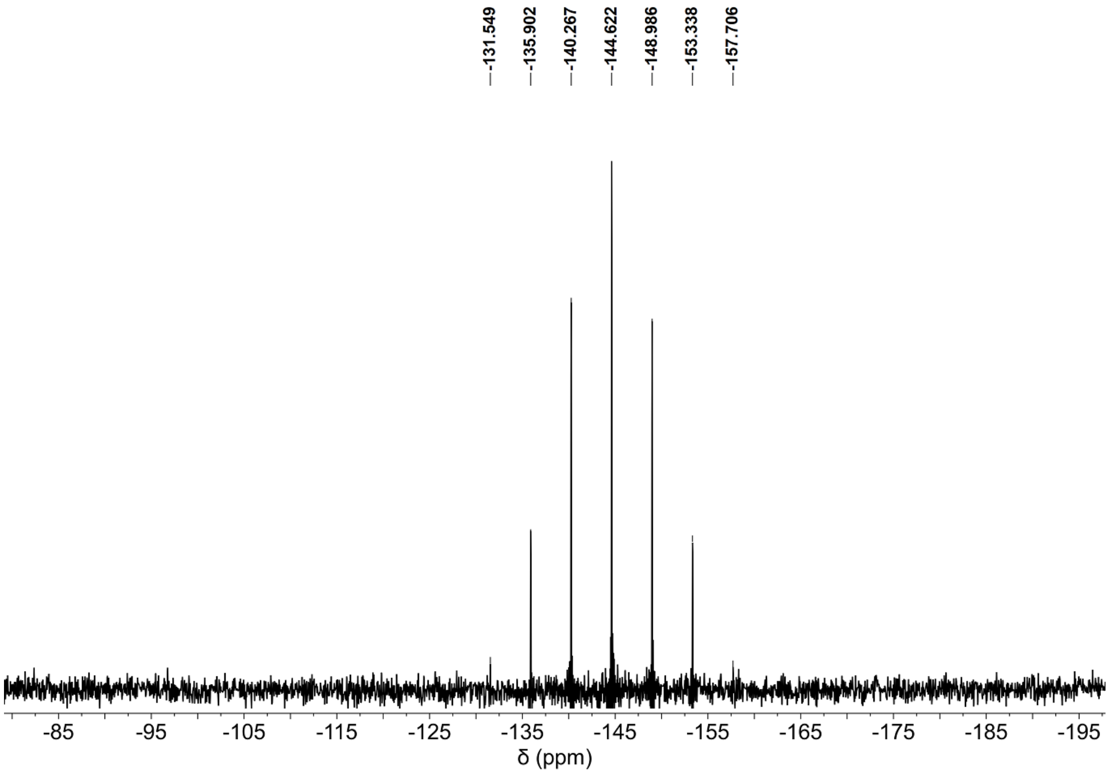

Figure S53. ^{31}P NMR spectrum of [LCu_2(μ-SMe)]PF_6 (**5**) in d_3-MeCN (162 MHz, 298 K).

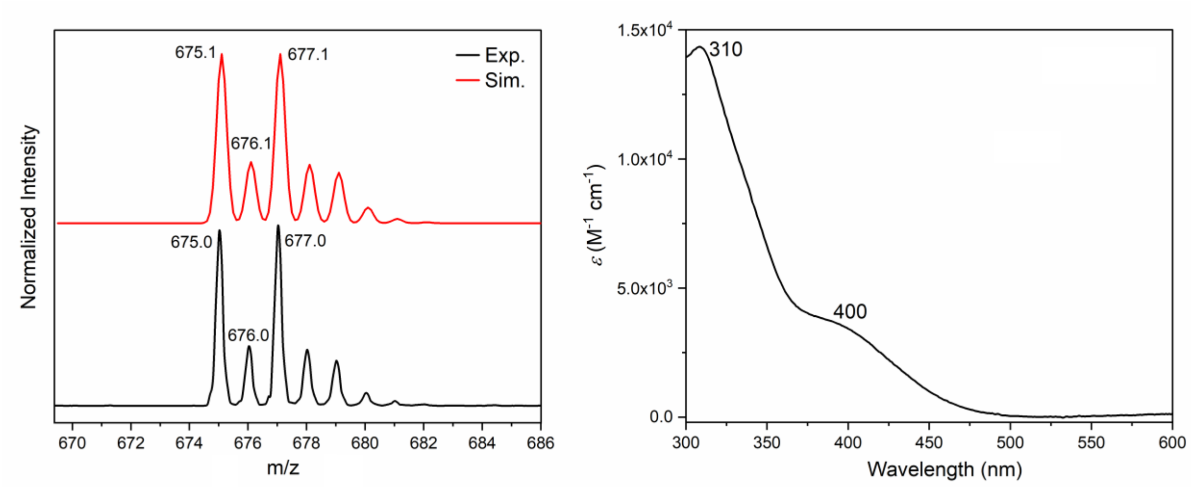

Figure S54. (Left) ESI–MS spectrum (MeCN) of complex **5** and the simulated isotope pattern for the peak around m/z = 675.0 $[M]^+$ and (Right) UV-vis spectrum of **5** recorded in MeCN at 298 K. The positions of the absorption bands are labeled.

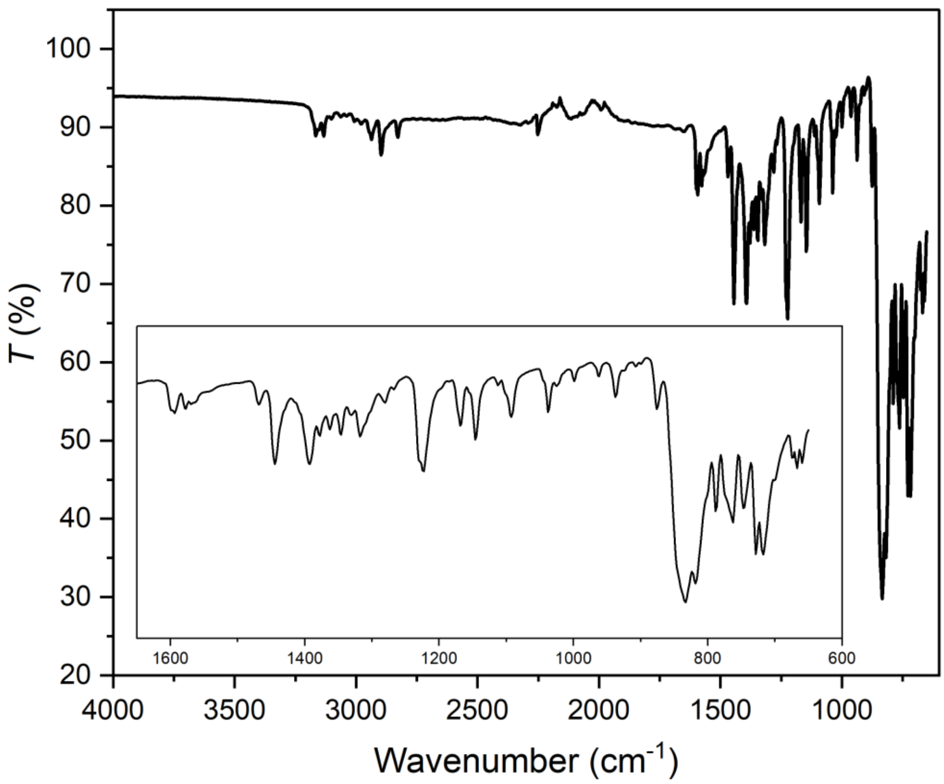

Figure S55. ATR–IR spectrum (solid, 650–4000 cm^{-1}) of **5**. Insert shows the range from 650 to 1650 cm^{-1}.

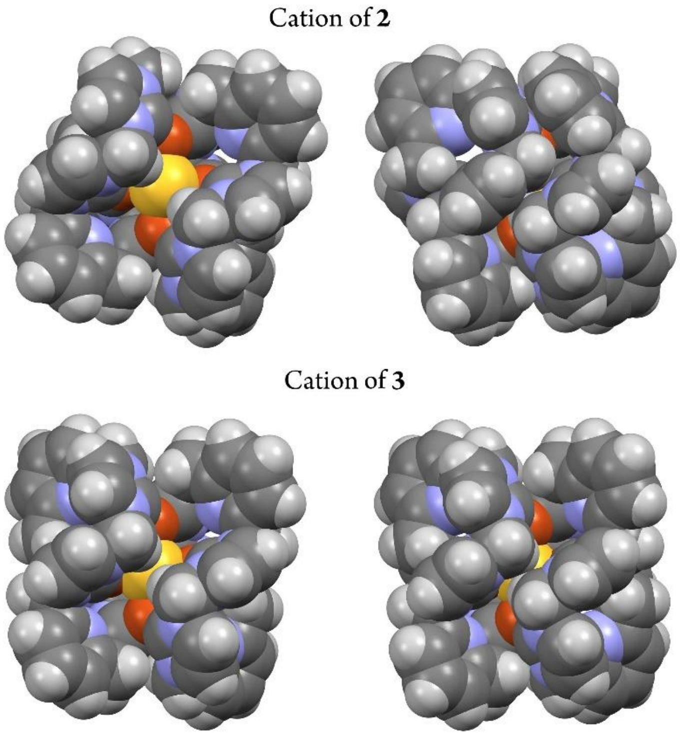

Figure S56. Space-filling model of $[L_2Cu_4S]^{2+}$ (cation of **2**; top) and $[L_2Cu_4S]^{3+}$ (cation of **3**; top) based on the structures determined by X-ray diffraction, showing the shielding of the Cu ions and the open cleft leading to the S center. Left: View along the S-Cu_4 direction; Right: View along the Cu_4-S direction. Color code: C (gray), N (blue), S (yellow), Cu (red) and H (white)."

Table S9. Cartesian coordinates of the optimized geometries.

Complex 2, cpcm(Acetonitrile)

Cu	14.57199755585492	5.14947244610193	11.25574738514955
Cu	12.89765619817866	5.46779575127163	13.92363117442499
Cu	16.68893666693960	2.98649288424627	12.68732078730403
Cu	15.79586636111502	4.49754698090175	15.33938765082699
S	14.64784762068719	3.99404054458929	13.32569961558846
N	10.25872723816502	6.18363206242790	12.66637612101681
C	11.25160655488932	5.26916566923142	12.80790412895004
N	10.90719117141215	4.29965250708023	11.92126594077023
C	9.33855062175488	5.80858443128398	11.70205913779713
H	8.48672486085755	6.41605273801384	11.45155802263248
N	12.33947532411756	3.31664649587769	10.28063718808394
C	9.74281576630181	4.60815850141941	11.23293579473642
H	9.31574823658185	3.95813318761831	10.49013996800818
N	13.51627342766601	3.98692953324339	8.63922978997278
C	11.76017394169576	3.17279789273377	11.60318960051735
H	12.56148648004022	3.14632112935830	12.33905453995954
H	11.18159339981123	2.25170026836651	11.62074458151581
N	16.42510113328936	3.17370430373641	8.58877305872694
C	13.40393370653620	4.11958623716288	9.98679395501255
N	18.56494499003553	3.24277242823299	10.28381721765427
C	11.81006036694907	2.71354047951365	9.15090321319060
H	10.95933181234677	2.05637771526794	9.19398235842052
N	18.32271954193844	5.07647036832364	11.33620285794619
C	12.56254951874538	3.12986444061035	8.11149904879873
H	12.51383395748722	2.89584264783146	7.06283353822481
N	15.27914035169861	8.00360001618949	10.60262586934327
C	15.63736817503396	3.47748822155762	7.55965262404021
C	14.59836628107883	4.53966139543482	7.83232676752321
H	15.03908249801778	5.35540574823449	8.40179598956157
H	14.17856190596985	4.92394784688314	6.90394320373909
N	16.94325475553205	7.03869331150274	11.52034036394883
C	15.73458745073689	2.82629540371060	6.33396356418614
H	15.08683187796943	3.10384438368247	5.51306241378941
N	12.08787979158787	8.02666080742242	11.77469282968927
C	16.67861612777108	1.81537061653405	6.19641678187121
H	16.78168881214950	1.28619107536649	5.25770875914300
N	18.48854739205149	0.95822140218083	13.98999650231843
N	16.53474671715075	0.79571963071300	14.81598407236971
C	17.49753101109671	1.50076110553410	7.27324560108163
H	18.25355992729807	0.73050457552578	7.19531412706620
N	15.09521676213857	1.75310878469281	16.46463637325896
C	17.33968032117751	2.21705016663850	8.45588319786767
C	17.85736192651321	3.80005646736350	11.30047557682542
N	13.32774223090839	5.47649219588887	18.03510534934060
C	18.21968974377027	1.97595854005655	9.66718862580168
H	17.68601962699687	1.38468272487363	10.41262573286179
H	19.12703090871728	1.44362086111821	9.38402084834804
N	15.27040292955073	3.17502008704667	18.03824692229268
N	14.17186654192941	7.86523243110393	15.14352722300403
C	19.28214197258285	5.31575828923932	10.36360140919396
H	19.77264209889317	6.26681906467867	10.25447681874630
C	19.42768048519796	4.15567484642366	9.69086647079584
H	20.06038345667918	3.89464616512341	8.86072883165966
N	12.43516700728117	7.37282519868713	16.27096386014708
C	15.66033917566375	6.80376509071347	11.11551958757684
N	18.11902768082457	6.35077778302300	15.83814124856176
C	17.77423790237153	6.07379875508779	12.21938404017204
H	18.57472586262571	6.61841179462541	12.71391001183337
H	17.16761807318175	5.55062670907088	12.95115289282595
N	16.52366541247369	7.41877876358065	14.91222978933125
N	19.42695426007997	3.41943987535076	14.74668089930059
C	17.34586222253773	8.33800510734181	11.25194399431010
H	18.33054546859954	8.69634972800085	11.49505210477552

C	11.62587344192710	9.56185900380826	13.54432062795432
H	11.04050411104876	9.80891904575434	14.42014675170955
C	12.64690259429895	10.39016264304756	13.10899116823539
H	12.86794180003472	11.31135429895907	13.63233855932529
C	13.39335897074814	10.01344337711420	12.00077866498016
H	14.20173594625147	10.63594236287256	11.64333587143644
C	13.08781564485575	8.81259030403260	11.36722947927766
C	13.91011575084628	8.31094987324978	10.19774860466160
H	13.46506873739079	7.39702297979018	9.81271249027562
H	13.95389323389647	9.05658581750375	9.40382457852138
C	16.29041229860055	8.95051733418381	10.67528445355481
H	16.17036124021158	9.95586965103468	10.31333052185820
C	10.26242225838199	7.47573614473868	13.31812320710468
H	10.35439197496333	7.33684138808763	14.39347629960546
H	9.29689905407677	7.94784922346126	13.13272835723902
C	11.37672471118563	8.38679579146317	12.84237285773215
C	17.23615781724518	1.46755010388926	13.86635035614123
C	18.57122003252470	0.01886897199020	15.00377657174100
H	19.48941648439062	-0.48572797223088	15.24814331320734
C	17.33032439071470	-0.09282911144439	15.52473097021404
H	16.94819489312848	-0.71373782185587	16.31528729071156
C	15.16446001330718	1.10793694741942	15.16693021820469
H	14.57555013271649	0.19433633838105	15.21154278309313
H	14.77556222703169	1.78832072516130	14.41189265962900
C	15.39617367174016	3.06540429770923	16.68996435702985
C	14.79786437744314	1.07513264726561	17.63603438830291
H	14.54868307826018	0.02858696152082	17.64838034427665
C	14.89843251142946	1.97877127493454	18.63269023090776
H	14.73740895000872	1.88553966826463	19.69207650212192
C	15.35547380702767	4.42417046775616	18.78597647424911
H	15.90327060868961	5.13176317544602	18.16687729473407
H	15.90589249204146	4.24136051812970	19.70771190596453
C	13.97051056711526	4.95315180320709	19.07700693925788
C	13.38821100204791	4.85990369163963	20.33719204638894
H	13.94094330408793	4.43917776128880	21.16664444046689
C	12.08580432419790	5.31755505331695	20.49849589884133
H	11.60062515124855	5.26058841031581	21.46449602651989
C	11.41859284070338	5.86201948718468	19.40888455568068
H	10.41015355160802	6.24329363291004	19.50272328565733
C	12.08595901807364	5.92865556531927	18.18952930794588
C	13.20261147889394	6.91800438287407	15.24709143804108
C	11.45437973866720	6.55651543762339	16.96167780390079
H	11.12180719323494	5.78441814870808	16.26646358834515
H	10.59685574255527	7.16699795765402	17.24165312631029
C	12.92732885959165	8.55896860100010	16.80047362059963
H	12.45459493518424	9.05967163008446	17.62719028136416
C	14.02259825998220	8.87704392037066	16.08007390301834
H	14.68840734105085	9.72002349572099	16.13596306731413
C	18.97993397939167	5.25304265491657	16.27058886402344
H	18.34517666267400	4.48455916322170	16.70443245601067
H	19.65867597164382	5.63678135389201	17.03234705250115
C	18.55702193331467	7.65901758656189	15.68859024517837
H	19.53122571463087	7.98404513395523	16.00718865571643
C	17.54460136129045	8.33625551995867	15.10713865865530
H	17.45478250940828	9.36774853476747	14.81559007902445
C	16.85535902708779	6.17564241573246	15.36911831131011
C	15.28095229069928	7.73831022253723	14.23266683532262
H	15.42692402657149	8.66622594330435	13.68561334556479
H	15.03602619216638	6.93321767519333	13.54734454863553
C	19.75125726791682	4.66382077947392	15.10723333125552
C	20.71850348549382	5.40300395836076	14.43298359720537
H	20.97077192276340	6.40412079789305	14.75368100222008
C	21.34788250184810	4.83138433355466	13.33547177786816
H	22.09584542225248	5.38458505916086	12.78241812466602
C	20.99595351156778	3.54791152110564	12.95179532994415

H	21.44977079994970	3.07783384197760	12.08921667400412
C	20.02922158789082	2.87369870785767	13.69137496796791
C	19.63707217960334	1.47016334497721	13.27383309977641
H	19.42719420037655	1.46465175528700	12.20676149704385
H	20.47105128755998	0.78866467100670	13.44538410871775

Complex 2 in gas phase

Cu	14.61180866224482	5.15691613255986	11.20654388787293
Cu	12.92351999034319	5.45517998761684	14.00020202299729
Cu	16.66434746053741	2.99220793844166	12.60892539731727
Cu	15.79248720436852	4.54481078286129	15.38185158509604
S	14.71778909957726	4.09784891193646	13.32058708431248
N	10.30760551097066	6.13531270379011	12.68552792568639
C	11.30408767151729	5.22943621158589	12.85818781492012
N	11.00444217678524	4.26452356524800	11.95225421695481
C	9.43235037738837	5.76184833819686	11.67963977477730
H	8.58589164885228	6.35947991943939	11.39232184734364
N	12.43099674548469	3.23478955435882	10.33844413224323
C	9.86636962691344	4.57063901985503	11.21944255834342
H	9.47325120612796	3.93101050924859	10.45046704689764
N	13.54282799098302	3.89625386908804	8.64936133341516
C	11.87346044387919	3.13889950807215	11.67302633690588
H	12.68366720514669	3.15652866054157	12.40189561623067
H	11.31297760110705	2.20754633420865	11.73947290330394
N	16.42642687190517	3.15272771790275	8.52923599399699
C	13.44902861745031	4.07505300776544	9.99279782558437
N	18.56192117777210	3.24993709528712	10.22213489540968
C	11.91528239100271	2.55953446977636	9.24335301980346
H	11.10836118263238	1.85285225714300	9.32192742938810
N	18.31649180471390	5.07365943869850	11.29287533196853
C	12.62712102814958	2.97012320794753	8.17481162124202
H	12.57212151259249	2.68304027318708	7.14033829372665
N	15.28242130798595	8.04011256468614	10.65641130096058
C	15.64166933218921	3.45801268563781	7.50124443463802
C	14.57452908275167	4.48248031191457	7.80338502479674
H	15.00687696183275	5.31024381984314	8.36243100897279
H	14.11184038969464	4.85642762329192	6.89006279120968
N	16.95526065124037	7.04668556919470	11.53148100548540
C	15.76641122996316	2.83972346118484	6.26091928974242
H	15.12617281236042	3.12139744659086	5.43530732778932
N	12.11373025797585	8.00690110651713	11.84013988769681
C	16.73435108328100	1.85478415879240	6.10782143896818
H	16.85926841657911	1.35130199903611	5.15821940655493
N	18.42802940866646	0.97974100203911	13.97727334029803
N	16.46568026545059	0.87043329609599	14.78979564643149
C	17.54693894683917	1.53224916659300	7.18680028253927
H	18.31607447975116	0.77629987617755	7.09696205238923
N	14.98698546438251	1.80845626511703	16.41081813303449
C	17.35928447628518	2.21990416393078	8.38154605796029
C	17.84943022213141	3.79635568038016	11.24224302723771
N	13.30961320028992	5.48903113449056	18.08961468479690
C	18.22328758699711	1.98124244135440	9.60403377052582
H	17.67421419910572	1.40000421458268	10.34654003388930
H	19.13346870608328	1.44238104098247	9.33893800514318
N	15.18334450426336	3.17324560287790	18.03107939265273
N	14.18597978831804	7.86752932694247	15.18413445606817
C	19.27209428037902	5.32387278800183	10.31869423599297
H	19.75725943555653	6.27633732895932	10.20322248436792
C	19.41924876052623	4.17190339186070	9.63477763144367
H	20.04406756608441	3.92962986228989	8.79350313240889
N	12.45349036204928	7.38759779226411	16.32493532637906
C	15.67705776376128	6.81750174551297	11.10437912742178
N	18.15487735162144	6.36377292054143	15.79510283279633
C	17.77641701883724	6.05221333305707	12.20050377867217
H	18.57990608605188	6.57296946750288	12.71763013862062
H	17.15909706501710	5.51533647467985	12.91649014604324

N	16.53873497239663	7.43462232537570	14.90510387301595
N	19.40368288228918	3.42623781483599	14.69172723042573
C	17.34180775068494	8.36348033496479	11.33541368346915
H	18.32094737539431	8.72676031666253	11.59189265186318
C	11.55951826893838	9.56602232847062	13.56016322548077
H	10.94461476531939	9.81927995719355	14.41381995603372
C	12.54664049257777	10.42891670041249	13.11493809901588
H	12.70823988966289	11.37903439668507	13.60707531036287
C	13.31951198910696	10.05822284344465	12.02285723251070
H	14.08799807688612	10.71896798867024	11.64549077913417
C	13.07538671056339	8.82828474000557	11.41825469600118
C	13.91043406828093	8.33876928713996	10.25262144422808
H	13.47408127973341	7.41789468715649	9.87360752762003
H	13.94344252937005	9.08334872133023	9.45612357907187
C	16.28166569333751	8.99348608097430	10.78593238681975
H	16.16439817517749	10.01163351311752	10.46181082073250
C	10.28058254831158	7.41800837068606	13.34949508340696
H	10.38121836369576	7.26505336020748	14.42258306218958
H	9.30327622136163	7.87118015645725	13.17512005613299
C	11.37144248733101	8.36427134389350	12.88379394882783
C	17.18074237256469	1.49385666500057	13.81970696604226
C	18.49469644215925	0.08980768823747	15.03578632146837
H	19.40359269322941	-0.40973965887209	15.31958101596632
C	17.24916529882082	0.01087673290995	15.54690194163292
H	16.86202423363949	-0.56785885956712	16.36542925638416
C	15.08889634725510	1.19961284028458	15.09946315322511
H	14.47897124127730	0.29739951886445	15.09683168443619
H	14.73891997059473	1.90698324331399	14.34760709793304
C	15.33843039853730	3.09827304865324	16.68358608083335
C	14.62505850756175	1.11192553798775	17.55312287693269
H	14.31692734739151	0.08169195035746	17.53390443241812
C	14.73819670221384	1.97986143605352	18.57833953794529
H	14.53620388966367	1.85919311096585	19.62728837352531
C	15.31237584620367	4.39547278829833	18.81377118330465
H	15.87078619714883	5.10259878782097	18.20313221809370
H	15.86976941245003	4.17482931442344	19.72406771286103
C	13.94680326772988	4.95807785640678	19.12788372562474
C	13.37383112305843	4.88374371861199	20.39378222131498
H	13.92291183701511	4.46289104353386	21.22583083497056
C	12.08202766183093	5.36598941348546	20.56363837876027
H	11.60376401323142	5.32422819422172	21.53336516255837
C	11.41617246099362	5.91283956149975	19.47455985205085
H	10.41269741574177	6.30500884767471	19.57669396451501
C	12.07897150639518	5.95971938913327	18.25213391490351
C	13.21484954398653	6.92106159887019	15.30024628388644
C	11.46246329993511	6.58155885735213	17.01440567192393
H	11.14089303633746	5.80314142630036	16.32057434543865
H	10.60050867624951	7.19584968680245	17.27658262172580
C	12.95806296882076	8.57055076405114	16.85020556873699
H	12.50463024500745	9.07439582425284	17.68532194410784
C	14.04857069790702	8.88087001857957	16.12161761864731
H	14.72069179611288	9.71769602895349	16.18422031858682
C	19.00551543835016	5.25611996694037	16.22359118980885
H	18.35851845568948	4.49213393907369	16.64773808323866
H	19.68809268091901	5.62461936538405	16.99059912122408
C	18.60538261819963	7.66073522201472	15.59599499653775
H	19.58748167470169	7.98957628466362	15.88378722151301
C	17.58045405569653	8.33968418055055	15.03774896641619
H	17.49934612977453	9.36869271965703	14.73612542886189
C	16.86950779565351	6.19735905307981	15.38156374613711
C	15.27667320980733	7.73727459015837	14.25320476259536
H	15.40359437769746	8.65738121499374	13.68628760492138
H	15.02495176149622	6.91770139472368	13.58443393909478
C	19.76837374560549	4.65392479718600	15.06153451126093
C	20.77563975463442	5.35796036616546	14.40756134996415
H	21.07031607451551	6.34350277586112	14.74121518929342

C	21.40749979726422	4.76352441880539	13.32366950713688
H	22.19712481006250	5.28140303133966	12.79513299520587
C	21.01720901341814	3.49316237084581	12.93468784352161
H	21.48496529782320	3.00345299765167	12.09056337154848
C	20.01139091272784	2.85606959167650	13.65571469313956
C	19.58083626832439	1.45875306120206	13.25038372074318
H	19.35571510108716	1.45484856300626	12.18579174887462
H	20.40217236231409	0.75910697519176	13.41375455219342

Complex 3-BF_4, cpcm(Acetonitrile)

Cu	1.82552971851477	1.46698810068659	0.19501879046073
Cu	1.46031939184776	-1.70298347471602	0.04773342538482
Cu	-1.82391216862788	-1.48125977233647	0.14977840152305
Cu	-1.45225495108473	1.69520121924881	0.03861035159532
S	0.00063605460269	-0.00692410809576	0.25419329129113
C	3.30004131886663	1.01115262968573	1.43950711139168
C	1.65299860932783	2.92696871433147	-1.13559734277495
C	1.91837338420696	-2.11507153598601	-1.84216214512499
C	2.04332754797974	-2.44773162590986	1.80687357543113
C	-1.62639295788658	-2.92840817877408	-1.18990756992611
C	-3.31377006385066	-1.03036452183207	1.37703007734542
C	-2.05947061014713	2.42303029298438	1.79690010172010
C	-1.87794853051631	2.12352204009591	-1.85526645582587
N	4.61092648282304	1.31252571301609	1.27177170601592
N	3.27917728319653	0.33214642032132	2.61682911031628
N	2.09970037542813	4.20053604801460	-1.01983044860868
N	1.10379952334524	2.89509180312132	-2.37779750311668
N	-1.05926643794920	2.03274126525549	-2.94241685390841
N	2.20299269877414	-1.77781110299085	2.98363167168304
N	2.28827544192331	-3.73481934818341	2.14301801429077
N	1.11706966349082	-2.01749912801883	-2.94160251504601
N	3.11800620431233	-2.46697600368732	-2.35854963113356
N	-2.07322277527919	-4.20340903118469	-1.09241511276384
N	-1.05494790906459	-2.88554478622188	-2.42168552643416
N	-4.62291500528227	-1.32613065279096	1.18757423192420
N	-3.30801426882402	-0.36164291723700	2.56038040758998
N	-2.23238650948572	1.74131366569017	2.96506314694315
N	-2.30653730855178	3.70689884136863	2.14366906529423
N	-3.06908238073179	2.47938663000528	-2.38842133039277
N	4.88393152555639	-0.40711532650117	-0.86104670365917
N	-0.20952112203714	-4.96658596858962	0.76905833809206
N	0.20710308167633	4.95027603643344	0.81976920436116
N	-4.86078281161522	0.41133244017037	-0.93538330076043
C	0.36728754997443	1.76069957976397	-2.88896484201680
C	5.39146295768383	0.81424923123875	2.30311740919983
C	5.16598947906753	1.83057582823447	0.03594760299695
C	4.54925050088762	0.19964601930379	3.16042263872874
C	2.10550493317461	-0.33608313309441	3.13972542771530
C	2.55413900288467	4.77940345415167	0.23002026796351
C	1.81319657549521	4.95234330215137	-2.14920388769952
C	1.18921518621825	4.12500673835108	-3.01447025198494
C	-0.31019572205099	-1.74606838381903	-2.90943372228948
C	5.60806370460790	0.70895854638707	-0.88329110643459
C	1.42068723651013	5.47947514964248	0.95332488480255
C	-1.73203441498766	2.32846849166924	-4.11793153324782
C	2.54273801954646	-2.63359137235461	4.01979354366391
C	2.58757531049082	-3.87397018494902	3.48946035758744
C	2.15562667054040	-4.87221485506322	1.23507750141787
C	1.80866970089275	-2.30517760072127	-4.10814845673664
C	3.07750749945081	-2.58066175417540	-3.73966445341169
C	4.34339704090131	-2.64429053496127	-1.58178558645112
C	-2.54784475971967	-4.79309244336229	0.14472959499434
C	-1.76528440457068	-4.94558758537892	-2.22251871222971
C	-1.12706038044122	-4.11042633334948	-3.06972362691835
C	-5.41705256156288	-0.83396539564890	2.21139918938932
C	-5.16086123329712	-1.83273229864366	-0.06049142135838

C	-4.58559862342474	-0.22962013257755	3.08631281532446
C	-2.14014690826186	0.29777090826609	3.10699047356065
C	-2.58169727320916	2.58690353233675	4.00635123707397
C	-2.61947395755734	3.83268733702275	3.48833045176653
C	-2.16450488277741	4.85365443771920	1.24909510833567
C	-3.00650642233612	2.60211551168908	-3.76792621327901
C	-4.30619149370174	2.65372778201323	-1.62983347992579
C	6.71412767521065	0.86272878485314	-1.71554642220376
C	1.65812509740981	6.61548488593855	1.72279046590488
C	0.81413793407878	-5.54721700026357	1.39697827749318
C	5.22697342839119	-1.42221886529055	-1.65538121877084
C	-1.42528931879690	-5.49534729172202	0.88304268859033
C	-5.58755410001037	-0.70247425353455	-0.97633611539408
C	-0.82608934567332	5.52864021004769	1.43392531679783
C	-5.19108203333452	1.43414993834909	-1.72527752903467
C	7.06980664115499	-0.19010192836910	-2.54585314703343
C	0.58556888995297	7.21529547711027	2.36674893272012
C	0.65906427418764	-6.68879687775883	2.17566775184326
C	6.31767472844387	-1.35872981336934	-2.51530947111035
C	-1.67487013135057	-6.63307616482270	1.64615846563761
C	-6.68328576125669	-0.84620219398416	-1.82394133383707
C	-0.68338486580999	6.66809124531256	2.21805913916478
C	-6.27097637492549	1.38095777554588	-2.59938019160936
C	-0.61231412079391	-7.23545737446108	2.30412596329363
C	-7.02565303008808	0.21467888133313	-2.64955528158682
H	0.56072159700132	0.93287500095999	-2.21674797284060
H	-0.51495119966994	-0.92501753500771	-2.23221246262429
H	0.69942164533934	1.51349750783867	-3.89432988954720
H	6.45859269773979	0.94756946911531	2.33359031561620
H	6.00231542223659	2.48935696704631	0.26326476484614
H	4.38954708195020	2.41568061445471	-0.45636901514320
H	4.73136592437127	-0.30399188752904	4.09328374199365
H	1.24670144151463	0.03062528859064	2.58877003585161
H	1.99396196104842	-0.12012549276632	4.19941712369636
H	2.93514009229264	3.96561448177976	0.84816412141244
H	3.36907242964154	5.47481683082075	0.03453933363776
H	2.08084959429883	5.99103284192664	-2.23303079119856
H	0.81221392157985	4.29195650547649	-4.00805106114736
H	-0.62543677019874	-1.48868832992845	-3.91765809508351
H	-1.24398140634751	2.32051662300873	-5.07648504774230
H	2.72098796661094	-2.28200830748357	5.02073142387988
H	2.79940955676262	-4.82855468225235	3.93725298252932
H	2.96674831731924	-5.56750628284032	1.44412117655147
H	2.25512473103202	-4.49101605161624	0.22194211907818
H	1.33599753495018	-2.29103645637914	-5.07430576475323
H	3.94275656678843	-2.84358498240385	-4.32172750991059
H	4.86219948598551	-3.52060688917471	-1.96660538644892
H	4.04784933867440	-2.82214671191820	-0.55083778971413
H	-3.35680786439861	-5.48965433782925	-0.07045312175962
H	-2.94271421198443	-3.98532659515775	0.76207472129709
H	-2.02970375871457	-5.98394669151913	-2.31962494311758
H	-0.73235865920230	-4.26893608992773	-4.05779838089860
H	-6.48494086250109	-0.96393133402163	2.22488834858281
H	-4.37821652639542	-2.41518220632306	-0.54609585178726
H	-6.00178193203134	-2.49150637778059	0.14910904341863
H	-4.78008008967328	0.26639033880538	4.02078180420664
H	-1.27459163403564	-0.06520072039071	2.56412405027238
H	-2.04403849557035	0.07013760039271	4.16577951757969
H	-2.77100229819553	2.22523215974856	5.00164017217094
H	-2.83488064518603	4.78290096159670	3.94366519294412
H	-2.97904525356462	5.54580457567476	1.45522742063120
H	-2.25106759908495	4.48270184202388	0.23095145373047
H	-3.86242286486787	2.86895248874694	-4.36188892763361
H	-4.02640236081080	2.82436430151631	-0.59324896653776
H	-4.81730763581669	3.53357821599147	-2.01687903344587
H	7.28519425762250	1.78177155783864	-1.70031644256899

H 2.65750343237190 7.02318460521659 1.80067926935592
H 7.92825132825828 -0.10698479297116 -3.19989890765231
H 0.73315339063515 8.10411110459013 2.96663978786262
H 1.51346354153980 -7.13845781249592 2.66370560990122
H 6.57284275884971 -2.20461503590529 -3.13961259605033
H -2.67548991055659 -7.04044979746955 1.70820105187639
H -7.25685217850916 -1.76382834218813 -1.82398175980160
H -1.54516615100231 7.11565714235518 2.69488589309597
H -6.51613204641798 2.23282583787593 -3.21954406446868
H -0.76933898823487 -8.12572771938849 2.89944321688090
H -7.87598431760105 0.13958676314716 -3.31506572160495

Complex 3-BF_4 in gas phase

Cu 1.84704130956528 1.45467861723452 0.25115325685645
Cu 1.44923627273277 -1.71478632436923 0.06800647201099
Cu -1.84668133610801 -1.47157323214687 0.20956602579579
Cu -1.43780255612379 1.70237276332400 0.05670020152115
S -0.00167609162007 -0.01040547315729 0.32001201324326
C 3.35043095375078 0.97686382642619 1.45433785278606
C 1.66851302034156 2.92635600093551 -1.06721003443459
C 1.88682975688951 -2.08933180838973 -1.83757272301725
C 2.09413500821798 -2.50564560556896 1.78725134277707
C -1.64405329786850 -2.93061843900995 -1.11800698682612
C -3.36629466433154 -0.99888032588924 1.39291433384220
C -2.10739295058548 2.47889001832743 1.77390677357143
C -1.84987379935939 2.10167569203655 -1.84944799781921
N 4.65315229972197 1.29925148350809 1.26479511701481
N 3.36660460248439 0.26337635203295 2.61181201712236
N 2.11878669865398 4.19727819052080 -0.94275849519581
N 1.13028897756467 2.90223852055582 -2.31575083337834
N -1.01506319196235 2.01197932592732 -2.92575603955120
N 2.29709207584508 -1.85682621196050 2.96975491703622
N 2.34548099937417 -3.79886182095773 2.09222529633744
N 1.06767756406872 -1.99053807509400 -2.92504576161715
N 3.08426231996793 -2.41517273155573 -2.37494101015899
N -2.09024188009191 -4.20440157609674 -1.01170941677547
N -1.08367186936834 -2.89359101111717 -2.35645741162669
N -4.66706448588241 -1.31379667466129 1.18020778834881
N -3.39743753137352 -0.29391633618599 2.55523385748197
N -2.32765111684554 1.81971888518377 2.94768165489198
N -2.36170110357615 3.76956268933575 2.08700303820630
N -3.03866989610862 2.43539988069570 -2.40081247534301
N 4.86409665146067 -0.36246354258390 -0.91190651187524
N -0.20343654537071 -4.98647776632504 0.79869316646805
N 0.20468505441806 4.96657788356284 0.84508699253076
N -4.84168782017944 0.36915724604468 -0.98321964490235
C 0.41461034147057 1.76500237104630 -2.84225767928838
C 5.46469234059499 0.77664857038809 2.25974532390937
C 5.17314968651377 1.84480584631696 0.02302421913346
C 4.65271527592990 0.12728104708194 3.11790858568489
C 2.20876396187558 -0.41823176326283 3.14630865976432
C 2.55511650039473 4.77181515829471 0.31800026605276
C 1.83990217067895 4.95682237278558 -2.06895437732353
C 1.22171551124524 4.13808790783760 -2.94357901426948
C -0.36390023312831 -1.74871594029851 -2.86054774984420
C 5.61798292120651 0.73281511883325 -0.90837174595915
C 1.41236076929046 5.50041633834145 0.99975395144634
C -1.67859559145444 2.29122660242675 -4.11285641393727
C 2.66701579006687 -2.73372025066937 3.97931799753687
C 2.68798539651486 -3.96293767513245 3.42512986463910
C 2.17152955668903 -4.91912092567264 1.16825364717536
C 1.74902843327868 -2.25643875125100 -4.10508336327401
C 3.02609889443864 -2.51431201764837 -3.75582533387633
C 4.31963455751795 -2.59758193369498 -1.61179942147912
C -2.54627872105619 -4.79075243069105 0.23651726711829
C -1.78757355332732 -4.95350613256057 -2.13874308055707

C	-1.15778718161846	-4.12483445443499	-2.99561317808030
C	-5.49204406808430	-0.79487473852686	2.16594201907435
C	-5.16863716117418	-1.84692313323083	-0.07445332521861
C	-4.69090453247616	-0.15578024748804	3.04186214982361
C	-2.24559050276254	0.37935418450852	3.11287960371699
C	-2.71059267461666	2.68803303960680	3.95977629056993
C	-2.72243986310164	3.92217011434705	3.41644290414616
C	-2.17535352747314	4.89818355504947	1.17580964147814
C	-2.96011214470000	2.54825554765043	-3.77970595644068
C	-4.28439389039187	2.61075367165651	-1.65316623360618
C	6.75601142425189	0.86168978948946	-1.69950859673462
C	1.63085406105521	6.66328061091873	1.73234752581230
C	0.83643405969464	-5.59717933344877	1.36464962056686
C	5.21826698321510	-1.38656174360783	-1.68649040260648
C	-1.41321450672542	-5.52137006660111	0.93234511471976
C	-5.59669144225993	-0.72524490803478	-1.00224316744869
C	-0.84371149309590	5.57518773665123	1.39715186655365
C	-5.18288499962422	1.40138294380048	-1.75297840679448
C	7.11907355444811	-0.20033087472100	-2.51513128002019
C	0.53996877806491	7.29320985677108	2.31400891425179
C	0.70629714234999	-6.77044420296187	2.09905472100230
C	6.34148983516421	-1.35202767424616	-2.50524096323953
C	-1.64270499883384	-6.68725783916797	1.65679772441429
C	-6.72224970228555	-0.84499568708377	-1.81246867684703
C	-0.72483358261382	6.74536593867121	2.13838839711046
C	-6.29353371260364	1.37635084994172	-2.58899234378295
C	-0.56087557870662	-7.31932534255799	2.25276451619181
C	-7.07182825036597	0.22561658746035	-2.62265276627171
H	0.60652811998184	0.93464716194733	-2.17026377724295
H	-0.56865059559046	-0.92591767903644	-2.18301886275182
H	0.77066971460762	1.52519513068293	-3.84287241378868
H	6.53092737014778	0.91749140677127	2.27379790853862
H	6.00010791946486	2.51902006024789	0.24231723984640
H	4.37153655550166	2.41713852318755	-0.44323822254253
H	4.87293289435018	-0.39824608985797	4.02970814888568
H	1.33951826230806	-0.04462243106667	2.61409222181707
H	2.11429378498466	-0.21585426012609	4.21165729027830
H	2.90204258476867	3.94874112635800	0.94461319481678
H	3.39186406834412	5.44706954587924	0.14125396874680
H	2.10570540814912	5.99596195274025	-2.14932983893751
H	0.85576838060794	4.32113791251246	-3.93793515348303
H	-0.70549254520981	-1.49956944132832	-3.86393014430979
H	-1.18844665609041	2.28737880417300	-5.07034453617661
H	2.87528228874175	-2.41286872430651	4.98459504884061
H	2.91101931647551	-4.92265101915004	3.85526730802910
H	2.99342622732164	-5.61742074283249	1.32074373235814
H	2.22323153595456	-4.51343847147588	0.16086321994755
H	1.27278544592948	-2.24396258357387	-5.06947958114518
H	3.88300269976250	-2.75727139033860	-4.35810689074096
H	4.82529384202909	-3.48334107948050	-1.99419816072272
H	4.02961673310697	-2.76618505638544	-0.57738269868978
H	-3.37743197039649	-5.46736173455621	0.03975942529449
H	-2.90692971924729	-3.97394440376776	0.86359548973124
H	-2.04680881909596	-5.99321229213251	-2.23222498634029
H	-0.77345074949020	-4.29847988562276	-3.98471293279744
H	-6.55898819462447	-0.93090465991796	2.16188389610743
H	-4.36058606888149	-2.41653214244285	-0.53289275328622
H	-6.00051145037535	-2.52113101312792	0.12533282659905
H	-4.92329468191890	0.36359144457373	3.95416440859079
H	-1.36949538077575	0.00778254158754	2.59060762078506
H	-2.16775786004506	0.16745694705317	4.17773767110155
H	-2.93318945017473	2.35829993570856	4.95908654501505
H	-2.95042605266914	4.87829722831654	3.85195585296237
H	-2.99987616342287	5.59458664650707	1.32264458742951
H	-2.21222241907469	4.50150444860008	0.16418083945812
H	-3.80767422677936	2.79878972956906	-4.39200003410460

H	-4.00849508206297	2.76829663730001	-0.61314194559834
H	-4.78412913701004	3.50098617340931	-2.03299924457139
H	7.35347177910272	1.76327453787438	-1.66556046922931
H	2.62631660507185	7.07608000605510	1.83083329498006
H	8.00470181101337	-0.14015989381369	-3.13358722263248
H	0.67022204409827	8.20608422508986	2.87980613994337
H	1.57340664175483	-7.25453295661315	2.52854220177149
H	6.61518744306947	-2.20850094929398	-3.10719337065250
H	-2.63944426877395	-7.10074830433021	1.73801562633317
H	-7.32094728267440	-1.74629249338580	-1.79719120591320
H	-1.59844381948778	7.22782805477319	2.55637084876084
H	-6.55739719709703	2.23915289204268	-3.18628305138047
H	-0.69960485817406	-8.23456770216205	2.81268571431816
H	-7.94773352127822	0.17270003170099	-3.25542959056390

References

(1) Liu, Y.; Resch, S.; Chen, H.; Dechert, S.; Demeshko, S.; Bill, E.; Ye, S.; Meyer, F. Fully Delocalized Mixed-Valent $Cu^{1.5}Cu^{1.5}$ Complex: Strong Cu-Cu Interaction and Fast Electron Self-Exchange Rate Despite Large Structural Changes. *Angew. Chem., Int. Ed.* **2023,** *62*, e202215840. doi: 10.1002/anie.202215840
(2) Nesmeyanov, A. N.; Materikova, R. B.; Lyatifov, I. R.; Kurbanov, T. K.; Kochetkova, N. S. *Sym*-Polymethylferricinium Hexafluorophosphates. *J. Organomet. Chem.* **1978,** *145*, 241-243. doi: 10.1016/s0022-328x(00)91129-x
(3) Sheldrick, G. M. Shelxt - Integrated Space-Group and Crystal-Structure Determination. *Acta Cryst.* **2015,** *A71*, 3-8. doi: 10.1107/S2053273314026370
(4) Sheldrick, G. M. Crystal Structure Refinement with Shelxl. *Acta Cryst.* **2015,** *C71*, 3-8. doi: 10.1107/S2053229614024218
(5) *X-Red; STOE & CIE GmbH, Darmstadt, Germany*. 2002.
(6) *SADABS; BRUKER AXS GmbH, Karlsruhe, Germany*. 2016.
(7) Spek, A. L. Platon Squeeze: A Tool for the Calculation of the Disordered Solvent Contribution to the Calculated Structure Factors. *Acta Cryst.* **2015,** *C71*, 9-18. doi: 10.1107/S2053229614024929
(8) Bill, E. j*ulX, Program for Simulation of Molecular Magnetic Data, Max-Planck Institute for Chemical Energy Conversion, Mülheim/Ruhr*. 2008.
(9) Stoll, S.; Schweiger, A. Easyspin, a Comprehensive Software Package for Spectral Simulation and Analysis in EPR. *J Magn. Reson.* **2006,** *178*, 42-55. doi: 10.1016/j.jmr.2005.08.013
(10) Neese, F. The Orca Program System. *WIREs Comput. Mol. Sci.* **2012,** *2*, 73-78. doi: 10.1002/wcms.81
(11) Neese, F. Software Update: The Orca Program System, Version 4.0. *WIREs Comput. Mol. Sci.* **2017,** *8*. doi: 10.1002/wcms.1327
(12) Lee, C.; Yang, W.; Parr, R. G. Development of the Colle-Salvetti Correlation-Energy Formula into a Functional of the Electron Density. *Phys. Rev. B* **1988,** *37*, 785-789. doi: 10.1103/physrevb.37.785
(13) Becke, A. D. Density-Functional Thermochemistry. III. The Role of Exact Exchange. *J. Chem. Phys.* **1993,** *98*, 5648-5652. doi: 10.1063/1.464913
(14) Schäfer, A.; Horn, H.; Ahlrichs, R. Fully Optimized Contracted Gaussian Basis Sets for Atoms Li to Kr. *J. Chem. Phys.* **1992,** *97*, 2571-2577. doi: 10.1063/1.463096
(15) Weigend, F.; Ahlrichs, R. Balanced Basis Sets of Split Valence, Triple Zeta Valence and Quadruple Zeta Valence Quality for H to Rn: Design and Assessment of Accuracy. *Phys. Chem. Chem. Phys.* **2005,** *7*, 3297-3305. doi: 10.1039/b508541a
(16) Weigend, F. Hartree-Fock Exchange Fitting Basis Sets for H to Rn. *J. Comput. Chem.* **2008,** *29*, 167-175. doi: 10.1002/jcc.20702
(17) Klamt, A.; Schüürmann, G. Cosmo: A New Approach to Dielectric Screening in Solvents with Explicit Expressions for the Screening Energy and Its Gradient. *J. Chem. Soc., Perkin Trans. 2* **1993,** 799-805. doi: 10.1039/p29930000799
(18) Barone, V.; Cossi, M. Quantum Calculation of Molecular Energies and Energy Gradients in Solution by a Conductor Solvent Model. *J. Phys. Chem. A* **1998,** *102*, 1995-2001. doi: 10.1021/jp9716997
(19) Eichkorn, K.; Treutler, O.; Öhm, H.; Häser, M.; Ahlrichs, R. Auxiliary Basis Sets to Approximate Coulomb Potentials. *Chem. Phys. Lett.* **1995,** *242*, 652-660. doi: 10.1016/0009-2614(95)00838-u
(20) Neese, F.; Wennmohs, F.; Hansen, A.; Becker, U. Efficient, Approximate and Parallel Hartree–Fock and Hybrid DFT Calculations. A 'Chain-of-Spheres' Algorithm for the Hartree–Fock Exchange. *Chem. Phys.* **2009,** *356*, 98-109. doi: 10.1016/j.chemphys.2008.10.036
(21) Grimme, S.; Antony, J.; Ehrlich, S.; Krieg, H. A Consistent and Accurate Ab Initio Parametrization of Density Functional Dispersion Correction (DFT-D) for the 94 Elements H-Pu. *J. Chem. Phys.* **2010,** *132*, 154104. doi: 10.1063/1.3382344

(22) Mori, R. A.; Paris, E.; Giuli, G.; Eeckhout, S. G.; Kavcic, M.; Zitnik, M.; Bucar, K.; Pettersson, L. G.; Glatzel, P. Sulfur-Metal Orbital Hybridization in Sulfur-Bearing Compounds Studied by X-Ray Emission Spectroscopy. *Inorg. Chem.* **2010,** *49*, 6468-6473. doi: 10.1021/ic100304z
(23) Hölzer, G.; Fritsch, M.; Deutsch, M.; Härtwig, J.; Förster, E. K$\alpha_{1,2}$ and K$\beta_{1,3}$ X-Ray Emission Lines of The 3*d* Transition Metals. *Phys. Rev. A* **1997,** *56*, 4554-4568. doi: 10.1103/PhysRevA.56.4554
(24) DeBeer George, S.; Petrenko, T.; Neese, F. Prediction of Iron *K*-Edge Absorption Spectra Using Time-Dependent Density Functional Theory. *J. Phys. Chem. A* **2008,** *112*, 12936-12943. doi: 10.1021/jp803174m
(25) Lee, N.; Petrenko, T.; Bergmann, U.; Neese, F.; DeBeer, S. Probing Valence Orbital Composition with Iron Kβ X-Ray Emission Spectroscopy. *J. Am. Chem. Soc.* **2010,** *132*, 9715-9727. doi: 10.1021/ja101281e
(26) Stoychev, G. L.; Auer, A. A.; Neese, F. Automatic Generation of Auxiliary Basis Sets. *J. Chem. Theory Comput.* **2017,** *13*, 554-562. doi: 10.1021/acs.jctc.6b01041
(27) van Lenthe, E.; Wormer, P. E. S.; van der Avoird, A. Density Functional Calculations of Molecular *g*-Tensors in the Zero-Order Regular Approximation for Relativistic Effects. *J. Chem. Phys.* **1997,** *107*, 2488-2498. doi: 10.1063/1.474590
(28) Pantazis, D. A.; Chen, X. Y.; Landis, C. R.; Neese, F. All-Electron Scalar Relativistic Basis Sets for Third-Row Transition Metal Atoms. *J. Chem. Theory Comput.* **2008,** *4*, 908-919. doi: 10.1021/ct800047t
